# Identifying Therapeutic Agents for Amelioration of Mitochondrial Clearance Disorder in Neurons of Familial Parkinson Disease

**DOI:** 10.1016/j.stemcr.2020.04.011

**Published:** 2020-05-28

**Authors:** Akihiro Yamaguchi, Kei-ichi Ishikawa, Tsuyoshi Inoshita, Kahori Shiba-Fukushima, Shinji Saiki, Taku Hatano, Akio Mori, Yutaka Oji, Ayami Okuzumi, Yuanzhe Li, Manabu Funayama, Yuzuru Imai, Nobutaka Hattori, Wado Akamatsu

**Affiliations:** 1 Center for Genomic and Regenerative Medicine, Juntendo University Graduate School of Medicine, Tokyo 113-8431, Japan; 2 Department of Neurology, Juntendo University School of Medicine, Tokyo 113-8431, Japan; 3 Department of Treatment and Research in Multiple Sclerosis and Neuro-intractable Disease, Tokyo 113-8431, Japan; 4 Research Institute for Diseases of Old Age, Graduate School of Medicine, Juntendo University, Tokyo 113-8421, Japan; 5 Department of Research for Parkinson's Disease, Juntendo University Graduate School of Medicine, Tokyo 113-8431, Japan

**Keywords:** Parkinson disease, iPS cells, mitochondria, drug screening, PARK2, PARK6, mitophagy

## Abstract

Parkinson disease (PD) is a neurodegenerative disorder caused by the progressive loss of midbrain dopaminergic neurons, and mitochondrial dysfunction is involved in its pathogenesis. This study aimed to establish an imaging-based, semi-automatic, high-throughput system for the quantitative detection of disease-specific phenotypes in dopaminergic neurons from induced pluripotent stem cells (iPSCs) derived from patients with familial PD having *Parkin* or *PINK1* mutations, which exhibit abnormal mitochondrial homeostasis. The proposed system recapitulates the deficiency of mitochondrial clearance, ROS accumulation, and increasing apoptosis in these familial PD-derived neurons. We screened 320 compounds for their ability to ameliorate multiple phenotypes and identified four candidate drugs. Some of these drugs improved the locomotion defects and reduced ATP production caused by PINK1 inactivation in *Drosophila* and were effective for idiopathic PD-derived neurons with impaired mitochondrial clearance. Our findings suggest that the proposed high-throughput system has potential for identifying effective drugs for familial and idiopathic PD.

## Highlights

• Semi-automatic phenotypic analysis system for familial PD-iPSC-derived neurons
• Library screening identified four compounds that improved mitochondrial clearance
• Compounds improved multiple phenotypes in fly PD model and idiopathic PD neurons
• Drugs screened in this system could be effective for familial and idiopathic PD

## Graphical Abstract

**Figure:**
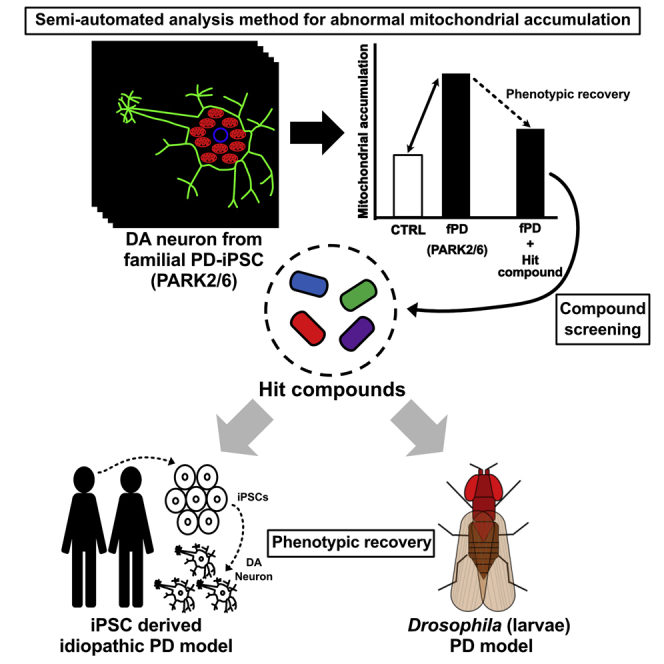

## Teaser

Wado Akamatsu and colleagues established an impaired mitochondrial clearance detection system for familial Parkinson disease (PD) iPSC-derived neurons and identified four potential therapeutic agents. Some of the agents exerted multiple phenotypic recovery effects *in vivo* (*Drosophila* PD model) and *in vitro* (familial and sporadic PD neurons). This system has the potential to identify novel disease-modifying agents for PD, including sporadic cases.

## Introduction

Parkinson disease (PD) is the second most common neurodegenerative disorder and is caused by the progressive loss of midbrain dopaminergic neurons (Kalia and Lang, 2015). Currently, its pharmacologic treatment is aimed primarily at correcting dopamine insufficiency. However, an effective disease-modifying therapy has yet to be established.

To date, more than 20 monogenic causative genes and numerous genetic risk factors have been identified (Deng et al., 2018). Familial cases with monogenic alterations comprise only a small percentage (of up to 10%) of PD patients, whereas the majority are idiopathic cases without any familial history (Tysnes and Storstein, 2017). Functional analyses of causative genes in familial PD suggest that various pathomechanisms, such as mitochondrial dysfunction, oxidative stress, α-synuclein accumulation, and impaired proteolysis, including macroautophagy and the ubiquitin proteasome pathway, underlie the dopaminergic neuronal loss in patients affected by PD (Kalia and Lang, 2015). Recent studies have suggested that mitochondrial dysfunction is a key factor in its pathophysiology. Mitochondrial dysfunctions have been reported in *Parkin* (PARK2), *PINK1* (PARK6)-, *DJ-1* (PARK7)-, *LRRK2* (PARK8)-, and *CHCHD2* (PARK22)-linked familial cases (Park et al., 2018), and several risk variants of these genes have been identified in idiopathic cases of PD (Kalia and Lang, 2015, Nalls et al., 2014). Moreover, the postmortem brain analysis of patients with idiopathic PD revealed mitochondrial dysfunctions, thereby suggesting their close association with the pathogenesis of idiopathic PD (Devi et al., 2008, Schapira et al., 1990, Sian et al., 1994). Therefore, identifying the therapeutic candidates for restoring impaired mitochondrial functions in PD could facilitate drug discovery for both familial and idiopathic PD.

PARK2 is the most common autosomal recessive (AR) form of early-onset PD (Lücking et al., 2000) caused by homozygous mutations in the *Parkin* gene. Mutations of the *PINK1* gene induce the second most frequent AR familial PD, named as PARK6. Clinicopathological phenotypes of these disorders in human and animal models are quite similar (Takanashi et al., 2016, Valente et al., 2004). In addition, it has been reported that Parkin is required for mitochondrial quality control, working closely with PINK1 protein kinase (Matsuda et al., 2010, Narendra et al., 2010). We and others have reported that the removal of damaged mitochondria in mitophagy is impaired in neurons derived from PARK2- and PARK6-induced pluripotent stem cells (iPSCs) after the accumulation of oxidative stress, thereby resulting in neuronal cell death (Imaizumi et al., 2012, Lahiri and Klionsky, 2017, Shiba-Fukushima et al., 2017).

In this study, we established an imaging-based, semi-automatic, high-throughput assay system for detecting both the cell viability and the impaired mitochondrial clearance in PARK2 (*Parkin*-Ex2-4 homozygous deletion and *Parkin*-Ex6, 7 homozygous deletion) and PARK6 (*PINK1*-c.1162T>C homozygous mutation) patient-derived dopaminergic neurons, aiming to screen potential therapeutic drugs that improve mitochondrial dysfunction in PARK2/6 neurons. We used PARK2/6 iPSCs to screen 320 compounds and identified 4 that improved the pathogenetic phenotypes in dopaminergic neurons. We then verified the therapeutic effects of these drug candidates using a *Drosophila* PD model, as well as iPSCs derived from patients with idiopathic PD. The results suggest that our proposed high-throughput phenotype detection system for PARK2/6 neurons is an effective drug-screening platform for isolating therapeutic agents that can restore impaired mitochondrial clearance in PD.

## Results

### High-Throughput Phenotype Detection of PARK2 and PARK6 iPSC-Derived Neurons

We have previously observed via immunofluorescent imaging that neurons differentiated from PARK2 and PARK6 iPSCs showed mitochondrial accumulation caused by impaired mitochondrial clearance (Imaizumi et al., 2012, Shiba-Fukushima et al., 2017). This phenotype is a fundamental pathomechanism of PD, including idiopathic cases. Therefore, we sought to increase the throughput of this method for applications to drug discovery and a large-scale cohort of PD-iPSC studies.

**Figure 1:**
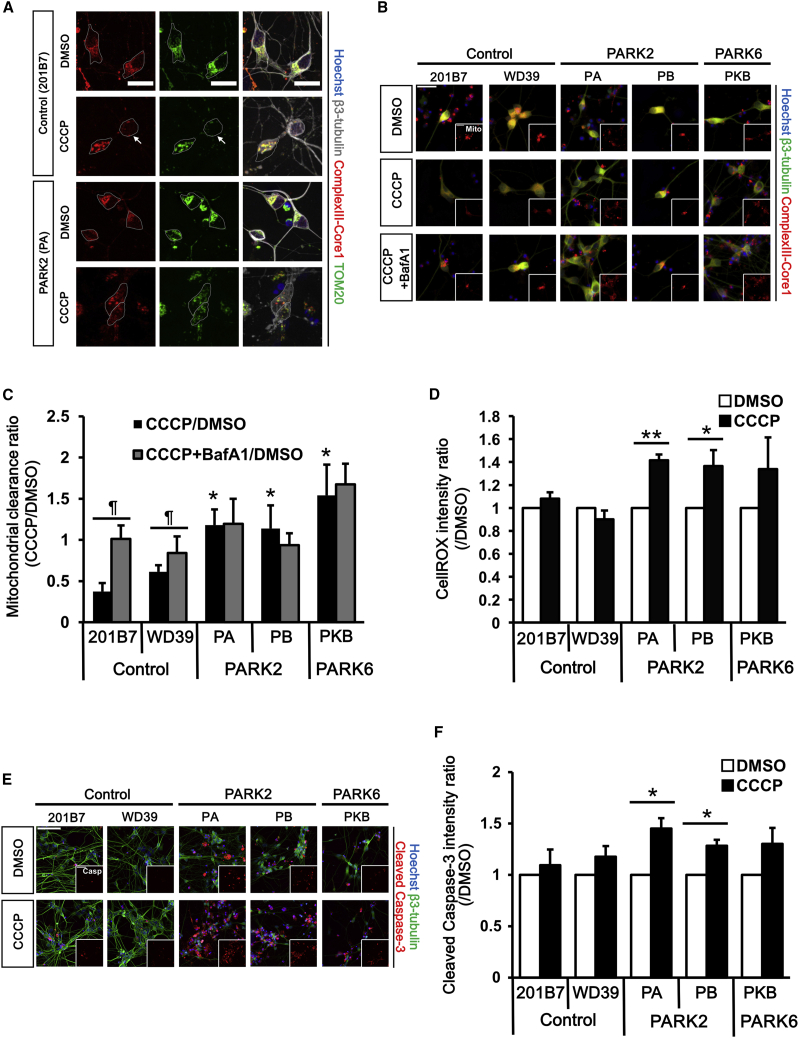
Establishment of a High-Throughput Phenotype Detection System (A) Immunostaining of control and PARK2 neurons with antibodies against mitochondrial proteins (ComplexⅢ-Core1 and TOM20) and a neuronal marker (β3-tubulin). Gray dotted lines indicate neuron cell bodies. Mitochondria are eliminated in the CCCP-treated control neuron (arrows). Scale bar, 20 μm. (B) Representative images of the mitochondrial clearance assay. Scale bar, 20 μm. (C) Quantitative data of the mitochondrial clearance assay. The mitochondrial area was reduced in day 32 control neurons treated with CCCP but not in day 32 PARK2 (PA and PB) and PARK6 (PKB) neurons. Data represent the ratio of mitochondrial area in neurons treated with CCCP (30 μM)/BafA1 (5 μM) and that in neurons treated with DMSO (n = 4 independent replicates; mean ± SEM). ^∗^p < 0.05 compared with 201B7, ¶p < 0.05 compared between CCCP treatment and CCCP + BafA1 treatment by Wilcoxon rank sum test. (D) ROS accumulation assay. Data represent the ratio of fluorescent intensity of day 32 CCCP-treated neurons and that of DMSO-treated neurons (n = 5 independent replicates; mean ± SEM). ^∗^p < 0.05, ^∗∗^p < 0.01 compared with DMSO by Wilcoxon rank sum test. (E) Representative images of the cell-viability assay. Scale bar, 100 μm. (F) Quantitative data of the cell-viability assay. Data represent the ratio of fluorescence intensity of cleaved caspase-3 in day 32 CCCP-treated neurons and that in day 32 DMSO-treated neurons (n = 5 independent replicates; mean ± SEM). ^∗^p < 0.05 compared with DMSO by Wilcoxon rank sum test. BafA1, bafilomycin A1; CCCP, carbonyl cyanide 3-chlorophenylhydrazone. See also Figure S1.

To establish an efficient analysis system for the monitoring of the PD-specific phenotypes of iPSC-derived neurons and for a large-scale drug screening, we first improved the method for neural differentiation. iPSCs were treated with SB431542 (transforming growth factor β3 [TGF-β] receptor inhibitor), dorsomorphin (AMPK inhibitor), and CHIR99021 (Wnt signal activator) for 5 days to induce embryoid body-like state (CTraS) cells to accelerate differentiation (Fujimori et al., 2017). These cells were then differentiated into neurospheres with region specificity of ventral midbrain by adding CHIR99021 and purmorphamine (Hedgehog signal activator) for 17 days as described previously (Imaizumi et al., 2015). Subsequently, the neurospheres were dissociated and plated onto 96-well plates for 10 days to induce neurons (Figure S1A). We confirmed that the neurospheres and the neurons differentiated with CHIR99021 and purmorphamine expressed midbrain markers (FOXA2, LMX1A, GIRK2, and NURR1) and a dopaminergic neuron marker (tyrosine hydroxylase [TH]) as seen in Figures S1B and S1C. Then, iPSC-derived dopaminergic neurons were treated by 30 μM carbonyl cyanide 3-chlorophenylhydrazone (CCCP), a mitochondrial membrane potential uncoupler, to induce mitochondrial elimination as described previously (Imaizumi et al., 2012) (Figure 1A).

To establish a drug-screening system using impaired mitochondrial clearance in PD iPSCs, we used two healthy controls, two PARK2 (*Parkin*-Ex2-4 homozygous deletion; PA and *Parkin*-Ex6, 7 homozygous deletion; PB), and one PARK6 (*PINK1*-c.1162T>C heterozygous mutation; PKB) iPSCs to derive dopaminergic neurons. After immunostaining with anti-β3-tubulin, complexIII-core1, cleaved caspase-3, and TH (Figure S1D), we confirmed that the differentiation efficiencies into dopaminergic neurons and the amounts of mitochondria at basal condition were not different among all the iPSCs clones (Figures S1E and S1F).

Figure S2 shows the schematic of the proposed high-throughput phenotype detection system. All images of neurons were obtained automatically using the imaging cytometer (IN Cell Analyzer 2200), while the subsequent recognition and quantification of the mitochondrial area in neurons were analyzed automatically by the imaging analyzer (IN Cell Developer Toolbox). Using this system, we detected that the mitochondrial area was unchanged in 30 μM CCCP-treated PARK2 and PARK6 neurons, but was significantly decreased in CCCP-treated control neurons (Figures 1B and 1C), which is consistent with our previous observation (Imaizumi et al., 2012). We confirmed that the mitochondrial area reduction in CCCP-treated control neurons was attenuated by 5 μM bafilomycin A1 (BafA1, a V-ATPase inhibitor), confirming that the reduced area was caused by lysosomal degradation. (Figures 1B and 1C). Similar results were obtained in PARK2 and PARK6 neurons treated with a different mitochondrial uncoupler, rotenone, and/or the lysosomal inhibitors, E64d and pepstatin A (Figure S1G). These data suggest that the proposed imaging-based, high-throughput system is capable of detecting disease-specific phenotypes caused by impaired lysosomal degradation of damaged mitochondria in PARK2 and PARK6 neurons within a differentiation culture period of 32 days, which is a 1/3 times faster than conventional methods.

We then tested this system to detect other phenotypes of PARK2 and PARK6 neurons. By using CellROX, a reactive oxygen species (ROS) indicator, we evaluated the oxidative stress in PARK2 and PARK6 neurons. ROS accumulation due to mitochondrial dysfunction was observed in CCCP-treated PARK2 neurons (Figure 1D) as reported previously (Fujimori et al., 2017). Increased activation of caspase-3 in PARK2 neurons was also detected, suggesting that these neurons were sensitive to cell death (Figures 1E and 1F). Similarly, both ROS and cleaved caspase-3 signals tended to be higher in PARK6 neurons, but these differences were not statistically significant. These results suggest that our proposed method evaluated mitochondrial stress and apoptosis level of iPSC-derived neurons to some extent within 32 days.

### Screening for Compounds that Improve Mitochondrial Clearance and Cell Viability in PARK2 and PARK6 iPSC-Derived Neurons

**Figure 2:**
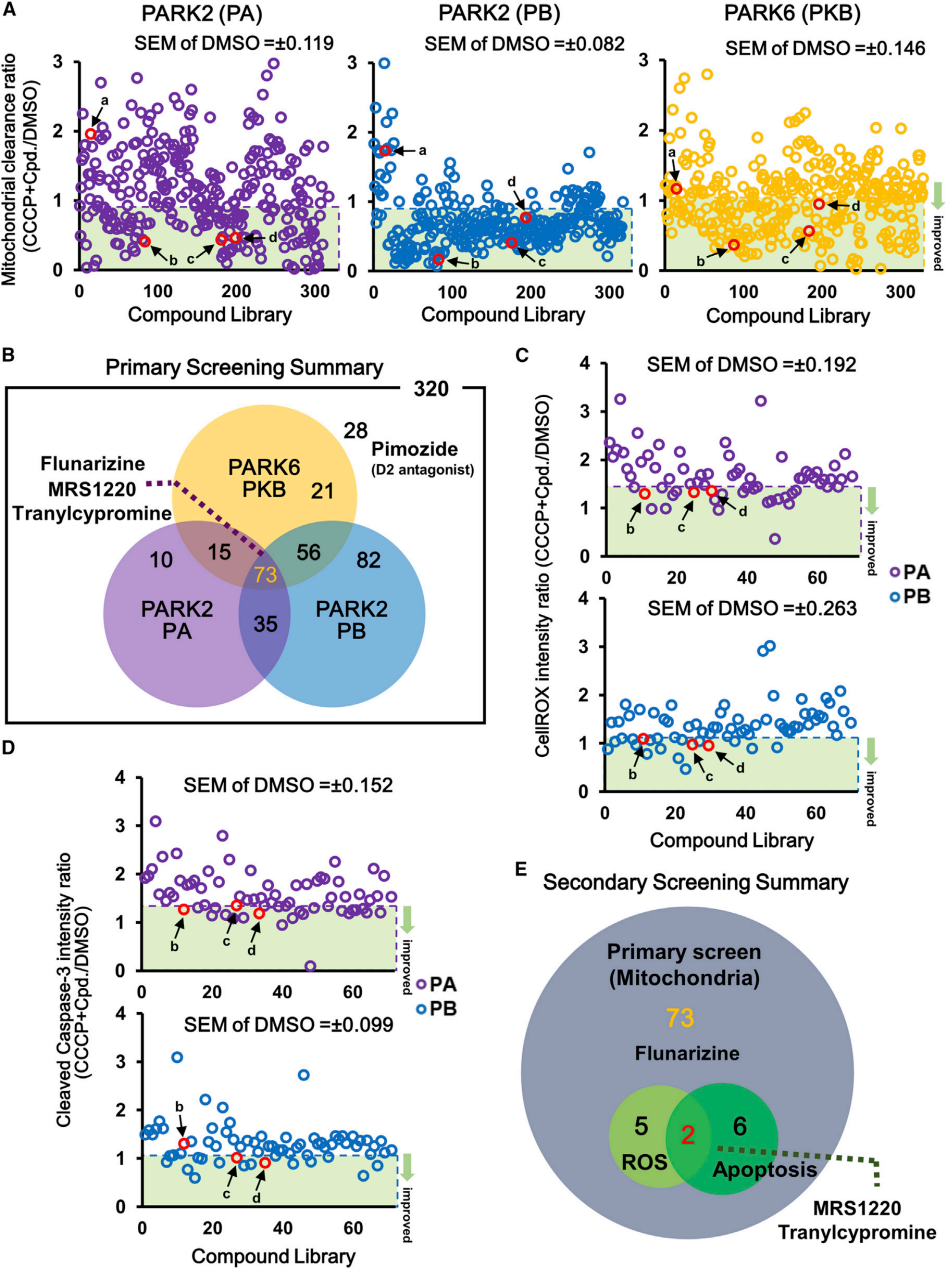
Screening for Compounds that Modify Mitochondrial Clearance in PARK2 and PARK6 Neurons (A) A scatterplot of mitochondrial clearance screening for PARK2 and PARK6 neurons. Data represent the ratio of mitochondrial area in neurons treated with CCCP and compound (Cpd) and that in neurons treated with DMSO. Hit compounds (below the average value of CCCP + DMSO treatment minus SEM) are indicated by the green band. Four notable compounds are indicated by the red frame. The average value ± SEM of CCCP + DMSO treatment: PA 1.06 ± 0.119, PB 1.00 ± 0.082, PKB 1.19 ± 0.146. (B) Summary of the mitochondrial clearance screening (primary screening). (C) A scatterplot of ROS accumulation screening for PARK2 neurons. Data represent the ratio of fluorescence intensity of CellROX in neurons treated with CCCP and compound and that in neurons treated with DMSO. Hit compounds (below the average value of CCCP + DMSO treatment minus SEM) are indicated by the green band. Three notable compounds are indicated by the red frame. The average value ± SEM of CCCP + DMSO treatment: PA 1.65 ± 0.192, PB 1.42 ± 0.263. (D) A scatterplot of apoptosis screening for PARK2 neurons. Data represent the ratio of fluorescence intensity of cleaved caspase-3 treated with CCCP and compound to that treated with DMSO. Hit compounds (below the average value of CCCP + DMSO treatment minus SEM) are indicated by the green band. Three notable compounds are indicated by the red frame. The average value ± SEM of CCCP + DMSO treatment: PA 1.52 ± 0.152, PB 1.16 ± 0.099. (E) Summary of ROS accumulation and apoptosis screening for PARK2 neurons (secondary screening). CCCP, carbonyl cyanide 3-chlorophenylhydrazone; a, pimozide; b, flunarizine; c, MRS1220; d, tranylcypromine. See also Figure S3.

To verify the applicability of the proposed method to drug screening of PD, we screened 320 pharmacologically active inhibitor compounds for their ability to improve mitochondrial clearance and cell viability in PARK2 and PARK6 iPSC-derived neurons. For the primary screening, the mitochondrial area was evaluated in neurons differentiated from two PARK2-iPSC lines and one PARK6-iPSC line treated with CCCP and 10 μM inhibitor compounds. The candidate drugs were evaluated based on the improvement in mitochondrial clearance by calculating the ratio of the mitochondrial area treated with CCCP and that treated with DMSO or with each compound (Figure 2A). The hit criterion of the screening was defined as less than 1-fold of the absolute values of standard error in the mitochondrial clearance ratio of CCCP + DMSO-treated neurons. We identified 73 compounds that improved the clearance of mitochondria in all clones (Figure 2B). Next, we performed a secondary screening using two types of PARK2 neurons, i.e., PA and PB, in terms of reduced ROS accumulation and decreased cell death (Figures 2C and 2D) because those of PARK6 neurons did not show significant changes. The secondary screening identified two hit compounds, MRS1220, an A3 adenosine receptor (A3A-R) antagonist, and tranylcypromine, a Food and Drug Administration-approved monoamine oxidase (MAO) inhibitor used as an antidepressant, which decreased both ROS generation and apoptosis in PARK2 neurons (Figures 2E and S3A).

A recent study reported that L-type calcium-channel blockers (CCBs), namely benidipine and ML218, exert a neuroprotective effect against increased mitochondrial stress in PARK2/6 iPSC-derived dopaminergic neurons (Tabata et al., 2018). Therefore, we focused on five CCBs, i.e., topiramate, nimodipine, isradipine, zonisamide, and flunarizine, included in the library. As shown in Figures S3B and S3C, only flunarizine (L-, T-, and N-type CCB) was effective in improving mitochondrial clearance. Flunarizine was included in the 73 hit compounds from the primary screening but was excluded in the secondary screening because it was less effective in decreasing ROS levels and cell death. However, because flunarizine was highly effective in improving the mitochondrial clearance ratio in two PARK2 and one PARK6 neurons (Figure S3B), we decided to include it in our subsequent analyses.

Among the 320 compounds screened, 28 were found ineffective in terms of mitochondrial elimination in all 3 lines based on the primary screening (Figure 2B), including pimozide, a dopamine D_2_ receptor antagonist. We further examined whether three D_2_ receptor agonists, i.e., bromocriptine, ropinirole, and aripiprazol, were effective in improving mitochondrial clearance and suppressing cell death in PARK2 and PARK6 neurons. All three D_2_ receptor agonists decreased the mitochondrial area and apoptosis with bromocriptine as the most effective (Figure S3D). Therefore, bromocriptine was included in subsequent analyses.

### Hit Compounds and Their Effects on Mitochondrial Clearance and Apoptosis in Human PARK2 and PARK6 Neurons

**Figure 3:**
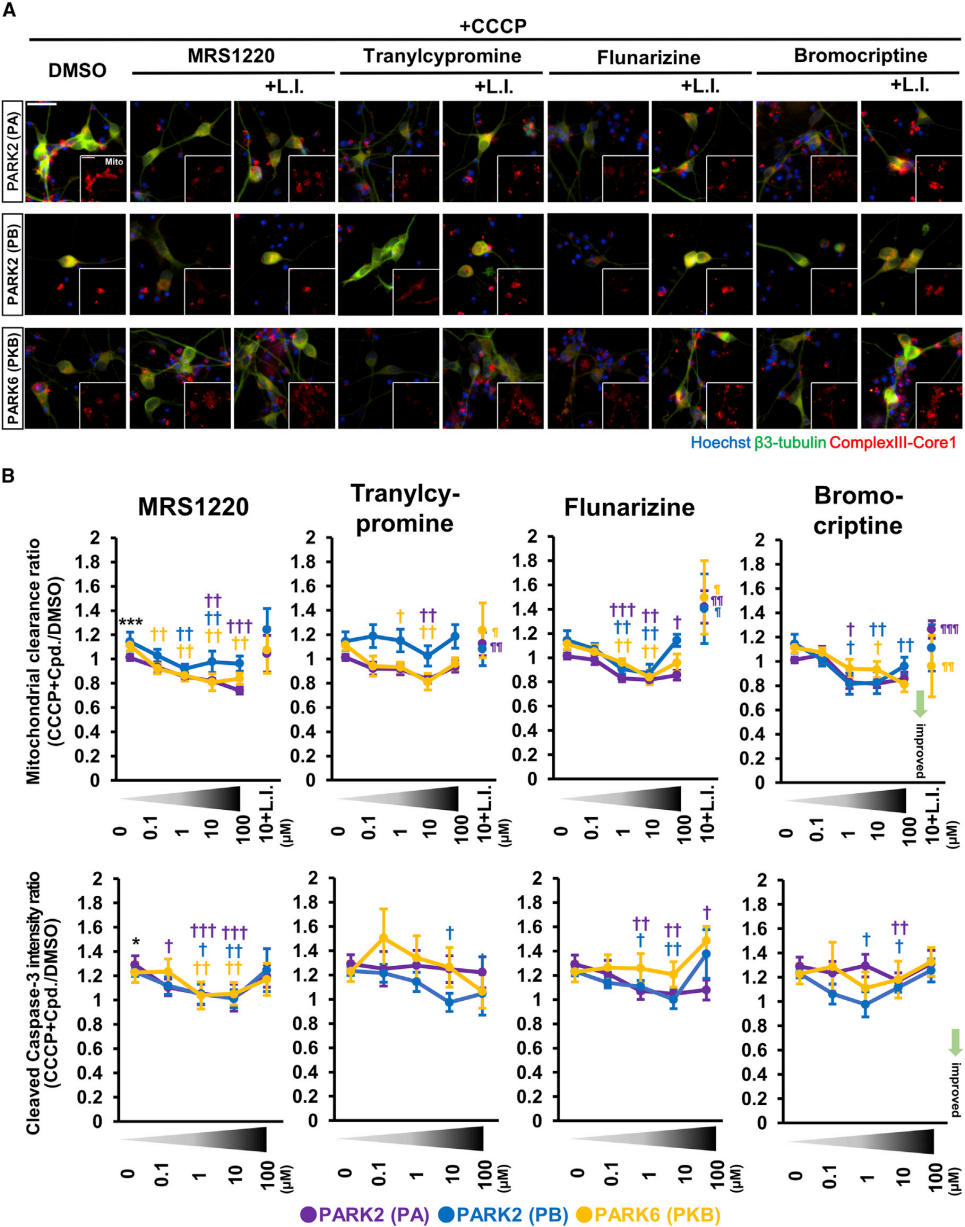
Candidate Compounds Show Reproducibility (A) Images of the mitochondrial clearance analysis for the validation studies of 10 μM candidate compounds. Scale bar, 20 μm. (B) Quantitative data of the candidate compounds in the mitochondrial clearance and apoptosis assays. Plots show the results of DMSO, 0.1–100 μM of each candidate, and 10 μM of candidate with lysosomal inhibitors under CCCP treatment. Data represent the mean ± SEM (n = 10 independent replicates). ^∗^p < 0.05, ^∗∗∗^p < 0.001 compared with DMSO; †p < 0.05, ††p < 0.01 compared with CCCP + DMSO; ¶p < 0.05, ¶¶p < 0.01, ¶¶¶p < 0.001 compared with CCCP + 100 μM compound by Wilcoxon signed rank test. Data represent the mean ± SEM. CCCP, carbonyl cyanide 3-chlorophenylhydrazone; L.I., lysosomal inhibitors (E64d and pepstatin A). See also Figure S4.

To confirm the effect of the four hit compounds, i.e., MRS1220, tranylcypromine, flunarizine, and bromocriptine, on mitochondrial clearance and apoptosis in CCCP-treated neurons, we conducted repetitive tests with 0–100 μM of each compound. As shown in Figures 3 and S4A, all the hit compounds accelerated mitochondrial elimination in a partially dose-dependent manner in PARK2 and PARK6 neurons treated with CCCP. Furthermore, the decrease in the mitochondrial area caused by these compounds was attenuated by lysosomal inhibitors, E64d and pepstatin A (Figures 3A, 3B, and S4A). These results suggested that all four compounds promoted lysosomal degradation of the mitochondria. MRS1220, flunarizine, and bromocriptine exerted anti-apoptotic effects on neurons in a dose-dependent manner with concentrations ranging from 0.1 to 10 μM; however, they seemed to show toxicity at 100 μM. Tranylcypromine showed an anti-apoptotic effect at 100 μM, but its effect on mitochondrial clearance was not significant at this concentration.

To exclude the possibility that the enhanced mitochondrial elimination might be induced by additional mitochondrial damage caused by these four compounds, we examined the mitochondrial membrane potential in neurons treated with 10 μM of the compounds. None of the compounds reduced the mitochondrial membrane potential (Figure S4B), indicating that they were unlikely to induce additional mitochondrial damage. In addition, these compounds did not affect neural differentiation and maturation (Figure S4C). We concluded from these observations that the optimal concentration of all four compounds to be used in subsequent experiments is 10 μM.

**Figure 4:**
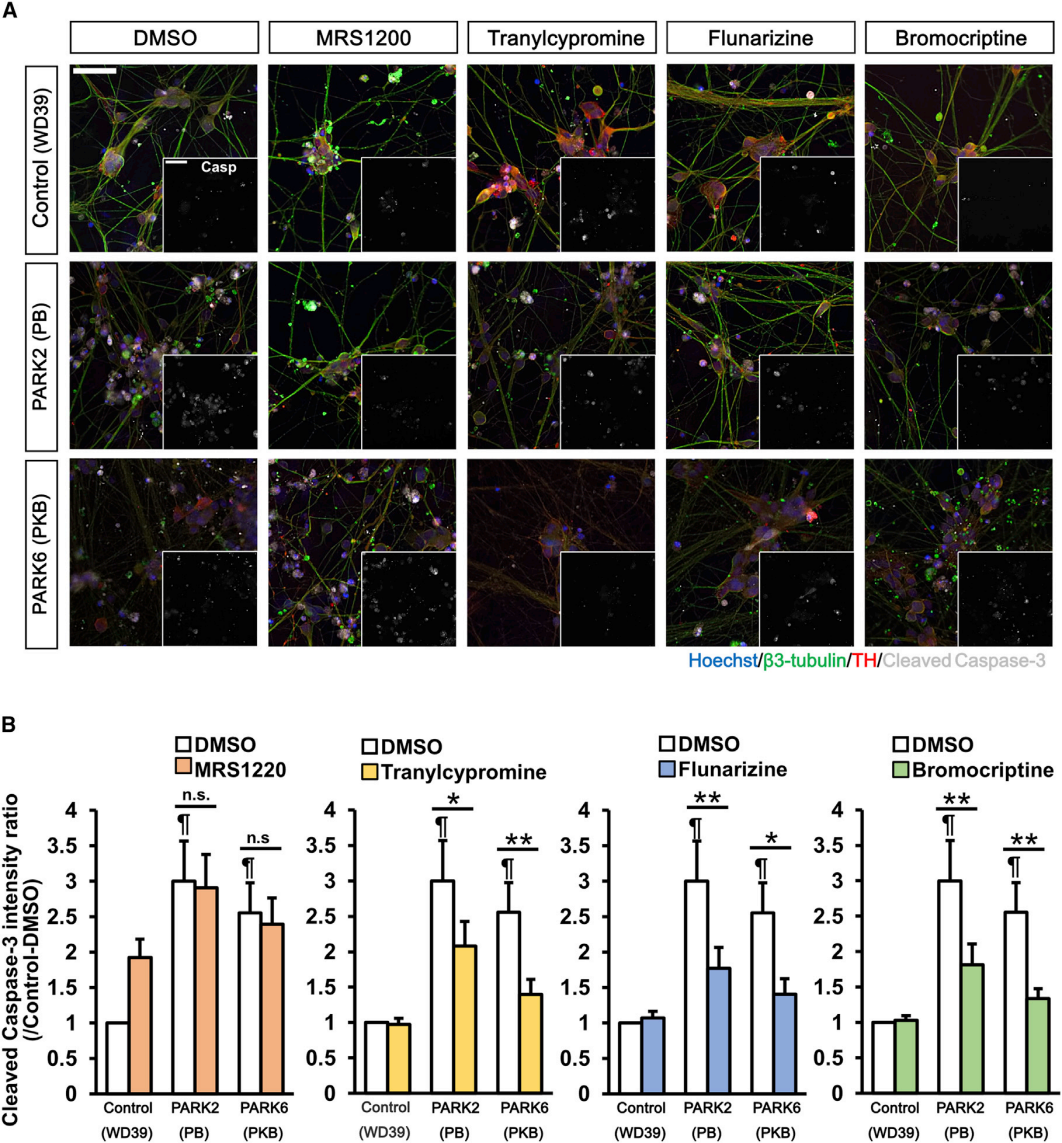
Tranylcypromine, Bromocriptine, and Flunarizine Reduce Spontaneous Apoptosis in PARK2 and PARK6 Neurons (A) Images of day 39 neurons treated with 10 μM compounds for apoptosis analysis in static state. Scale bar, 100 μm. (B) Quantitative data of the apoptosis analysis. Data represent the ratio of fluorescence intensity of cleaved caspase-3 in DMSO-treated control (WD39) neurons and that in 10 μM compound-treated neurons (n = 9 independent replicates; mean ± SEM). ^∗^p < 0.05, ^∗∗^p < 0.01 compared with DMSO; ¶p < 0.05 compared with DMSO-treated control (WD39) neuron by Wilcoxon signed rank test. n.s., not significant.

We next investigated the anti-apoptotic effects of these compounds in PARK2 and PARK6 neurons under a CCCP-untreated static condition. Increased apoptosis in PARK2 and PARK6 neurons without CCCP treatment was not significant by day 32, as identified by the proposed detection system. However, with the addition of culture by day 39, a significant increase in cell death became detectable (Figure S1H). All the hit compounds, except MRS1220, significantly reduced the fluorescence intensity of the cleaved caspase-3 in PARK2 and PARK6 neurons compared with DMSO (Figures 4A and 4B). Although it was not statistically significant, the mean value of the fluorescence intensity of the cleaved caspase-3 was decreased by MRS1220. These results suggest that the hit compounds identified by our high-throughput phenotype detection system could significantly modify multiple phenotypes in PARK2 and PARK6 neurons, thus confirming the validity of the proposed screening method.

### Therapeutic Effects of the Candidate Compounds in a *Drosophila* PD Model

**Figure 5:**
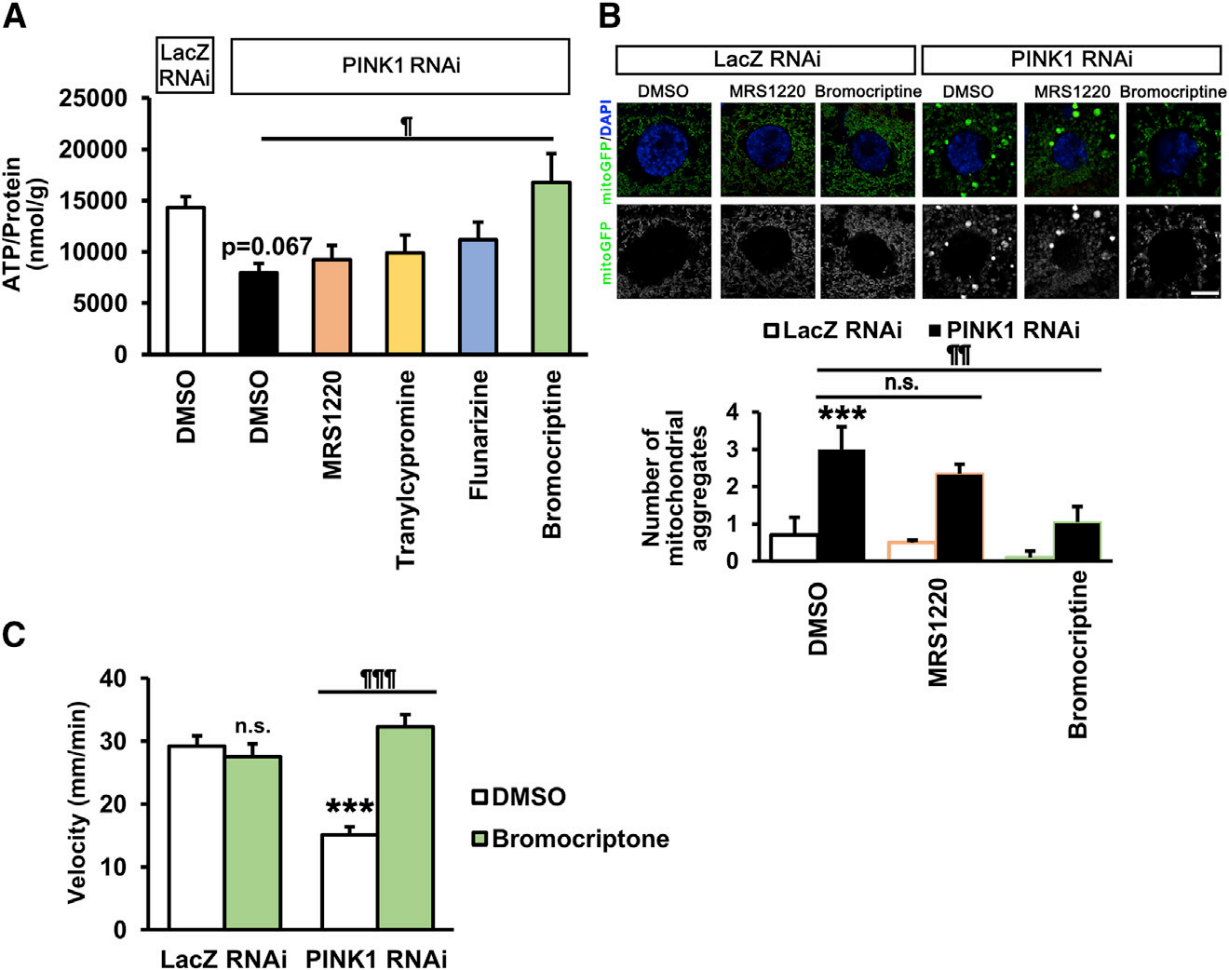
Bromocriptine Rescues Abnormal Phenotypes of *PINK1*-Inactivated *Drosophila* (A) Whole-body ATP levels of *PINK1 RNAi* flies treated with or without drugs. Data represent the mean ± SEM (n = 9 independent replicates). ¶p < 0.05 compared with DMSO in *PINK1 RNAi* by Dunnett's test. (B) Mitochondrial morphology of *PINK1 RNAi* flies treated with or without the indicated drugs. The number of greater than 2 μm^2^ mitochondrial aggregates was graphed. Data represent the mean ± SEM (n = 20 cells from 5 independent flies). ^∗∗∗^p < 0.001 compared with DMSO in *LacZ RNAi*, ¶¶p < 0.01 compared with DMSO in *PINK1 RNAi* by Tukey-Kramer test. n.s., not significant. Scale bar, 10 μm. (C) Bromocriptine did not have any effect on the motor behavior of *LacZ RNAi* flies. Assay was performed as in Figure S5B. Data represent the mean ± SEM (n = 9–35 independent replicates). ^∗∗∗^p < 0.001 compared with DMSO in *LacZ RNAi*, ¶¶¶p < 0.001 compared with DMSO in *PINK1 RNAi* by Tukey-Kramer test. n.s., not significant.

Testing on animal models is an important step in drug discovery. However, mice harboring *PARK2* or *PARK6* mutations do not reproduce the degeneration of midbrain dopaminergic neurons (Goldberg et al., 2003, Itier et al., 2003, Kitada et al., 2007). Indeed, it was difficult to evaluate a PINK1 activation drug, kinetin triphosphate, in *PINK1* mutant mice (Orr et al., 2017). In contrast to rodent models, *PINK1*- or *Parkin*-deficient *Drosophila* exhibit prominent mitochondrial degeneration from an early adult stage (Clark et al., 2006, Park et al., 2006, Yang et al., 2006). Thus, we orally administrated the candidate compounds to *Drosophila* third-instar larvae expressing *PINK1 shRNA* in their muscular tissues (*PINK1*-KD). Although the loss of dopaminergic neurons was hardly detected in this larval stage of *PINK1*-deficient flies, *PINK1*-KD larvae showed apparent locomotion defects, suggesting that the *PINK1*-KD larvae partly reflect the prodromal stage of mitochondria-associated PD (Figures S5A–S5C). In contrast, the larvae expressing control LacZ RNAi (*LacZ*-KD) had normal movements (Figures S5A and S5B). MRS1220 and bromocriptine alleviated the movement disorder of *PINK1*-KD larvae. The other two compounds tended to improve the locomotion defects caused by *PINK1* inactivation (Figure S5B). To verify that the improvements were due to the recovery of mitochondrial functions, we quantified the amount of ATP in larval whole bodies. *PINK1*-KD larvae had lower ATP production than *LacZ-*KD larvae and, among the four candidate compounds, only bromocriptine stimulated the ATP production in *PINK1*-KD larvae (Figure 5A). Mitochondrial aggregation caused by *PINK1* inactivation was also alleviated by bromocriptine but not by MRS1220 (Figure 5B). Moreover, bromocriptine did not affect larval locomotion in *LacZ*-KD larvae (Figure 5C). These results suggest that at least one candidate compound identified by our detection system, i.e., bromocriptine, exerts beneficial effects in *PINK1*-KD flies, thereby showing potential as therapeutic drug for PD.

### Therapeutic Effects of the Candidate Compounds in iPSC-Derived Neurons from Patients with Idiopathic PD

Because idiopathic PD accounts for 90% of PD cases, we further evaluated the therapeutic effects of the four candidate compounds in idiopathic PD iPSC-derived neurons. We generated iPSCs from CD3-positive T lymphocytes derived from four patients with idiopathic PD (iPD1-4) and three age-matched healthy controls (Cont1-3; Table S2). All established iPSC clones showed typical embryonic stem cell-like morphology and were positive for pluripotent markers (Figure S6A). We confirmed no difference in induction efficiency of dopaminergic neurons between the healthy controls and idiopathic PDs (Figures S6B and S6C). To elucidate the idiopathic PD phenotypes and evaluate the effect of the candidate compounds, we examined cell death and mitochondrial clearance abnormalities in neurons derived from each idiopathic iPSC clone as readouts of the appropriate phenotypes.

**Figure 6:**
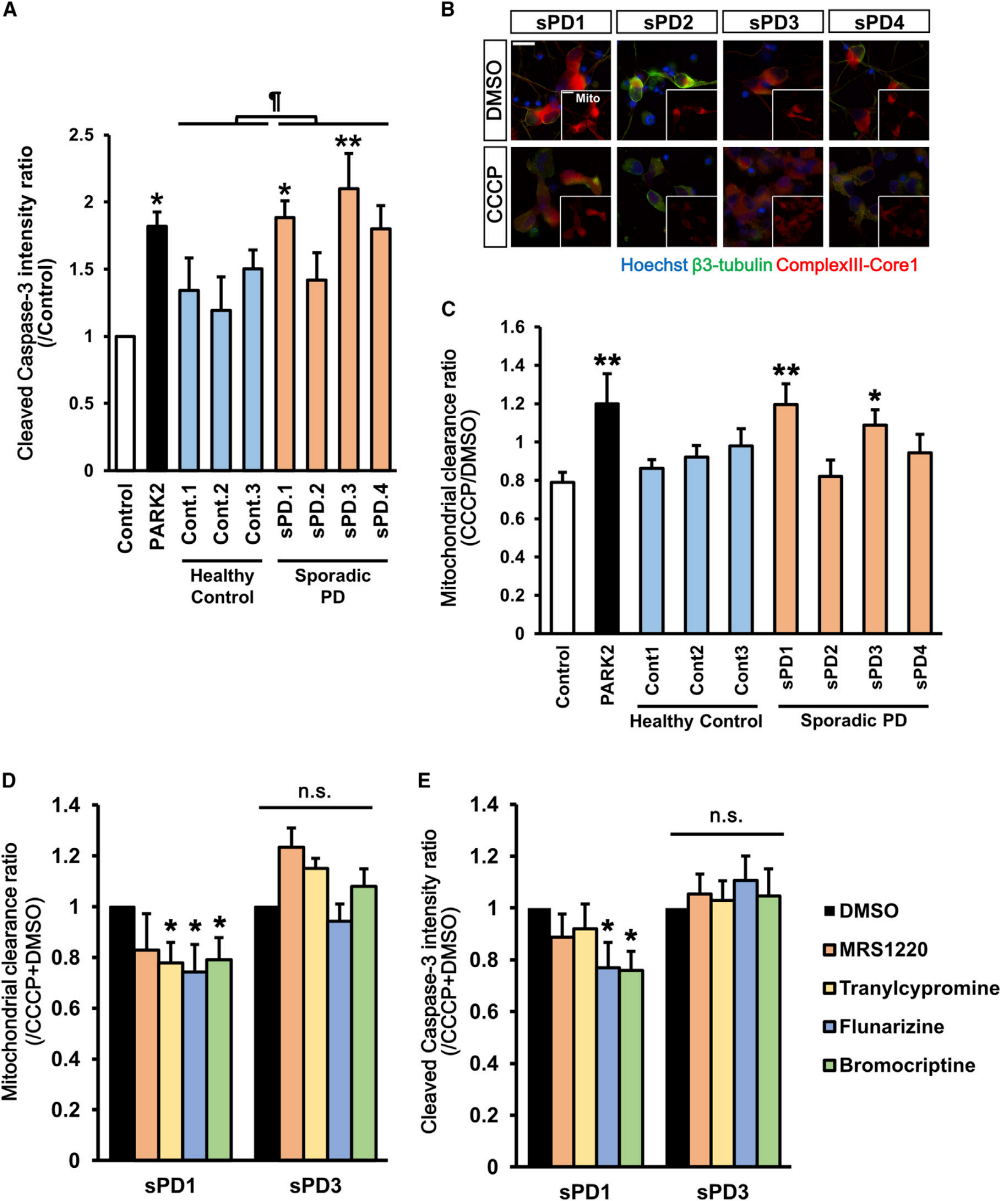
Evaluation of Drug Efficacy in Idiopathic PD-Derived Neurons (A) Apoptosis assay in idiopathic PD-derived neurons. Data represent the ratio of the intensity of cleaved caspase-3 in day 39 idiopathic PD-derived neurons and that in control (201B7) neurons (n = 6–9 independent replicates; mean ± SEM). ^∗^p < 0.05 compared with control neurons by Wilcoxon rank sum test, ¶p < 0.05 compared between Control (Cont) and idiopathic PD (iPD) neurons by Student’s t test. (B) Images of mitochondrial clearance analysis in day 32 neurons. Scale bar, 20 μm. (C) Quantitative data of mitochondrial clearance analysis. Data represent the ratio of mitochondrial area in neurons treated with CCCP and that in neurons treated with DMSO (n = 7–8 independent replicates; mean ± SEM). ^∗^p < 0.05, ^∗∗^p < 0.01 compared with control (201B7) by Wilcoxon rank sum test. (D) Compound evaluation in mitochondrial clearance assay. Data represent the ratio of mitochondrial area in neurons treated with CCCP + compound and that in neurons treated with CCCP + DMSO (n = 6 independent replicates; mean ± SEM). ^∗^p < 0.05 compared with DMSO by Wilcoxon signed rank test. (E) Compound evaluation in apoptosis assay. Data represent the ratio of fluorescence intensity of cleaved caspase-3 in neurons treated with CCCP + compound and that in neurons treated with CCCP + DMSO (n = 6 independent replicates; mean ± SEM). ^∗^p < 0.05 compared with CCCP + DMSO by Wilcoxon signed rank test. n.s., not significant. See also Figure S6.

Quantitation of the cleaved caspase-3-positive neurons using the same protocol as that in familial PDs revealed that the two idiopathic PD lines, iPD1 and iPD3, significantly increased apoptosis compared with the healthy control line WD39 (Figure 6A). Interestingly, only these two lines showed impaired elimination of damaged mitochondria (Figures 6B and 6C). To eliminate the possibility that these iPSC lines have unknown genetic mutations, we analyzed all PD-related genes. Interestingly, two other idiopathic PD lines have LRRK2-p.G2385R (heterozygote), which has been reported as a PD-risk variant (Di Fonzo et al., 2006, Tan et al., 2007). These results suggest that some idiopathic cases recapitulate the phenotypic features of PARK2 and PARK6 neurons.

To evaluate the efficacy of the candidate compounds in idiopathic PD iPSCs, we treated the two idiopathic PD lines, i.e., iPD1 and iPD3, that exhibit impaired mitochondrial clearance and increased apoptosis with these compounds. Tranylcypromine, flunarizine, and bromocriptine significantly improved the impaired mitochondrial clearance in iPD1 line but not in iPD3 (Figure 6D). Flunarizine and bromocriptine also significantly decreased apoptosis in iPD1, but tranylcypromine did not (Figure 6E). These results suggest that bromocriptine is effective for a specific type of idiopathic PD. We conclude that the therapeutic drug candidates identified by our high-throughput phenotype detection system using PARK2/6 iPSCs are effective in other PD models, such as *Drosophila* and idiopathic PD with impaired mitochondrial clearance.

## Discussion

We have established an imaging-based, high-throughput phenotype detection system for neurons derived from PARK2 and PARK6 iPSCs and have assessed its applicability through compound library screening. There have been a few studies that performed drug screening with neurons from iPSCs in neurodegenerative disorders (Imamura et al., 2017, Kondo et al., 2017, Tabata et al., 2018). Because the efficacy of neutron differentiation is critical for the assays, in most studies the neurons were differentiated from iPSCs through the viral expression of neuron-generating genes or through long-term self-renewing neuroepithelial-like stem cells (lt-NES cells). In this study, we have analyzed a large number of iPSC-derived neurons with sufficient induction efficiency in a short culture period by accelerating iPSC differentiation via embryo body-like state by treatment with three chemicals (Fujimori et al., 2017). Moreover, because our automatic analysis system is based on immunofluorescent imaging, we can select the neurons by markers. Because cell culturing and immunostaining are still performed manually, our proposal can be considered a “semi”-automatic high-throughput assay system. This system can recapitulate disease-specific phenotypes similar to previous reports in the same control and familial PD iPSC-derived neurons (Imaizumi et al., 2012, Shiba-Fukushima et al., 2017). Because we focused on the reproducibility of the phenotype detection, we built this system using the same iPSCs, but it might have been better to use other iPSCs as normal controls, such as age-matched or mutation-corrected iPSCs. However, this system managed to detect disease phenotypes in some idiopathic PD and control iPSCs that were not used for drug screening.

We investigated whether drugs identified using iPSC-derived neurons with abnormal mitochondrial clearance were also effective for the majority of patients with PD, including those with idiopathic cases and presumed similar mitochondrial abnormalities. We focused mainly on the phenotype of the mitochondrial clearance abnormality and identified four drugs, i.e., MRS1220, tranylcypromine, flunarizine, and bromocriptine. Mitochondrial quality control is regulated by both the removal of the damaged mitochondria and mitochondrial biogenesis (Pickles et al., 2018). The damaged mitochondria are mainly eliminated by mitophagy, but they can also be degraded by the other processes, such as macroautophagy and the ubiquitin proteasome system (Pickles et al., 2018, Yoshii et al., 2011). In this study, the involvement of lysosomes in the degradation of mitochondria was confirmed, but we did not examine in detail how mitochondria are degraded and how the candidate drugs promote the degradation of mitochondria. Both an A3A-R antagonist, which is reversine, and a histone lysine-specific demethylase 1 inhibitor, which is also a MAO inhibitor, activated autophagy via Akt/mTORC1 inhibition in several cell lines (Ambrosio et al., 2017, Lee et al., 2012); hence, MRS1220 and tranylcypromine may have eliminated mitochondria by activating autophagy. Ca^2+^ signaling is a well-known apoptosis and autophagy regulator. However, the effects of flunarizine on autophagy or apoptosis have not yet been elucidated; therefore, further experiments are required for elucidating the mechanisms of the effects of the candidate drugs. Moreover, mitochondrial dysfunction plays a pivotal role in the pathogenesis of other neurodegenerative diseases, such as Alzheimer disease (Fang et al., 2019), Huntington disease, and amyotrophic lateral sclerosis (Rogers et al., 2017). Therefore, our proposed system could also be useful in detecting disease phenotypes and screening therapeutic drugs in these neurodegenerative diseases.

Considering the clinical use of these drugs for treatment of PD, TCP-FA4, a derivate of tranylcypromine (Desino et al., 2009), flunarizine (Agarwal et al., 1996), and bromocriptine (Friis et al., 1979), have good blood-brain barrier permeability. Caffeine, a nonselective adenosine receptor antagonist, has been reported to lower the risk of PD (Altman et al., 2011, Postuma et al., 2012). Currently, an adenosine A2A receptor antagonist is used as treatment for PD, but A3A-R antagonists have not yet been used. Tranylcypromine is used as an antidepressant, and other MAO inhibitors are currently used as a treatment for PD. Further experiments are needed to determine whether this effect on mitochondria is unique to tranylcypromine. Flunarizine is effective in treating migraine but has side effects, such as depression and weight gain (Sørensen et al., 1991). CCBs can induce parkinsonism (Jhang et al., 2017), but some reports suggested that CCBs lower the risk of PD (Jhang et al., 2017, Simon et al., 2010, Swart and Hurley, 2016). The ergot-derived dopamine agonists were commonly used as a treatment for PD, but lots of adverse events, such as valvar heart disease, retroperitoneal fibrosis, pleurisy, and pericarditis have been reported (Horvath et al., 2004, Schade et al., 2007). Moreover, because we used these candidate compounds as treatment to neurons and flies in progressive phase, their efficacies can be expected from the prodromal to mid-term stage of PD. Overall, these four compounds have potential as disease-modifying treatment for PD, and the elucidation of their mechanisms of action, including their target molecules and pathways, could lead to the discovery of novel clinically optimized drugs.

The efficacy of the candidate drugs was validated using both *in vivo* and *in vitro* models as summarized in Table 1. We first confirmed their efficacies and optimal concentrations in PARK2 and PARK6 iPSCs. Then we applied them to *PINK1* RNAi flies and evaluated their effect on locomotion activity and ATP production to confirm their therapeutic effects (Yang et al., 2006). The inactivation of PINK1 in *Drosophila* causes severe defects in mitochondrial morphology, muscular function, and dopaminergic neurons (Yang et al., 2006). The loss of Parkin in *Drosophila* produces very similar phenotypes with the inactivation of PINK1 (Greene et al., 2003, Shiba-Fukushima et al., 2017). We used *Drosophila* larvae expressing *PINK1* RNAi in muscles as a mitochondria-associated PD animal model for the following reasons: first, the reduced but not complete loss of PINK1-Parkin pathway activity would mimic most of the pathological conditions of PD caused by mitophagy defects, including those of PARK2 and PARK6 cases harboring milder mutations; secondly, controlling the amount of drug administered to *Drosophila* larvae is easy due to their stable feeding behavior; thirdly, their muscular mitochondrial phenotype is detected at an early stage and appears to be more severe than that of dopaminergic neurons. On the other hand, there are some limitations to the use of *Drosophila* as human disease models. It is difficult to evaluate the effects of compounds in aged animals using PD model flies, which show early developmental phenotypes, such as *PINK1* RNAi flies, because flies do not consume any food or water for 3.5–4.5 days during pupation, making it difficult to administer compounds constantly. Evaluation of the compounds for the survival and function of dopaminergic neurons in our fly models would be a challenge for further study. Nevertheless, *Drosophila* larval models have advantages in evaluating compounds targeting mitochondria in early screenings for drug repositioning at least as a mitochondria-associated PD model animal. The *Drosophila* genome contains at least ∼75% of ortholog genes for human diseases, and most cell-signaling pathways in mammals are conserved as simple frameworks (Reiter et al., 2001)**.** Moreover, mitochondrial degeneration caused by the defects in PINK1-Parkin signaling is more obvious than in rodent models (Clark et al., 2006, Kitada et al., 2007, Park et al., 2006, Yang et al., 2006). Thus, drug evaluation using *Drosophila* models is less expensive and can bypass ethical issues to assess approved or potential compounds in terms of highly conserved mitochondrial functions.

**Table 1:** Summary of the Therapeutic Effect of Candidate Drugs in Various PD Models Used in This Study

	PARK2A			PARK2B			PARK6			Idiopathic PD		*Drosophila*		
	Apoptosis (Basal)	Apoptosis (+CCCP)	Mito phagy	Apoptosis (Basal)	Apoptosis (+CCCP)	Mito phagy	Apoptosis (Basal)	Apoptosis (+CCCP)	Mito phagy	Apoptosis (Basal)	Mito phagy	Move ment	ATP	Mitochondrial Reduction
MRS 1220	–	+	+	–	+	+	–	+	+	–	–	+	–	–
Tranylcypromine	–	–	+	+	+	–	+	–	+	–	+	–	–	NA
Flunarizine	–	+	+	+	+	+	+	–	+	+	+	–	–	NA
Bromocriptine	–	–	+	+	+	+	+	+	+	+	+	+	+	+

The majority of patients with PD are idiopathic cases with multiple pathological causes, such as impaired mitochondrial homeostasis, α-synuclein accumulation, autophagic dysfunction, endoplasmic reticulum stress, and immunological dysfunction, due to genetic and environmental factors (Kalia and Lang, 2015). In this study, we newly established iPSCs from four idiopathic PD patients, and two out of these showed increased apoptosis and impaired mitochondrial clearance (Figures 6C and 6D). Unexpectedly, both lines had *LRRK2*-p.G2385R (heterozygote), which has been reported as a risk variant for PD (Di Fonzo et al., 2006, Tan et al., 2007). Because G2385R was found in approximately 5% of the control in an Asian population, this variant is not a pathogenic mutation (Funayama et al., 2007). The association of this variant with mitochondrial function has not yet been reported. It is unclear whether our proposed system can detect unknown genetic mutations, or whether these mutations are not relevant to the increment of apoptosis and impairment of mitochondrial clearance; therefore, further investigation with more samples is required. Interestingly, the identified drug candidates were effective in only one of two cases showing the phenotypes but ineffective in the other, indicating that, although a certain number of idiopathic cases show mitochondrial clearance abnormalities, the drugs we identified in PARK2/6 have limited efficacies. In conclusion, we were still able to show that our high-throughput phenotype detection system with familial PD neurons could be beneficial for patients with idiopathic PD having abnormal phenotypes common to familial PD.

## Experimental Procedures

### Culture of Human iPSCs

The control human iPSC lines 201B7 (Takahashi et al., 2007) and WD39 (Imaizumi et al., 2012), PARK2 lines PA9 and PB20 (Imaizumi et al., 2012), and PARK6 line PKB4/6 (Shiba-Fukushima et al., 2017) were cultured on mitomycin C-treated SNL murine fibroblast feeder cells in iPSC medium as described previously (Takahashi et al., 2007). Details about the iPSC lines are given in Table S1. All experimental procedures involving human iPSCs were approved by the Juntendo University School of Medicine Ethics Committee (approval no. 2017032).

### Isolation of Human T Cells and Induction into iPSCs on a Small Scale

iPSCs were derived from four patients with idiopathic PD and three age-matched healthy controls (detailed information is given in Table S2). Sendai viral induction was performed on a small scale as reported previously (Fujimori et al., 2018, Matsumoto et al., 2016) with slight modifications. Protocols for the iPSC induction and characterization are detailed in the Supplemental Experimental Procedures.

### Neural Induction

The differentiation into midbrain dopaminergic neurons was induced as reported previously (Fujimori et al., 2017, Imaizumi et al., 2015) with slight modifications and summarized in Figure S1A. In brief, 2 days after seeding iPSCs (day 0), 3 μM SB431542 (Tocris Bioscience, Avonmouth, UK), 3 μM dorsomorphin (Sigma-Aldrich), and 3 μM CHIR99021 (ReproCELL, Yokohama, Japan) were added to the iPSC medium for 5 days (days 0–5), which was replaced daily for 5 days. This is described as the CTraS method. To form neurospheres, on day 5 iPSC colonies were detached from the feeder cell layers using the Dissociation solution (ReproCELL), and then dissociated into single cells using TrypLE Select (Life Technologies, Carlsbad, CA, USA) at 37°C for 5–7 min. The dissociated and filtered (40 μm) cells were cultured at a density of 1 × 10^4^ cells/mL in KBM Neural Stem Cell medium (Kohjin Bio) supplemented with B27 (Life Technologies), 20 ng/mL basic fibroblast growth factor (PeproTech, Rocky Hill, NJ, USA), 2 μM SB431542 (Tocris Bioscience), and 5 μM Y27632 (Wako, Osaka, Japan) in 4% O_2_ atmosphere. On day 8, 3 μM CHIR99021 and 2 μM purmorphamine (Millipore, Burlington, MA, USA) were added to the culture medium. For terminal differentiation, on day 22 the neurospheres were dissociated by TrypLE Select with same protocol as day 5 and plated onto a 96-well plate (Corning, Corning, NY, USA) with poly-L-ornithine (Sigma-Aldrich) and Fibronectin (Corning) at a density of 2 × 10^4^ cells/well. The cells were cultured in KBM Neural Stem Cell medium supplemented with B27, 20 ng/mL brain-derived neurotrophic factor (BioLegend, Sandiego, CA, USA), glial cell-derived neuotrophic factor (PeproTech), 200 μM ascorbic acid (Sigma-Aldrich), 0.5 mM dibutyryl-cAMP (Nakalai Tesque, Kyoto, Japan), 1 ng/mL TGF-β3 (BioLegend), and 10 μM DAPT (Sigma) for 10 or 17 days before analysis. CHIR99021 was added to the medium only after the dissociated cells were plated. Every 2 days, 60% of the medium was replaced with fresh medium. We used neurons cultured for 10 days (day 32; mitophagy assay and apoptosis assay induced by CCCP) or 17 days (day 39; apoptosis assay in static condition and cell population assay) and plated onto 96-well plates.

### High-Content Analysis

For the cell population, mitophagy, ROS, and apoptosis assays, neurons were fixed and then stained with the antibodies listed in Table S3. The stained neurons on 96-well plates were automatically imaged by the IN Cell Analyzer 2200 imaging system (GE Healthcare) and then automatically analyzed by the IN Cell Developer Toolbox v.1.9 (GE Healthcare). An overview of the analysis is shown in Figure S2 and detailed in the Supplemental Experimental Procedures.

### Compound Library

We used a commercial inhibitor library (Sigma; S990043-INH4∼7) consisting of 320 compounds.

### *Drosophila* Genetics and Larval Assays

*Drosophila* lines with the following genotypes were used: UAS-mitoGFP/+, MHC-Gal4, UAS-PINK1 RNAi/+ (PINK1 RNAi), UAS-mitoGFP/UAS-LacZ RNAi, and MHC-Gal4/+ (LacZ RNAi). Eggs were laid on grape juice agar plates. The larvae were transferred to yeast chunks (0.6 g/mL distilled water), including 0.05% DMSO with or without drugs and were raised from the first-instar to the third-instar stage. Wandering is a behavior in *Drosophila* larvae before metamorphosis. Wandering larvae at late third-instar stage were used for the crawling assay. Their crawling, mitochondrial morphology, and ATP production were analyzed as described in the Supplemental Experimental Procedures.

### Statistical Analysis

The data are presented as the mean ± standard error of the mean (SEM). Analysis was performed using the JMP v.13 software (SAS Institute, Cary, NC, USA). Comparisons between the groups were performed using Steel's test or Dunnett's test after one-way ANOVA and Wilcoxon rank sum test. The effect of the compound treatment was analyzed using Wilcoxon signed-rank test. p values less than 0.05 were considered statistically significant.

## Author Contributions

A.Y., K.I., and W.A. conceived and designed the experiments. A.Y., K.I., T.I., K.S.-F., Y.L., M.F., and Y.I. performed the experiments and analyzed the data. S.S., T.H., A.M., Y.O., A.O., and N.H. contributed to the acquisition of patient samples and data. A.Y., K.I., Y.I., and W.A. wrote and revised the manuscript. All authors have reviewed and approved the manuscript.

## Supplemental Information

Document S1. Supplemental Experimental Procedures, Figures S1–S6, and Tables S1–S4

Document S2. Article plus Supplemental Information
