# Supplementary material for: Identifying Therapeutic Agents for Amelioration of Mitochondrial Clearance Disorder in Neurons of Familial Parkinson Disease
Source: Stem Cell Reports. 2020 May 28;14(6):1060–75. doi: 10.1016/j.stemcr.2020.04.011 (PMC7355139; doi:10.1016/j.stemcr.2020.04.011)
Supplement: Document S1. Supplemental Experimental Procedures, Figures S1–S6, and Tables S1–S4 [file mmc1.pdf]

Stem Cell Reports, Volume *14*

Supplemental Information

Identifying Therapeutic Agents for Amelioration of Mitochondrial Clear-ance Disorder in Neurons of Familial Parkinson Disease

Akihiro Yamaguchi, Kei-ichi Ishikawa, Tsuyoshi Inoshita, Kahori Shiba-Fukushima, Shinji Saiki, Taku Hatano, Akio Mori, Yutaka Oji, Ayami Okuzumi, Yuanzhe Li, Manabu Funayama, Yuzuru Imai, Nobutaka Hattori, and Wado Akamatsu

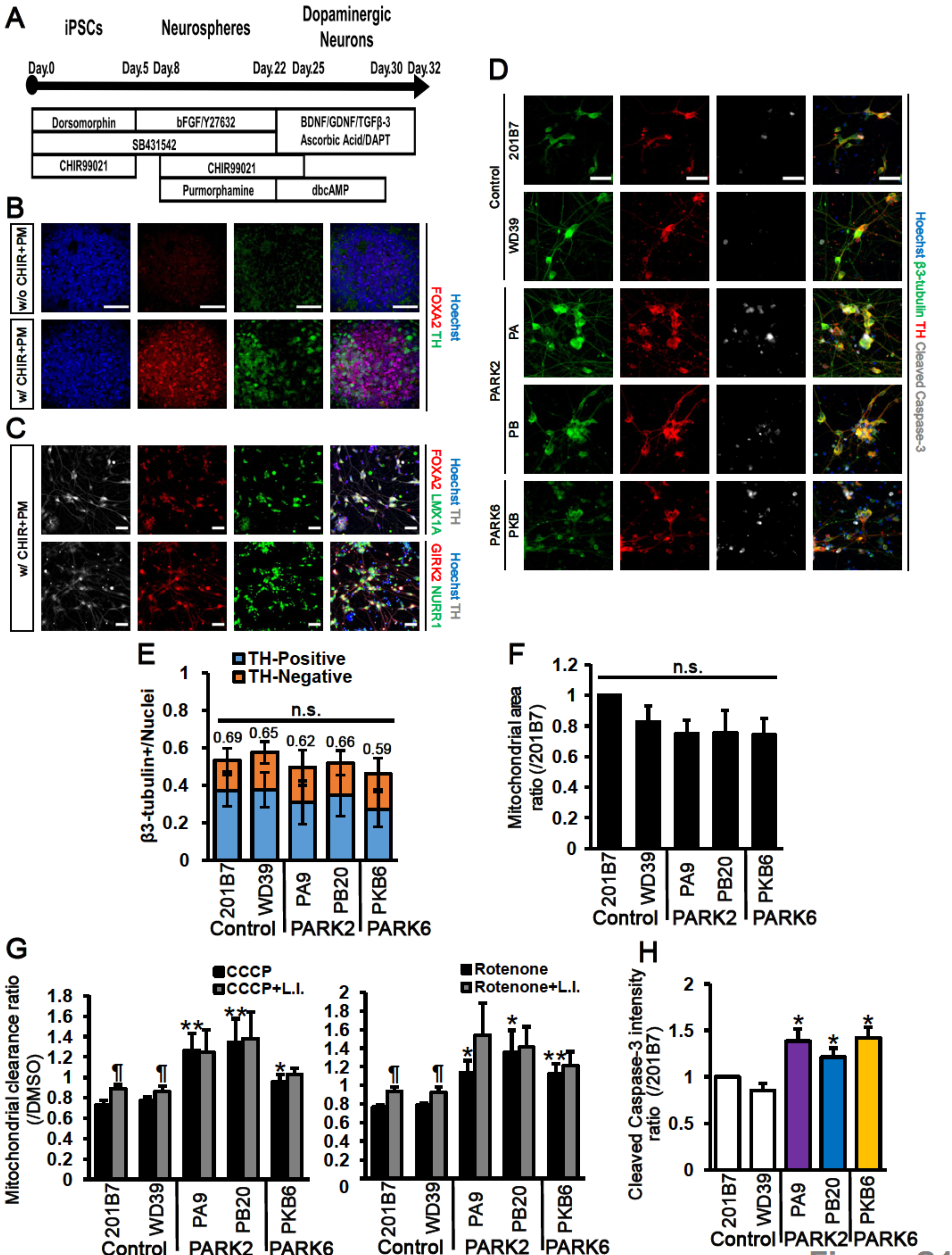
A
iPSCs
Neurospheres
Dopaminergic Neurons
Day.0 Day.5 Day.8 Day.22 Day.25 Day.30 Day.32
Dorsomorphin
bFGF/Y27632
BDNF/GDNF/TGFβ-3 Ascorbic Acid/DAPT
SB431542
CHIR99021
CHIR99021
Purmorphamine
dbcAMP
B
w/o CHIR+PM
w/ CHIR+PM
Hoechst FOXA2 TH
C
w/ CHIR+PM
Hoechst TH FOXA2 LMX1A
Hoechst TH GIRK2 NURR1
D
Control 201B7
WD39
PARK2 PA
PB
PARK6 PKB
Hoechst β3-tubulin TH Cleaved Caspase-3
E
TH-Positive
TH-Negative
β3-tubulin+/Nuclei
n.s.
0.69 0.65 0.62 0.66 0.59
201B7 WD39 PA9 PB20 PKB6
Control PARK2 PARK6
F
Mitochondrial area ratio (/201B7)
n.s.
201B7 WD39 PA9 PB20 PKB6
Control PARK2 PARK6
G
CCCP
CCCP+L.I.
Rotenone
Rotenone+L.I.
Mitochondrial clearance ratio (/DMSO)
201B7 WD39 PA9 PB20 PKB6
Control PARK2 PARK6
H
Cleaved Caspase-3 intensity ratio (/201B7)
201B7 WD39 PA9 PB20 PKB6
Control PARK2 PARK6

Figure S1

Figure S1. Overview and Characterization of PARK2/6 Dopaminergic Neurons. Related to Figure 1
(A) Schematic of the iPSC induction into midbrain dopaminergic neurons. (B) Characterization of neurosphere treated without (w/o) or with (w/) CHIR 99021 (CHIR) and Purmorphamine (PM) from 201B7. Immunocytochemical staining was performed with antibodies against a dopaminergic neuron marker (TH) and a midbrain marker (FOXA2). Scale bar = 50 μm. (C) Characterization of differentiated neuron treated with CHIR and PM from 201B7. Immunocytochemical staining was performed with antibodies against a dopaminergic neuron marker (TH) and midbrain markers (FOXA2, LMX1A, GIRK2, and NURR1). Scale bar = 50 μm. (D) Representative images of iPSC-derived dopaminergic neurons. Scale bar = 50 μm. (E) Quantitative analysis of β3-tubulin positive ratio. The percentage above the bar indicates TH-positive ratio in β3-tubulin-positive neurons (n=4 independent replicates; mean ± SEM). n.s. means not significant by one-way ANOVA. (F) Quantitative data of basal mitochondrial area, representing the ratio of mitochondrial area of neurons as standardized by 201B7 (n=7-9 independent replicates; mean ± SEM). n.s. means not significant by one-way ANOVA. (G) Validation of the mitochondrial uncoupler and lysosome inhibitor for mitochondrial clearance assay. Data represent the ratio of mitochondrial area of neurons treated with CCCP or rotenone with/without lysosomal inhibitors (L.I.; E64d and pepstatin A; n=9 independent replicates; mean ± SEM). *$p<0.05$; *$p<0.01$ compared with 201B7, ¶$p<0.05$ comparing CCCP treatment with CCCP+L.I. treatment by Wilcoxon rank-sum test. (H) Quantitative data of spontaneous apoptosis, representing the ratio of the intensity of cleaved caspase-3 in neurons and that of control (201B7) neurons (n=4-5 independent replicates; mean ± SEM). *$p<0.05$ compared with control (201B7) by Wilcoxon rank-sum test. CCCP means carbonyl cyanide 3-chlorophenylhydrazone.

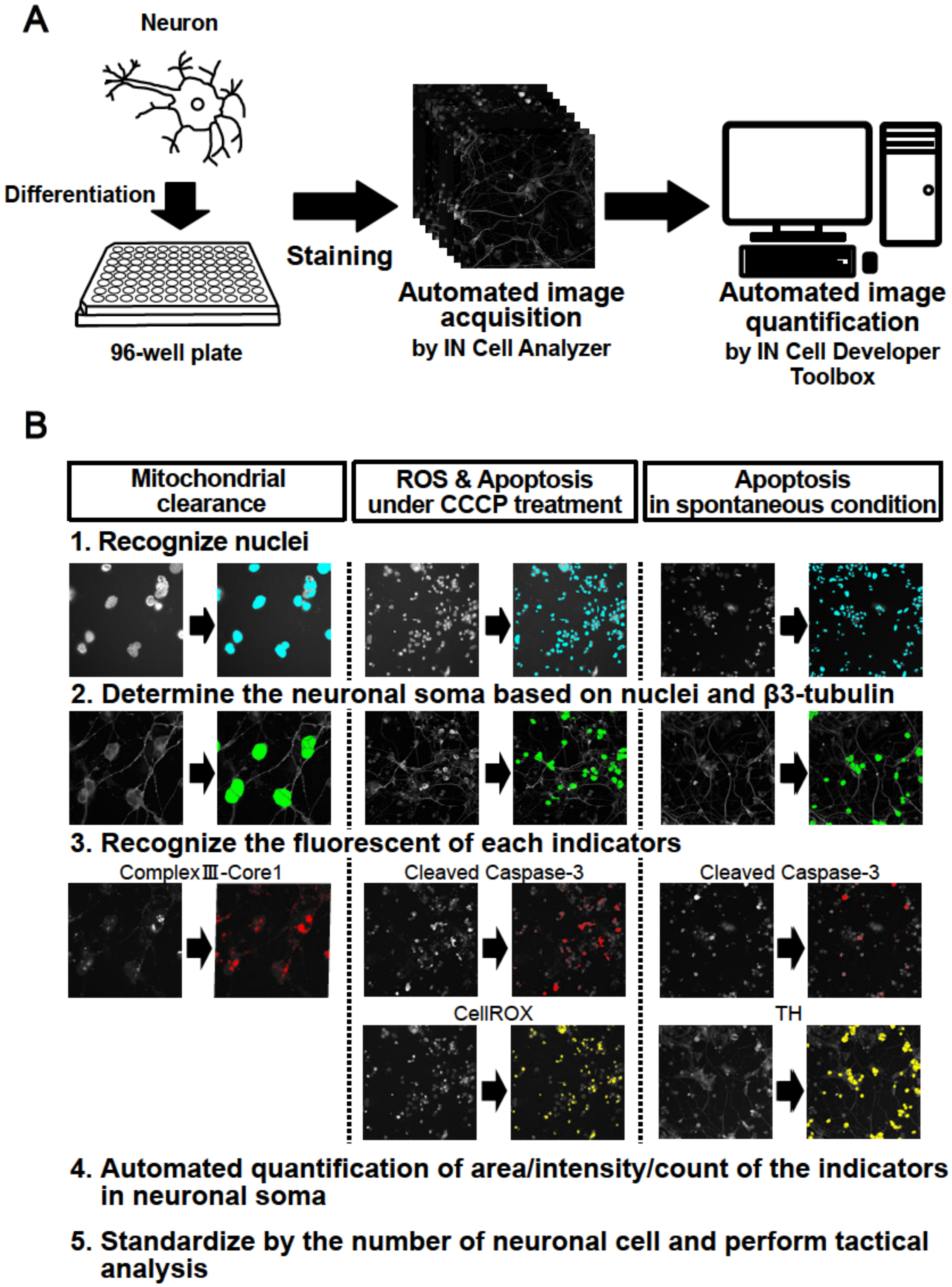
A
Neuron
Differentiation
96-well plate
Staining
Automated image acquisition
by IN Cell Analyzer
Automated image quantification
by IN Cell Developer Toolbox
B
Mitochondrial clearance
ROS & Apoptosis under CCCP treatment
Apoptosis in spontaneous condition
1. Recognize nuclei
2. Determine the neuronal soma based on nuclei and β3-tubulin
3. Recognize the fluorescent of each indicators
ComplexⅢ-Core1
Cleaved Caspase-3
Cleaved Caspase-3
CellROX
TH
4. Automated quantification of area/intensity/count of the indicators in neuronal soma
5. Standardize by the number of neuronal cell and perform tactical analysis

Figure S2

Figure S2. Schematic Representation of the Proposed Semi-Automated High-Throughput Phenotype Detection System. Related to Figure 2.

(A) Dopaminergic neurons were differentiated on 96-well plates and stained. All images were obtained automatically using the imaging cytometer and analyzed. (B) Recognition of cell features (e.g. nuclei, neural cell soma, mitochondria) and subsequent quantification of the mitochondrial area, CellROX® intensity, and cleaved caspase-3 intensity in the overlapped area were automatically analyzed. Results were standardized using the neuronal nuclei number in the field.

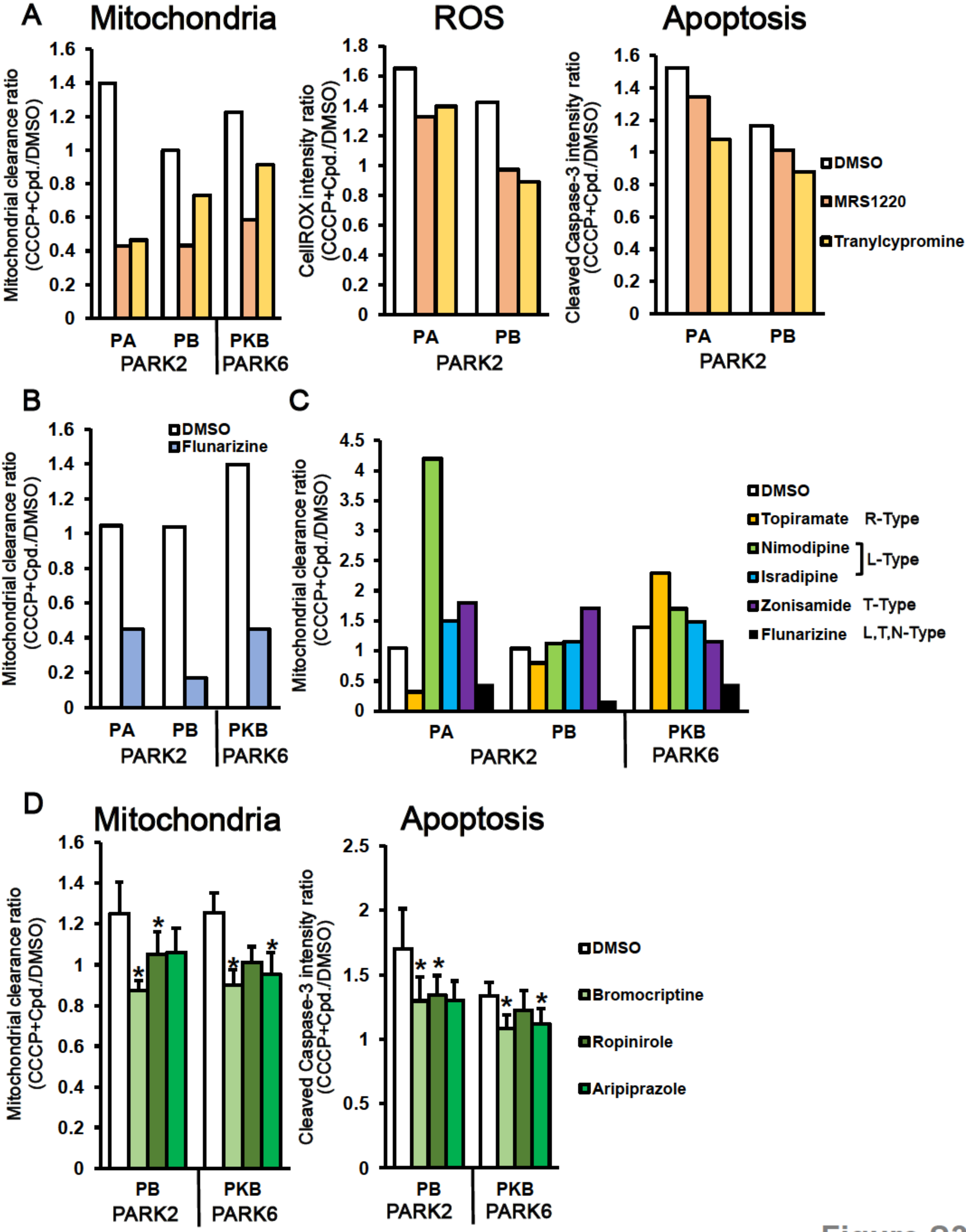
A
Mitochondria
Mitochondrial clearance ratio (CCCP+Cpd./DMSO)
PA
PB
PKB
PARK2
PARK6
ROS
CellROX intensity ratio (CCCP+Cpd./DMSO)
Apoptosis
Cleaved Caspase-3 intensity ratio (CCCP+Cpd./DMSO)
DMSO
MRS1220
Tranylcypromine
B
DMSO
Flunarizine
C
Topiramate R-Type
Nimodipine
Isradipine
L-Type
Zonisamide T-Type
Flunarizine L,T,N-Type
D
Bromocriptine
Ropinirole
Aripiprazole

Figure S3

Figure S3. Result of Each Compound in Screening and Validation of Calcium Channel Blockers and D_2 receptor Agonists. Related to Figure 2

(A) Results of MRS1220 and tranylcypromine in compound library screening. (B) Results of flunarizine in compound library screening. (C) Results of mitochondrial clearance assay using various types of calcium channel blockers. Screening assays were similarly performed as Figure 2A. (D) Mitochondrial clearance and apoptosis assays with several D_2 receptor agonists. For the mitochondrial clearance assay, data represent the ratio of the mitochondrial area in neurons treated with CCCP and each compound and that in neurons treated with DMSO (n=9 independent replicates; mean ± SEM). For the apoptosis assay, data represent the ratio of the fluorescence intensity of the cleaved caspase-3 treated with CCCP+compound and that treated with DMSO (n=9 independent replicates; mean ± SEM). *$p<0.05$ compared with CCCP+DMSO treatment using Wilcoxon signed-rank test. CCCP means carbonyl cyanide 3-chlorophenylhydrazone.

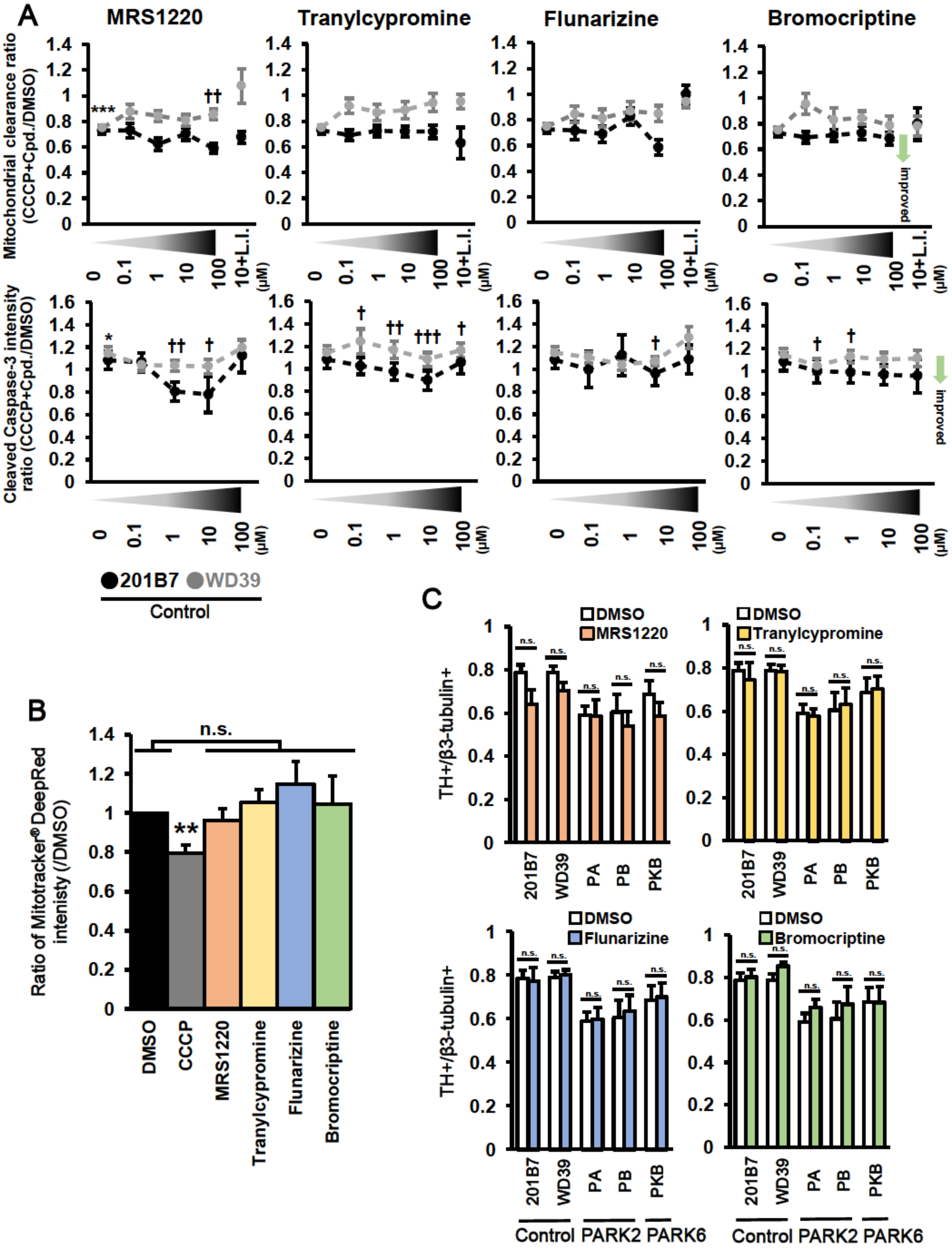
A
MRS1220
Tranylcypromine
Flunarizine
Bromocriptine
Mitochondrial clearance ratio (CCCP+Cpd./DMSO)
***
††
improved
0 0.1 1 10 100 10+L.I. (μM)
Cleaved Caspase-3 intensity ratio (CCCP+Cpd./DMSO)
*
†
††
†††
0 0.1 1 10 100 (μM)
201B7 WD39
Control
B
n.s.
**
Ratio of Mitotracker® DeepRed intenisty (/DMSO)
DMSO
CCCP
MRS1220
Tranylcypromine
Flunarizine
Bromocriptine
C
DMSO
MRS1220
Tranylcypromine
Flunarizine
Bromocriptine
n.s.
TH+/β3-tubulin+
201B7
WD39
PA
PB
PKB
Control PARK2 PARK6

Figure S4

Figure S4. Detailed Evaluation of Candidate Compounds. Related to Figure 3
(A) Quantitative data of candidate compounds in mitochondrial clearance and apoptosis assays in two control (201B7 and WD39) neurons. Plots show the results of DMSO, 0.1-100 μM of each candidate, and 100 μM of each candidate with lysosomal inhibitors (L.I.) under CCCP treatment. Data represent the mean ± SEM. (n=10 independent replicates). *$p<0.05$; ***$p<0.001$ compared with DMSO, †$p<0.05$; ††$p<0.01$; †††$p<0.001$ compared with CCCP+DMSO by Wilcoxon signed-rank test. L.I., lysosomal inhibitors (E64d and pepstatin A). (B) Effect of candidates on mitochondrial membrane potential. Mitotracker® signal becomes weaker when mitochondrial membrane potential decreased. Data represent the ratio of fluorescence intensity of Mitotracker DeepRed® in neurons treated with DMSO and that in neurons treated with the compound (n=9 independent replicates; mean ± SEM). **$p<0.01$ compared with DMSO by Wilcoxon signed-rank test. n.s. means not significant. (C) Effect of candidates on differentiation (TH-positive ratio). Data represent the mean ± SEM. (n=9 independent replicates). n.s. means not significant by one-way ANOVA. CCCP means carbonyl cyanide 3-chlorophenylhydrazone.

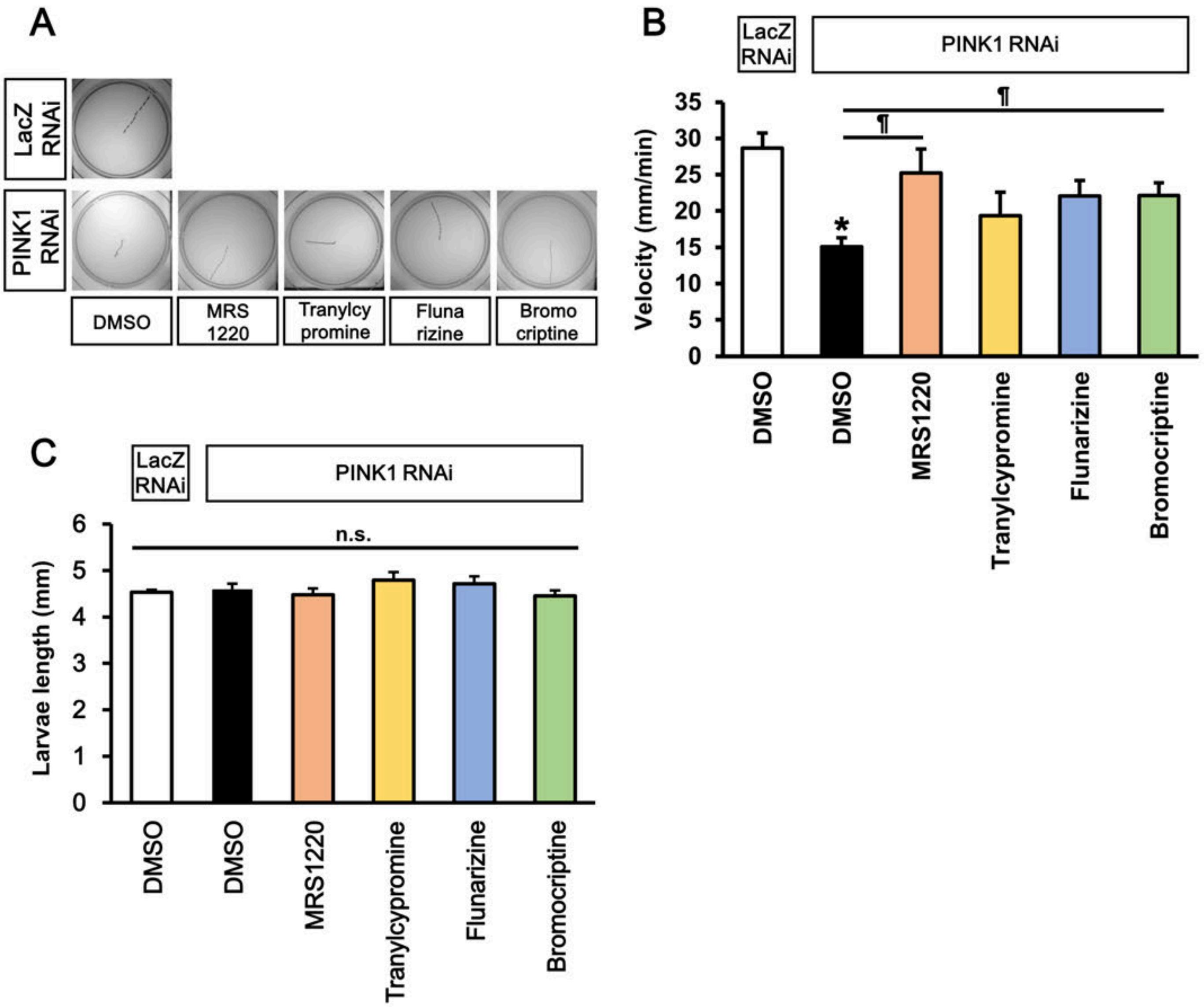
A
LacZ RNAi
PINK1 RNAi
DMSO
MRS 1220
Tranylcy promine
Fluna rizine
Bromo criptine
B
LacZ RNAi
PINK1 RNAi
Velocity (mm/min)
0
5
10
15
20
25
30
35
¶
¶
*
DMSO
DMSO
MRS1220
Tranylcypromine
Flunarizine
Bromocriptine
C
LacZ RNAi
PINK1 RNAi
Larvae length (mm)
0
1
2
3
4
5
6
n.s.
DMSO
DMSO
MRS1220
Tranylcypromine
Flunarizine
Bromocriptine

Figure S5

Figure S5. Candidate evaluation in *PINK1*-inactivated *Drosophila*. Related to Figure 5
(A) Tracking of representative larval movements treated with or without drugs. Larvae were placed at the center of dishes, and their movements recorded for 1 min. LacZ RNAi flies served as the healthy control. (B) Velocity of the movements measured in (A). Data represent the mean ± SEM (n=7-22 independent replicates). *$p<0.05$ compared with DMSO in LacZ RNAi, ¶$p<0.05$ compared with DMSO in PINK1 RNAi by Dunnett's test. (C) The body size of larvae with or without drugs. (n=7-24 independent replicates; mean ± SEM). n.s. means not significant by one-way ANOVA.

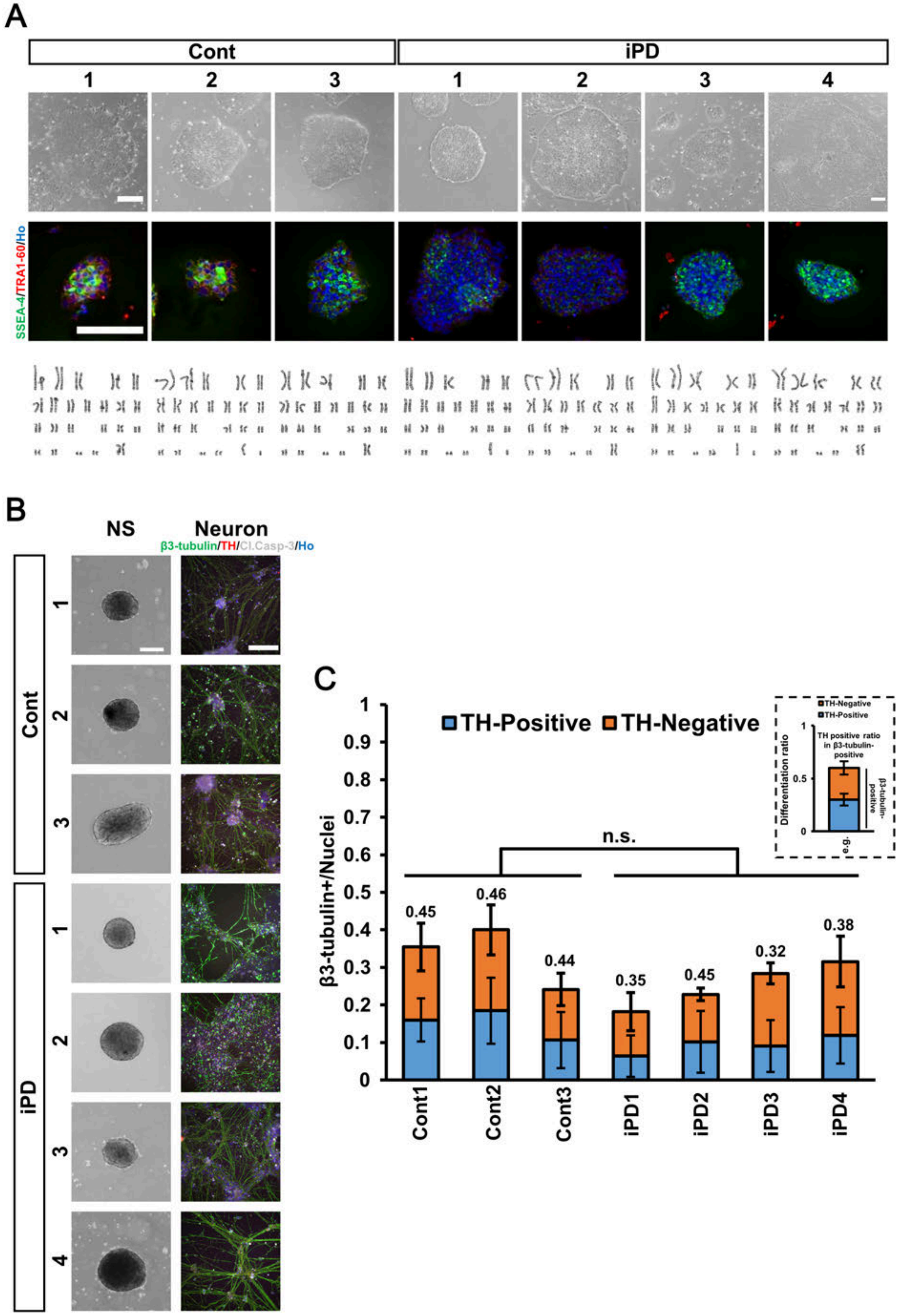
A
Cont
iPD
1
2
3
1
2
3
4
SSEA-4/TRA1-60/Ho
B
NS
Neuron
β3-tubulin/TH/Cl.Casp-3/Ho
Cont
iPD
C
TH-Positive
TH-Negative
n.s.
β3-tubulin+/Nuclei
0.45
0.46
0.44
0.35
0.45
0.32
0.38
Cont1
Cont2
Cont3
iPD1
iPD2
iPD3
iPD4
TH-Negative
TH-Positive
TH positive ratio in β3-tubulin-positive
Differentiation ratio
β3-tubulin-positive
e.g.

Figure S6

Figure S6. Characterization of Newly Established iPSCs derived from Control and Patients with Sporadic PD. Related to Figure 6

(A) Generation of iPSCs from patients with idiopathic PD and healthy controls. Representative images of iPSC colonies and their karyotypes are shown. Immunostaining of iPSCs was performed with multi-potency markers (SSEA-4 and TRA-1-60) and Hoechst. Scale bar = 200 µm. (B) Representative images of neurospheres (NS) and neurons. Immunostaining of iPSCs and neurons was performed with a neuronal marker (β3-tubulin), a dopaminergic neuron marker (TH), and Hoechst. Scale bar = 200 µm. (C) Quantitative analysis of β3-tubulin positive ratio. The percentage above the bar indicates the TH-positive ratio in β3-tubulin positive neurons (n=6-9 independent replicates; mean ± SEM). n.s. means not significant by one-way ANOVA.

Table S1. iPSCs derived from healthy donors and patients with familial PDs. Related to Figure 1.

Category	Gender	Ethnicity	Cells of Origin	Reprogramming Method	Sampling Age	Age of Onset	Risk Variants	H&Y Stage	Reference
Normal (201B7)	F	Caucasian	Dermal fibroblast	Retro virus	36	N/A	N/A	N/A	Takahashi et al. 2007
Normal (WD39)	F	Asian	Dermal fibroblast	Retro virus	16	N/A	N/A	N/A	Imaizumi et al. 2012
PARK2 (PA9)	F	Asian	Dermal fibroblast	Retro virus	72	62	*Parkin* Ex. 2-4 deletion homo	II	Imaizumi et al. 2012
PARK2 (PB20)	M	Asian	Dermal fibroblast	Retro virus	50	28	*Parkin* Ex. 6,7 deletion homo	N/A	Imaizumi et al. 2012
PARK6 (PKB4,6)	F	Asian	Dermal fibroblast	Retro virus	61	54	*PINK1* c.1162T>C homo	III	Shiba et al. 2017

Abbreviations: N/A, not available; H&Y Stage, Hoehn and Yahr stage.

Table S2. iPSCs derived from healthy donors and patients with idiopathic PDs. Related to Figure 6.

Category	Gender	Ethnicity	Cells of Origin	Reprogramming Method	Sampling Age	Age of Onset	Risk Variants	H&Y Stage
Cont1	F	Asian	PBC	Sendai virus	84	N/A	N/A	N/A
Cont2	M	Asian	PBC	Sendai virus	67	N/A	N/A	N/A
Cont3	F	Asian	PBC	Sendai virus	47	N/A	N/A	N/A
iPD1	M	Asian	PBC	Sendai virus	76	75	*LRRK2* p.G2385R hetero	III
iPD2	F	Asian	PBC	Sendai virus	66	60	N/A	I
iPD3	M	Asian	PBC	Sendai virus	66	56	*LRRK2* p.G2385R hetero	II
iPD4	F	Asian	PBC	Sendai virus	64	62	N/A	II

Abbreviations: PBC, peripheral blood cells; N/A, not available; H&Y Stage, Hoehn and Yahr stage.

Table S3. Antibodies used for staining. Related to Experimental Procedures.

Protein	Species	Source (catalogue #)	Dilution
FOXA2	Goat	Santa Cruz Biotechnology (sc-6554)	1:500
LMX1A	Rabbit	Sigma (HPA030088)	1:300
NURR1	Mouse	R&D (PP-N1404)	1:300
GIRK2	Rabbit	Alomone Labs (APC-006)	1:300
β3-tubulin	Mouse	Sigma (T8660)	1:1000
TH	Mouse	Sigma (T1299)	1:1000
TH	Rabbit	Millipore (AB152)	1:1000
Complex Ⅲ-Core1	Mouse	Sigma (459140)	1:300
TOM20	Rabbit	Santa Cruz Biotechnology (sc-11415)	1:300
Cleaved Caspase-3	Rabbit	Cell Signaling Technology (9661)	1:400
SSEA4	Mouse	Abcam (ab16287)	1:1000
TRA-1-60	Mouse	Millipore (MAB4360)	1:1000

Table S4. Compounds used for screening. Related to Experimental Procedures.

Compound	Molecular Weight	Source (catalogue #)	Concentration (reference)
DMSO	78.13	Fujifilm-Wako (048-21985)	0.3%
CCCP	204.62	Sigma (C2759)	30μM (Imaizumi et al. 2012.)
Bafilomycin A1	622.83	Sigma (B1793)	5μM (Imaizumi et al. 2012.)
E-64-d	342.43	Peptide Institute (4321-v)	30μM (Hirano et al. 2019.)
Pepstatin A	685.89	Peptide Institute (4397-v)	15μM (Hirano et al. 2019.)
Rotenone	394.42	Sigma (R8875)	10μM (Tabata et al. 2018.)
MRS 1220	403.83	Santa Cruz Biotechnology (sc-361259)	10μM
Tranylcypromine	169.65	Sigma (P8511)	10μM
Bromocriptine mesylate	750.70	Fujifilm-Wako (020-18471)	10μM
Flunarizine dihydrochloride	477.42	Sigma (F8257)	10μM
Ropinirole hydrochloride	296.84	Sigma (R2530)	10μM
Aripiprazole	448.39	Sigma (SML0935)	10μM

Supplemental Experimental Procedures

Immunocytochemistry

iPSCs, NS, or mDA neurons were fixed with 4% paraformaldehyde (PFA) in PBS at 23 °C for 30 min. The fixed mDA neurons in 96-well plates were washed with PBS automatically by a plate washer (HydroSpeed plate washer; Tecan) in the following steps. The cells were blocked with 5% FBS and 0.3% Triton X-100 in PBS for 30 min and then stained with the primary antibodies at 4 °C overnight (Table S3). After rinsing with PBS, the cells were incubated with species-specific Alexa Fluor 488-, Alexa Fluor 555-, or Alexa Fluor 647-conjugated secondary antibodies (1:500; ThermoFisher) and Hoechst 33258 (1:5000; Sigma) at 23 °C for 1 h (iPSC and mDA neurons) or overnight (NS). For whole mount image acquisition of NS, the stained samples were treated with a clearing solution of 60% glycerol and 2.5 M fructose in distilled H_2O (Dekkers et al., 2019) for 20 min. Then, the cleared samples were gently placed in microscope slides and mounted by the clearing solution. The images were obtained using a BZ-Z810 microscope (Keyence, Osaka, Japan) and IN Cell Analyzer 2200 imaging system (GE Healthcare, Chicago, IL, USA).

High-content analysis

For the cell population, mitophagy, ROS, and apoptosis assays, the stained neurons were imaged on 96-well plates by the IN Cell Analyzer 2200 imaging system (GE Healthcare). Twenty-five fields were automatically collected from each well using a 20× magnification for the cell population, ROS, and apoptosis assays, whereas 50 fields were collected from each well using a 60× objective for the mitophagy assay. The images were analyzed by IN Cell Developer Toolbox v1.9 (GE Healthcare). The nuclei, neuronal cell bodies, dopaminergic neurons, and mitochondria were identified with Hoechst, β3-tubulin, TH, and Complex III-Core 1 staining, respectively. Apoptosis was detected by cleaved caspase-3 staining. To analyze the phenotypes in neurons, the stained area of Complex III-Core 1 (mitochondrial clearance assay), and the fluorescence intensities of CellROX® (ROS assay) and cleaved Caspase-3 (apoptosis assay) in the neural soma recognized by Hoechst and β3-tubulin and/or TH signals were analyzed. The quantified area/intensity was normalized using the number of β3-tubulin and/or TH-positive nuclei. For the cell population assay, by setting the areas of β3-tubulin- and TH-positive cells, the numbers of Hoechst-positive total nuclei, β3-tubulin–positive nuclei, and TH-positive nuclei were analyzed. For drug screening, the raw value in each plate (Vr) was corrected (Vc) to account for variations between plates using the following formula:

$$Vc = Vr \times (\{the\ average\ value\ of\ CCCP\ treatment\ per\ DMSO\ in\ all\ plates\} \div \{the\ value\ of\ CCCP\ treatment\ per\ DMSO\ in\ the\ target\ plate\})$$

The overview of the analysis is shown in Figure S2.

Mitochondrial clearance assay

For induction of mitochondrial clearance, 30 µM CCCP (Sigma), 10 µM Rotenone (Sigma), or 0.3% DMSO were added in differentiation medium without dibutyryl-cAMP for 48 h. To assess the lysosomal degradation of mitochondria, 5 µM BafA1 (Sigma), or 30 µM E-64-d (Peptide Institute, Ibaraki, Japan) plus 15 µM pepstatin A (Peptide Institute) were also added at the same time. Subsequently, the cells were fixed and stained as described in "Immunocytochemistry", and automated analysis methods were discussed in "High-content analysis". The total mitochondrial area was normalized using the total β3-tubulin–positive nuclei number, and the mitochondrial clearance were evaluated using the ratio with DMSO treatment conditions.

Oxidative stress assay

After the CCCP treatment, the neurons were incubated with the CellROX® RGreen Reagent (Life Technologies) for 30 min at 37 °C following the manufacturer's instructions. Afterwards, the cells were fixed with 4% PFA at 23 °C for 30 min and stained with antibodies as described above. The automated analysis methods were as discussed in "High-content analysis." The total CellROX® intensity in neurons was normalized using the total β3-tubulin–positive nuclei number and calculated using the ratio of the CCCP treatment and DMSO treatment.

Mitochondrial membrane potential assay

After treatment with the compound for 2.5 h, the neurons were incubated with the Mitotracker® DeepRed FM (Life Technologies), a mitochondrial membrane potential indicator (Nakahira et al., 2011), at 37 °C for 30 min according to the manufacturer's instructions. Subsequently, the cells were fixed and stained. Image acquisition and quantification were performed by the IN Cell Analyzer 2200 imaging system and IN Cell Developer Toolbox v1.9, respectively (GE Healthcare).

Crawling assay of *Drosophila* larvae

The wandering larvae fed with or without compounds were placed in the middle of a 2% agar plate (100 mm in diameter). The body size of larvae in each group was not significantly different (Figure S5C). Larval positions were recorded every 7.5 s using a webcam (BSW20KM11BK; Buffalo, Nagoya, Japan) and traced by ImageJ software. The moving distance for 1 min was calculated using the ImageJ software.

Analysis of mitochondrial morphology of *Drosophila* larvae

Z-stacked images (5-µm thick) of mito-GFP–positive perinuclear mitochondria (40-μm^2-square containing a nucleus in the center) in the third-instar larval body wall muscle cells were obtained by confocal microscopy (Leica SP5). More than 2-μm^2-aggregated mitochondria were extracted by the 'Analyze particles' tool of the ImageJ software.

ATP assay

The effect of the compounds on ATP production was analyzed using CellTiter-Glo® Luminescent Cell Viability Assay (Promega, Madison, WI, USA). After treatment with compounds, 100 µL CellTiter-Glo® Reagent was added to the cells or *Drosophila* larval homogenates in white 96-well plates, and the samples were incubated according to the manufacturer's instructions. For the ATP measurement in *Drosophila* larvae, the effects of bacterial flora in the gut were estimated with and without the digestive organs. Because the effects of bacterial flora were negligible, whole bodies were homogenized in 40 µL of homogenization buffer (6 M guanidine-HCl, 100 mM Tris, and 4 mM EDTA; pH 7.8) and centrifuged at 16,000 g. The supernatants were diluted 1:500 and 1:10 with water and subjected to measurements of ATP and protein concentrations, respectively. ATP levels were estimated as luminescence intensity measured by a microplate reader (Mithras^2 LB943; Berthold, Bad Wildbad, Germany). The luminescence intensities were standardized with the amounts of proteins measured using the Pierce BCA Protein Assay Kit (Thermo Scientific).

Isolation of human T cells and small-scale induction into iPSCs

The peripheral blood mononuclear cells (PBMCs) were obtained from 4 patients with idiopathic PD and 3 age-matched healthy donors. (Detailed information is listed in Table S2.) After centrifugation, CD3- and CD28-positive cells were selected and expanded using Dynabeads® Human T-Activator CD3/CD28 (ThermoFisher Scientific, Waltham, MA, USA) in KBM 502 medium (Kohjin Bio, Sakado, Japan) for 5–7 days. For cryopreservation, we used CELLBANKER®2 (ZENOAQ, Koriyama, Japan), and thawing was performed according to the manufacturer's instructions. After thawing, the activated T cells were transferred to a 24-well plate with 500-µL KBM 502 medium at a density of 8 x 10^4 cells/well, and then, Cytotune®-iPS 2.0 Reprogramming Kit (MOI=5; DNAVEC, Tsukuba, Japan), 0.25 µM sodium butyrate (Sigma, St. Louis, MO, USA), and 5 µM cyclic pifithrin (Sigma) were added. At 24 h post-infection, 500 µL of the medium (KBM 502 + Sodium Butyrate, cyclic Pifithrin) was added. Defining the day on which the SeV infection was performed as day 0, the cells were collected on day 3 and transferred to a new 24-well plate coated with iMatrix 511-E8 (at a density of 0.5 µg/cm²; Nippi, Adachi-ku, Japan). On day 4, 1 mL of hiPSC medium (StemFit® AK02N; Ajinomoto, Chuo-ku, Japan) was added. After day 6, hiPSC medium was changed every other day until the colonies were visible. On day 10 until day 21, the cell-generated iPSC colonies were passaged to 6-well plates coated with iMatrix 511-E8 for subsequent maintenance and cryopreservation. We used cells at passage 5–10 for differentiation. G-band karyotyping of the established iPSCs were performed at LSI Medience Corporation (Japan).

Genetic analysis

PD-causative gene analysis was performed as previously reported (Shin et al., 2017). Briefly, PD- and dementia-associated genes, including *SNCA, Parkin, UCHL1, PINK1, DJ-1, LRRK2, ATP13A2,GIGYF2,*

HTRA2, PLA2G6, FBXO7, VPS35, EIF4G1, DNAJC6, SYNJ1, DNAJC13,CHCHD2, GCH1, NR3A2, VPS13C, RAB7L1, BST1, c19orf12, RAB39B, MAPT, PSEN1, GRN, APP, and *APOE*, were screened using high-throughput, next-generation sequencing such as Ion Torrent System (IAD103177_182; Thermo Fisher Scientific, Waltham, MA, US). The panel for sequencing was designed by Ion AmpliSeq Designer (https://www.ampliseq.com). Library preparation was performed by using an Ion AmpliSeq Kit for Chef DL8 (Thermo Fisher Scientific) and Ion Chef System (Thermo Fisher Scientific). Emulsion PCR was performed using an Ion 530 Kit-Chef, and the product was sequenced on an IonS5 Plus Sequencer with an Ion 530 Chip. Sequence alignment was performed using the Torrent Mapping Alignment Program aligner implemented in the Torrent Suite software (v5.10; Thermo Fisher Scientific).

Supplemental References

Dekkers, J.F., Alieva, M., Wellens, L.M., Ariese, H.C.R., Jamieson, P.R., Vonk, A.M., Amatngalim, G.D., Hu, H., Oost, K.C., Snippert, H.J.G., et al. (2019). High-resolution 3D imaging of fixed and cleared organoids. Nat. Protoc. *14*, 1756–1771.

Nakahira, K., Haspel, J.A., Rathinam, V.A.K., Lee, S.J., Dolinay, T., Lam, H.C., Englert, J.A., Rabinovitch, M., Cernadas, M., Kim, H.P., et al. (2011). Autophagy proteins regulate innate immune responses by inhibiting the release of mitochondrial DNA mediated by the NALP3 inflammasome. Nat. Immunol. *12*, 222–230.

Shin, S., Kim, Y., Oh, S.C., Yu, N., Lee, S.T., Choi, J.R., and Lee, K.A. (2017). Validation and optimization of the Ion Torrent S5 XL sequencer and Oncomine workflow for BRCA1 and BRCA2 genetic testing. Oncotarget *8*, 34858–34866.
