# Supplementary material for: Identifying Therapeutic Agents for Amelioration of Mitochondrial Clearance Disorder in Neurons of Familial Parkinson Disease
Source: Stem Cell Reports. 2020 May 28;14(6):1060–75. doi: 10.1016/j.stemcr.2020.04.011 (PMC7355139; doi:10.1016/j.stemcr.2020.04.011)
Supplement: Document S2. Article plus Supplemental Information [file mmc2.pdf]

Stem Cell Reports
Article
OPEN ACCESS

Identifying Therapeutic Agents for Amelioration of Mitochondrial Clearance Disorder in Neurons of Familial Parkinson Disease

Akihiro Yamaguchi,[1] Kei-ichi Ishikawa,[1,2,*] Tsuyoshi Inoshita,[3] Kahori Shiba-Fukushima,[3] Shinji Saiki,[2] Taku Hatano,[2] Akio Mori,[2] Yutaka Oji,[2] Ayami Okuzumi,[2] Yuanzhe Li,[2] Manabu Funayama,[1,2,4] Yuzuru Imai,[2,5] Nobutaka Hattori,[2,3,5] and Wado Akamatsu[1,*]

[1]Center for Genomic and Regenerative Medicine, Juntendo University Graduate School of Medicine, Tokyo 113-8431, Japan
[2]Department of Neurology, Juntendo University School of Medicine, Tokyo 113-8431, Japan
[3]Department of Treatment and Research in Multiple Sclerosis and Neuro-intractable Disease, Tokyo 113-8431, Japan
[4]Research Institute for Diseases of Old Age, Graduate School of Medicine, Juntendo University, Tokyo 113-8421, Japan
[5]Department of Research for Parkinson's Disease, Juntendo University Graduate School of Medicine, Tokyo 113-8431, Japan
*Correspondence: kishikaw@juntendo.ac.jp (K.-i.I.), awado@juntendo.ac.jp (W.A.)
https://doi.org/10.1016/j.stemcr.2020.04.011

SUMMARY

Parkinson disease (PD) is a neurodegenerative disorder caused by the progressive loss of midbrain dopaminergic neurons, and mitochondrial dysfunction is involved in its pathogenesis. This study aimed to establish an imaging-based, semi-automatic, high-throughput system for the quantitative detection of disease-specific phenotypes in dopaminergic neurons from induced pluripotent stem cells (iPSCs) derived from patients with familial PD having *Parkin* or *PINK1* mutations, which exhibit abnormal mitochondrial homeostasis. The proposed system recapitulates the deficiency of mitochondrial clearance, ROS accumulation, and increasing apoptosis in these familial PD-derived neurons. We screened 320 compounds for their ability to ameliorate multiple phenotypes and identified four candidate drugs. Some of these drugs improved the locomotion defects and reduced ATP production caused by PINK1 inactivation in *Drosophila* and were effective for idiopathic PD-derived neurons with impaired mitochondrial clearance. Our findings suggest that the proposed high-throughput system has potential for identifying effective drugs for familial and idiopathic PD.

INTRODUCTION

Parkinson disease (PD) is the second most common neurodegenerative disorder and is caused by the progressive loss of midbrain dopaminergic neurons (Kalia and Lang, 2015). Currently, its pharmacologic treatment is aimed primarily at correcting dopamine insufficiency. However, an effective disease-modifying therapy has yet to be established.

To date, more than 20 monogenic causative genes and numerous genetic risk factors have been identified (Deng et al., 2018). Familial cases with monogenic alterations comprise only a small percentage (of up to 10%) of PD patients, whereas the majority are idiopathic cases without any familial history (Tysnes and Storstein, 2017). Functional analyses of causative genes in familial PD suggest that various pathomechanisms, such as mitochondrial dysfunction, oxidative stress, α-synuclein accumulation, and impaired proteolysis, including macroautophagy and the ubiquitin proteasome pathway, underlie the dopaminergic neuronal loss in patients affected by PD (Kalia and Lang, 2015). Recent studies have suggested that mitochondrial dysfunction is a key factor in its pathophysiology. Mitochondrial dysfunctions have been reported in *Parkin* (PARK2), *PINK1* (PARK6)-, *DJ-1* (PARK7)-, *LRRK2* (PARK8)-, and *CHCHD2* (PARK22)-linked familial cases (Park et al., 2018), and several risk variants of these genes have been identified in idiopathic cases of PD (Kalia and Lang, 2015; Nalls et al., 2014). Moreover, the postmortem brain analysis of patients with idiopathic PD revealed mitochondrial dysfunctions, thereby suggesting their close association with the pathogenesis of idiopathic PD (Devi et al., 2008; Schapira et al., 1990; Sian et al., 1994). Therefore, identifying the therapeutic candidates for restoring impaired mitochondrial functions in PD could facilitate drug discovery for both familial and idiopathic PD.

PARK2 is the most common autosomal recessive (AR) form of early-onset PD (Lücking et al., 2000) caused by homozygous mutations in the *Parkin* gene. Mutations of the *PINK1* gene induce the second most frequent AR familial PD, named as PARK6. Clinicopathological phenotypes of these disorders in human and animal models are quite similar (Takanashi et al., 2016; Valente et al., 2004). In addition, it has been reported that Parkin is required for mitochondrial quality control, working closely with PINK1 protein kinase (Matsuda et al., 2010; Narendra et al., 2010). We and others have reported that the removal of damaged mitochondria in mitophagy is impaired in neurons derived from PARK2- and PARK6-induced pluripotent stem cells (iPSCs) after the accumulation of oxidative stress, thereby resulting in neuronal cell death (Imaizumi et al., 2012; Lahiri and Klionsky, 2017; Shiba-Fukushima et al., 2017).

In this study, we established an imaging-based, semi-automatic, high-throughput assay system for detecting both the cell viability and the impaired mitochondrial clearance in PARK2 (*Parkin*-Ex2-4 homozygous deletion

 © 2020 The Authors.
This is an open access article under the CC BY license (http://creativecommons.org/licenses/by/4.0/).

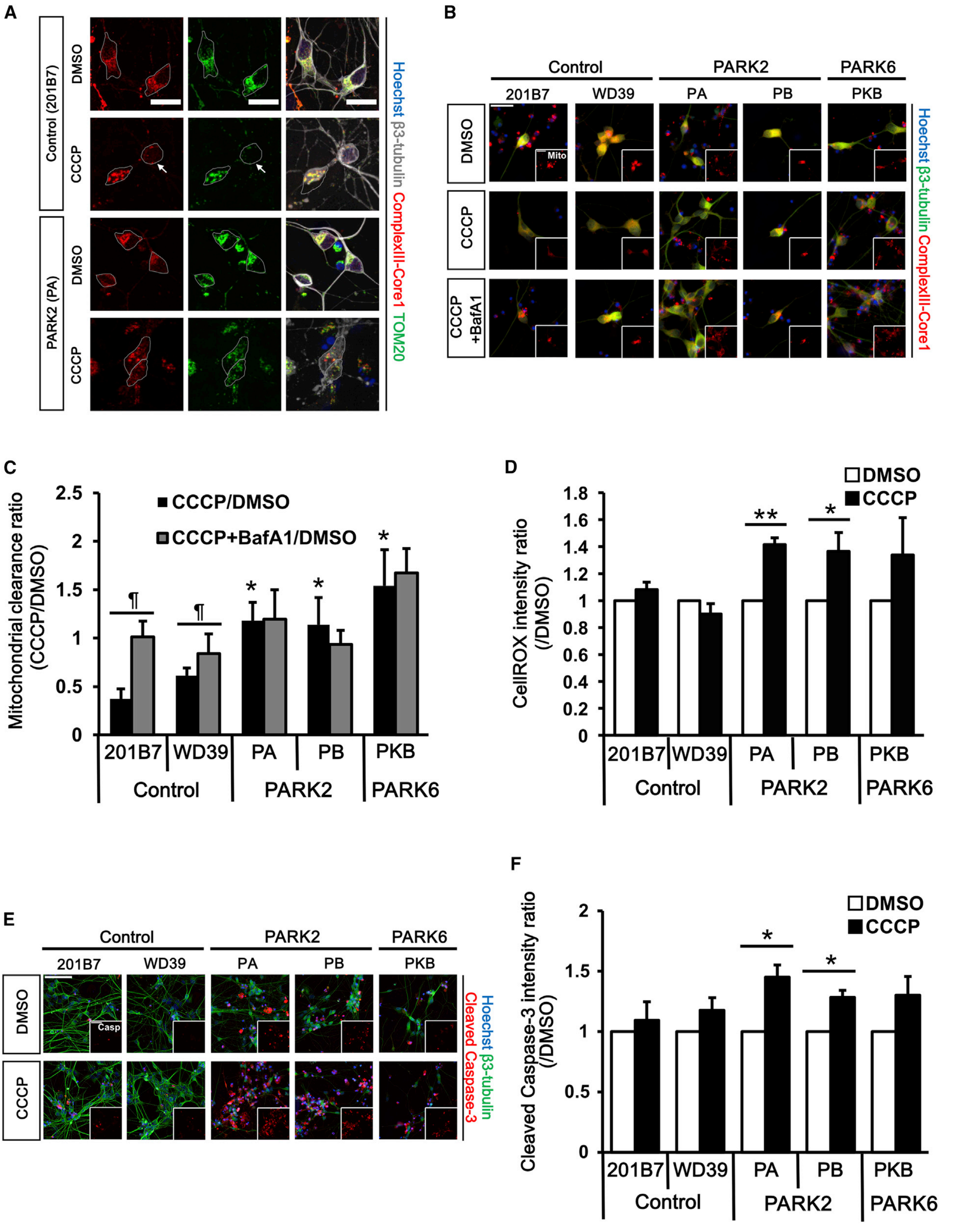

(legend on next page)

and *Parkin*-Ex6, 7 homozygous deletion) and PARK6 (*PINK1*-c.1162T>C homozygous mutation) patient-derived dopaminergic neurons, aiming to screen potential therapeutic drugs that improve mitochondrial dysfunction in PARK2/6 neurons. We used PARK2/6 iPSCs to screen 320 compounds and identified 4 that improved the pathogenetic phenotypes in dopaminergic neurons. We then verified the therapeutic effects of these drug candidates using a *Drosophila* PD model, as well as iPSCs derived from patients with idiopathic PD. The results suggest that our proposed high-throughput phenotype detection system for PARK2/6 neurons is an effective drug-screening platform for isolating therapeutic agents that can restore impaired mitochondrial clearance in PD.

RESULTS

High-Throughput Phenotype Detection of PARK2 and PARK6 iPSC-Derived Neurons

We have previously observed via immunofluorescent imaging that neurons differentiated from PARK2 and PARK6 iPSCs showed mitochondrial accumulation caused by impaired mitochondrial clearance (Imaizumi et al., 2012; Shiba-Fukushima et al., 2017). This phenotype is a fundamental pathomechanism of PD, including idiopathic cases. Therefore, we sought to increase the throughput of this method for applications to drug discovery and a large-scale cohort of PD-iPSC studies.

To establish an efficient analysis system for the monitoring of the PD-specific phenotypes of iPSC-derived neurons and for a large-scale drug screening, we first improved the method for neural differentiation. iPSCs were treated with SB431542 (transforming growth factor β3 [TGF-β] receptor inhibitor), dorsomorphin (AMPK inhibitor), and CHIR99021 (Wnt signal activator) for 5 days to induce embryoid body-like state (CTraS) cells to accelerate differentiation (Fujimori et al., 2017). These cells were then differentiated into neurospheres with region specificity of ventral midbrain by adding CHIR99021 and purmorphamine (Hedgehog signal activator) for 17 days as described previously (Imaizumi et al., 2015). Subsequently, the neurospheres were dissociated and plated onto 96-well plates for 10 days to induce neurons (Figure S1A). We confirmed that the neurospheres and the neurons differentiated with CHIR99021 and purmorphamine expressed midbrain markers (FOXA2, LMX1A, GIRK2, and NURR1) and a dopaminergic neuron marker (tyrosine hydroxylase [TH]) as seen in Figures S1B and S1C. Then, iPSC-derived dopaminergic neurons were treated by 30 μM carbonyl cyanide 3-chlorophenylhydrazone (CCCP), a mitochondrial membrane potential uncoupler, to induce mitochondrial elimination as described previously (Imaizumi et al., 2012) (Figure 1A).

To establish a drug-screening system using impaired mitochondrial clearance in PD iPSCs, we used two healthy controls, two PARK2 (*Parkin*-Ex2-4 homozygous deletion; PA and *Parkin*-Ex6, 7 homozygous deletion; PB), and one PARK6 (*PINK1*-c.1162T>C heterozygous mutation; PKB) iPSCs to derive dopaminergic neurons. After immunostaining with anti-β3-tubulin, complexIII-core1, cleaved caspase-3, and TH (Figure S1D), we confirmed that the differentiation efficiencies into dopaminergic neurons and the amounts of mitochondria at basal condition were not different among all the iPSCs clones (Figures S1E and S1F).

Figure S2 shows the schematic of the proposed high-throughput phenotype detection system. All images of neurons were obtained automatically using the imaging cytometer (IN Cell Analyzer 2200), while the subsequent recognition and quantification of the mitochondrial area in neurons were analyzed automatically by the imaging analyzer (IN Cell Developer Toolbox). Using this system, we detected that the mitochondrial area was unchanged in 30 μM CCCP-treated PARK2 and PARK6 neurons, but

Figure 1. Establishment of a High-Throughput Phenotype Detection System

(A) Immunostaining of control and PARK2 neurons with antibodies against mitochondrial proteins (ComplexIII-Core1 and TOM20) and a neuronal marker (β3-tubulin). Gray dotted lines indicate neuron cell bodies. Mitochondria are eliminated in the CCCP-treated control neuron (arrows). Scale bar, 20 μm.

(B) Representative images of the mitochondrial clearance assay. Scale bar, 20 μm.

(C) Quantitative data of the mitochondrial clearance assay. The mitochondrial area was reduced in day 32 control neurons treated with CCCP but not in day 32 PARK2 (PA and PB) and PARK6 (PKB) neurons. Data represent the ratio of mitochondrial area in neurons treated with CCCP (30 μM)/BafA1 (5 μM) and that in neurons treated with DMSO (n = 4 independent replicates; mean ± SEM). *$p < 0.05$ compared with 201B7, ¶$p < 0.05$ compared between CCCP treatment and CCCP + BafA1 treatment by Wilcoxon rank sum test.

(D) ROS accumulation assay. Data represent the ratio of fluorescent intensity of day 32 CCCP-treated neurons and that of DMSO-treated neurons (n = 5 independent replicates; mean ± SEM). *$p < 0.05$, **$p < 0.01$ compared with DMSO by Wilcoxon rank sum test.

(E) Representative images of the cell-viability assay. Scale bar, 100 μm.

(F) Quantitative data of the cell-viability assay. Data represent the ratio of fluorescence intensity of cleaved caspase-3 in day 32 CCCP-treated neurons and that in day 32 DMSO-treated neurons (n = 5 independent replicates; mean ± SEM). *$p < 0.05$ compared with DMSO by Wilcoxon rank sum test.

BafA1, bafilomycin A1; CCCP, carbonyl cyanide 3-chlorophenylhydrazone. See also Figure S1.

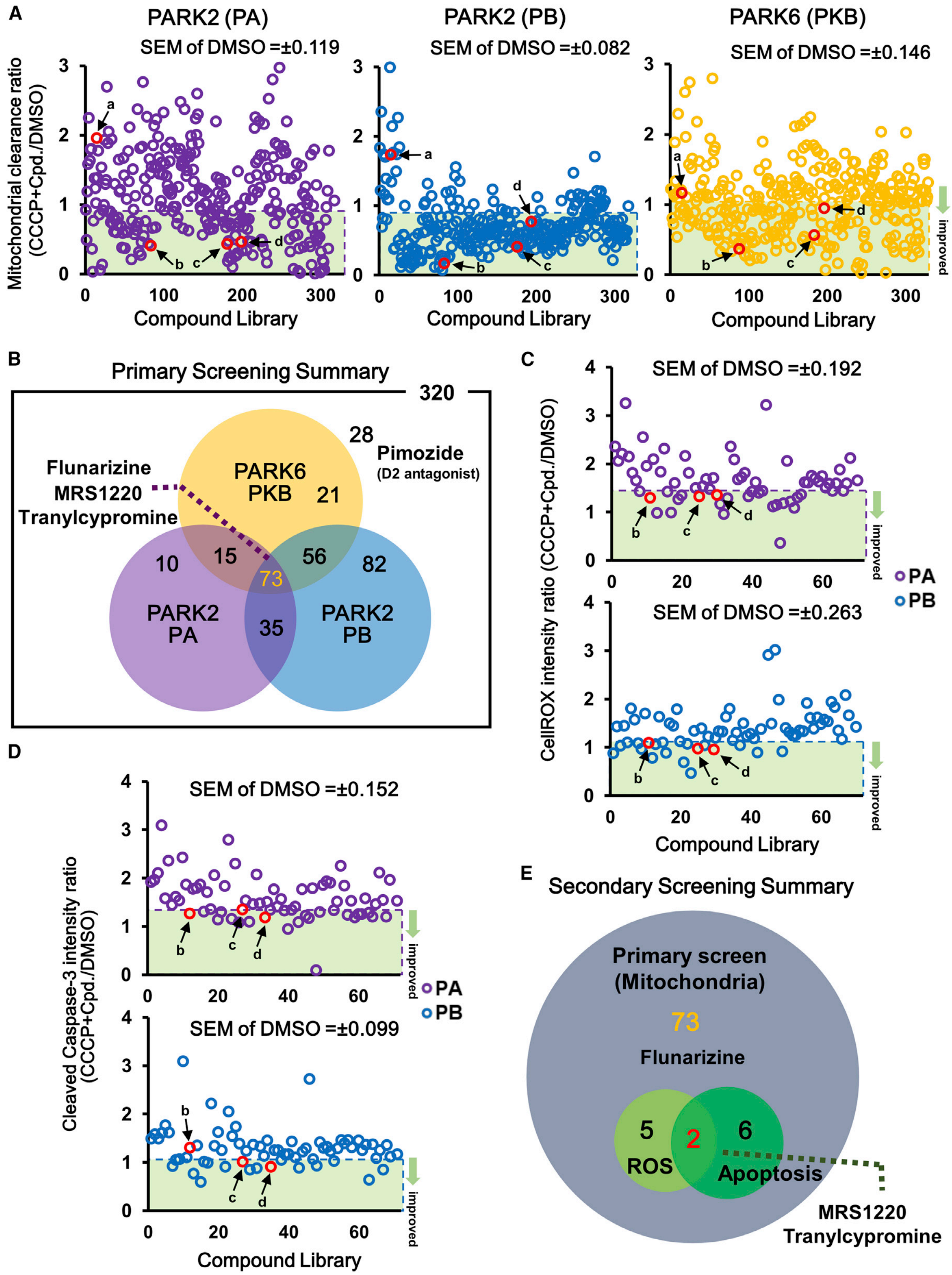

Figure 2. Screening for Compounds that Modify Mitochondrial Clearance in PARK2 and PARK6 Neurons

(A) A scatterplot of mitochondrial clearance screening for PARK2 and PARK6 neurons. Data represent the ratio of mitochondrial area in neurons treated with CCCP and compound (Cpd) and that in neurons treated with DMSO. Hit compounds (below the average value of CCCP +

(legend continued on next page)

was significantly decreased in CCCP-treated control neurons (Figures 1B and 1C), which is consistent with our previous observation (Imaizumi et al., 2012). We confirmed that the mitochondrial area reduction in CCCP-treated control neurons was attenuated by 5 μM bafilomycin A1 (BafA1, a V-ATPase inhibitor), confirming that the reduced area was caused by lysosomal degradation. (Figures 1B and 1C). Similar results were obtained in PARK2 and PARK6 neurons treated with a different mitochondrial uncoupler, rotenone, and/or the lysosomal inhibitors, E64d and pepstatin A (Figure S1G). These data suggest that the proposed imaging-based, high-throughput system is capable of detecting disease-specific phenotypes caused by impaired lysosomal degradation of damaged mitochondria in PARK2 and PARK6 neurons within a differentiation culture period of 32 days, which is a 1/3 times faster than conventional methods.

We then tested this system to detect other phenotypes of PARK2 and PARK6 neurons. By using CellROX, a reactive oxygen species (ROS) indicator, we evaluated the oxidative stress in PARK2 and PARK6 neurons. ROS accumulation due to mitochondrial dysfunction was observed in CCCP-treated PARK2 neurons (Figure 1D) as reported previously (Fujimori et al., 2017). Increased activation of caspase-3 in PARK2 neurons was also detected, suggesting that these neurons were sensitive to cell death (Figures 1E and 1F). Similarly, both ROS and cleaved caspase-3 signals tended to be higher in PARK6 neurons, but these differences were not statistically significant. These results suggest that our proposed method evaluated mitochondrial stress and apoptosis level of iPSC-derived neurons to some extent within 32 days.

Screening for Compounds that Improve Mitochondrial Clearance and Cell Viability in PARK2 and PARK6 iPSC-Derived Neurons

To verify the applicability of the proposed method to drug screening of PD, we screened 320 pharmacologically active inhibitor compounds for their ability to improve mitochondrial clearance and cell viability in PARK2 and PARK6 iPSC-derived neurons. For the primary screening, the mitochondrial area was evaluated in neurons differentiated from two PARK2-iPSC lines and one PARK6-iPSC line treated with CCCP and 10 μM inhibitor compounds. The candidate drugs were evaluated based on the improvement in mitochondrial clearance by calculating the ratio of the mitochondrial area treated with CCCP and that treated with DMSO or with each compound (Figure 2A). The hit criterion of the screening was defined as less than 1-fold of the absolute values of standard error in the mitochondrial clearance ratio of CCCP + DMSO-treated neurons. We identified 73 compounds that improved the clearance of mitochondria in all clones (Figure 2B). Next, we performed a secondary screening using two types of PARK2 neurons, i.e., PA and PB, in terms of reduced ROS accumulation and decreased cell death (Figures 2C and 2D) because those of PARK6 neurons did not show significant changes. The secondary screening identified two hit compounds, MRS1220, an A3 adenosine receptor (A3A-R) antagonist, and tranylcypromine, a Food and Drug Administration-approved monoamine oxidase (MAO) inhibitor used as an antidepressant, which decreased both ROS generation and apoptosis in PARK2 neurons (Figures 2E and S3A).

A recent study reported that L-type calcium-channel blockers (CCBs), namely benidipine and ML218, exert a neuroprotective effect against increased mitochondrial stress in PARK2/6 iPSC-derived dopaminergic neurons (Tabata et al., 2018). Therefore, we focused on five CCBs, i.e., topiramate, nimodipine, isradipine, zonisamide, and flunarizine, included in the library. As shown in Figures S3B and S3C, only flunarizine (L-, T-, and N-type CCB) was effective in improving mitochondrial clearance. Flunarizine was included in the 73 hit compounds from the primary screening but was excluded in the secondary screening because it was less effective in decreasing ROS levels and cell death. However, because flunarizine was highly effective in improving the mitochondrial clearance ratio in two PARK2 and one PARK6 neurons (Figure S3B), we decided to include it in our subsequent analyses.

DMSO treatment minus SEM) are indicated by the green band. Four notable compounds are indicated by the red frame. The average value ± SEM of CCCP + DMSO treatment: PA 1.06 ± 0.119, PB 1.00 ± 0.082, PKB 1.19 ± 0.146.

(B) Summary of the mitochondrial clearance screening (primary screening).

(C) A scatterplot of ROS accumulation screening for PARK2 neurons. Data represent the ratio of fluorescence intensity of CellROX in neurons treated with CCCP and compound and that in neurons treated with DMSO. Hit compounds (below the average value of CCCP + DMSO treatment minus SEM) are indicated by the green band. Three notable compounds are indicated by the red frame. The average value ± SEM of CCCP + DMSO treatment: PA 1.65 ± 0.192, PB 1.42 ± 0.263.

(D) A scatterplot of apoptosis screening for PARK2 neurons. Data represent the ratio of fluorescence intensity of cleaved caspase-3 treated with CCCP and compound to that treated with DMSO. Hit compounds (below the average value of CCCP + DMSO treatment minus SEM) are indicated by the green band. Three notable compounds are indicated by the red frame. The average value ± SEM of CCCP + DMSO treatment: PA 1.52 ± 0.152, PB 1.16 ± 0.099.

(E) Summary of ROS accumulation and apoptosis screening for PARK2 neurons (secondary screening).

CCCP, carbonyl cyanide 3-chlorophenylhydrazone; a, pimozide; b, flunarizine; c, MRS1220; d, tranylcypromine. See also Figure S3.

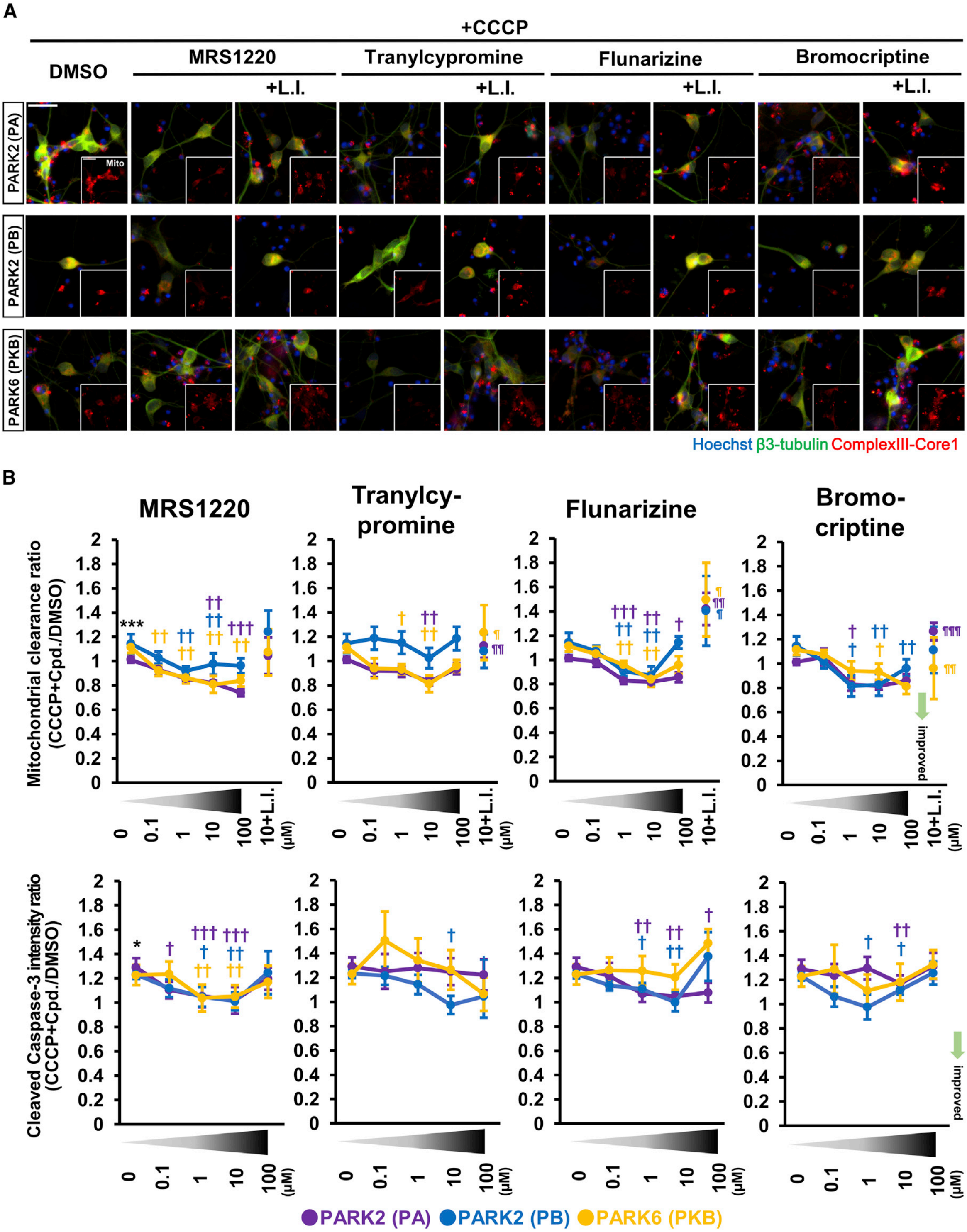

Figure 3. Candidate Compounds Show Reproducibility

(A) Images of the mitochondrial clearance analysis for the validation studies of 10 μM candidate compounds. Scale bar, 20 μm.

(B) Quantitative data of the candidate compounds in the mitochondrial clearance and apoptosis assays. Plots show the results of DMSO, 0.1–100 μM of each candidate, and 10 μM of candidate with lysosomal inhibitors under CCCP treatment. Data represent the mean ± SEM

(legend continued on next page)

Among the 320 compounds screened, 28 were found ineffective in terms of mitochondrial elimination in all 3 lines based on the primary screening (Figure 2B), including pimozide, a dopamine D_2 receptor antagonist. We further examined whether three D_2 receptor agonists, i.e., bromocriptine, ropinirole, and aripiprazol, were effective in improving mitochondrial clearance and suppressing cell death in PARK2 and PARK6 neurons. All three D_2 receptor agonists decreased the mitochondrial area and apoptosis with bromocriptine as the most effective (Figure S3D). Therefore, bromocriptine was included in subsequent analyses.

Hit Compounds and Their Effects on Mitochondrial Clearance and Apoptosis in Human PARK2 and PARK6 Neurons

To confirm the effect of the four hit compounds, i.e., MRS1220, tranylcypromine, flunarizine, and bromocriptine, on mitochondrial clearance and apoptosis in CCCP-treated neurons, we conducted repetitive tests with 0–100 μM of each compound. As shown in Figures 3 and S4A, all the hit compounds accelerated mitochondrial elimination in a partially dose-dependent manner in PARK2 and PARK6 neurons treated with CCCP. Furthermore, the decrease in the mitochondrial area caused by these compounds was attenuated by lysosomal inhibitors, E64d and pepstatin A (Figures 3A, 3B, and S4A). These results suggested that all four compounds promoted lysosomal degradation of the mitochondria. MRS1220, flunarizine, and bromocriptine exerted anti-apoptotic effects on neurons in a dose-dependent manner with concentrations ranging from 0.1 to 10 μM; however, they seemed to show toxicity at 100 μM. Tranylcypromine showed an anti-apoptotic effect at 100 μM, but its effect on mitochondrial clearance was not significant at this concentration.

To exclude the possibility that the enhanced mitochondrial elimination might be induced by additional mitochondrial damage caused by these four compounds, we examined the mitochondrial membrane potential in neurons treated with 10 μM of the compounds. None of the compounds reduced the mitochondrial membrane potential (Figure S4B), indicating that they were unlikely to induce additional mitochondrial damage. In addition, these compounds did not affect neural differentiation and maturation (Figure S4C). We concluded from these observations that the optimal concentration of all four compounds to be used in subsequent experiments is 10 μM.

We next investigated the anti-apoptotic effects of these compounds in PARK2 and PARK6 neurons under a CCCP-untreated static condition. Increased apoptosis in PARK2 and PARK6 neurons without CCCP treatment was not significant by day 32, as identified by the proposed detection system. However, with the addition of culture by day 39, a significant increase in cell death became detectable (Figure S1H). All the hit compounds, except MRS1220, significantly reduced the fluorescence intensity of the cleaved caspase-3 in PARK2 and PARK6 neurons compared with DMSO (Figures 4A and 4B). Although it was not statistically significant, the mean value of the fluorescence intensity of the cleaved caspase-3 was decreased by MRS1220. These results suggest that the hit compounds identified by our high-throughput phenotype detection system could significantly modify multiple phenotypes in PARK2 and PARK6 neurons, thus confirming the validity of the proposed screening method.

Therapeutic Effects of the Candidate Compounds in a *Drosophila* PD Model

Testing on animal models is an important step in drug discovery. However, mice harboring *PARK2* or *PARK6* mutations do not reproduce the degeneration of midbrain dopaminergic neurons (Goldberg et al., 2003; Itier et al., 2003; Kitada et al., 2007). Indeed, it was difficult to evaluate a PINK1 activation drug, kinetin triphosphate, in *PINK1* mutant mice (Orr et al., 2017). In contrast to rodent models, *PINK1*- or *Parkin*-deficient *Drosophila* exhibit prominent mitochondrial degeneration from an early adult stage (Clark et al., 2006; Park et al., 2006; Yang et al., 2006). Thus, we orally administrated the candidate compounds to *Drosophila* third-instar larvae expressing *PINK1 shRNA* in their muscular tissues (*PINK1*-KD). Although the loss of dopaminergic neurons was hardly detected in this larval stage of *PINK1*-deficient flies, *PINK1*-KD larvae showed apparent locomotion defects, suggesting that the *PINK1*-KD larvae partly reflect the prodromal stage of mitochondria-associated PD (Figures S5A–S5C). In contrast, the larvae expressing control LacZ RNAi (*LacZ*-KD) had normal movements (Figures S5A and S5B). MRS1220 and bromocriptine alleviated the movement disorder of *PINK1*-KD larvae. The other two compounds tended to improve the locomotion defects caused by *PINK1* inactivation (Figure S5B). To verify that the improvements were due to the recovery of mitochondrial functions, we quantified the amount of ATP in larval whole bodies. *PINK1*-KD larvae had lower ATP production than *LacZ*-KD larvae and, among the four candidate compounds, only bromocriptine stimulated the ATP production in *PINK1*-KD larvae (Figure 5A). Mitochondrial aggregation caused by *PINK1* inactivation was also alleviated

(n = 10 independent replicates). *p < 0.05, ***p < 0.001 compared with DMSO; †p < 0.05, ††p < 0.01 compared with CCCP + DMSO; ¶p < 0.05, ¶¶p < 0.01, ¶¶¶p < 0.001 compared with CCCP + 100 μM compound by Wilcoxon signed rank test. Data represent the mean ± SEM. CCCP, carbonyl cyanide 3-chlorophenylhydrazone; L.I., lysosomal inhibitors (E64d and pepstatin A). See also Figure S4.

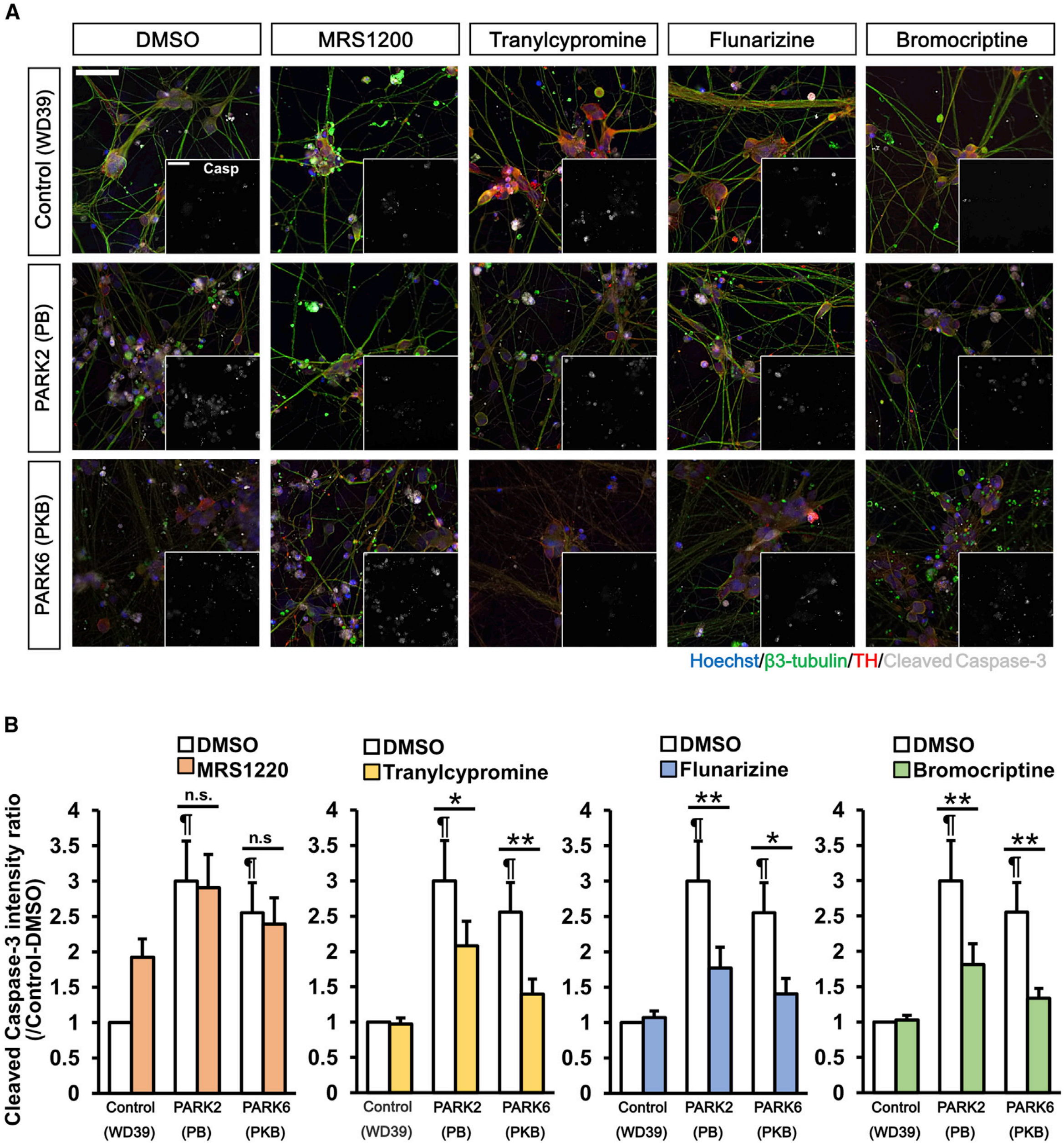

Figure 4. Tranylcypromine, Bromocriptine, and Flunarizine Reduce Spontaneous Apoptosis in PARK2 and PARK6 Neurons

(A) Images of day 39 neurons treated with 10 μM compounds for apoptosis analysis in static state. Scale bar, 100 μm.

(B) Quantitative data of the apoptosis analysis. Data represent the ratio of fluorescence intensity of cleaved caspase-3 in DMSO-treated control (WD39) neurons and that in 10 μM compound-treated neurons (n = 9 independent replicates; mean ± SEM). $^{*}p < 0.05$, $^{**}p < 0.01$ compared with DMSO; ¶$p < 0.05$ compared with DMSO-treated control (WD39) neuron by Wilcoxon signed rank test. n.s., not significant.

by bromocriptine but not by MRS1220 (Figure 5B). Moreover, bromocriptine did not affect larval locomotion in *LacZ*-KD larvae (Figure 5C). These results suggest that at least one candidate compound identified by our detection system, i.e., bromocriptine, exerts beneficial effects in *PINK1*-KD flies, thereby showing potential as therapeutic drug for PD.

Therapeutic Effects of the Candidate Compounds in iPSC-Derived Neurons from Patients with Idiopathic PD

Because idiopathic PD accounts for 90% of PD cases, we further evaluated the therapeutic effects of the four candidate compounds in idiopathic PD iPSC-derived neurons. We generated iPSCs from CD3-positive T lymphocytes

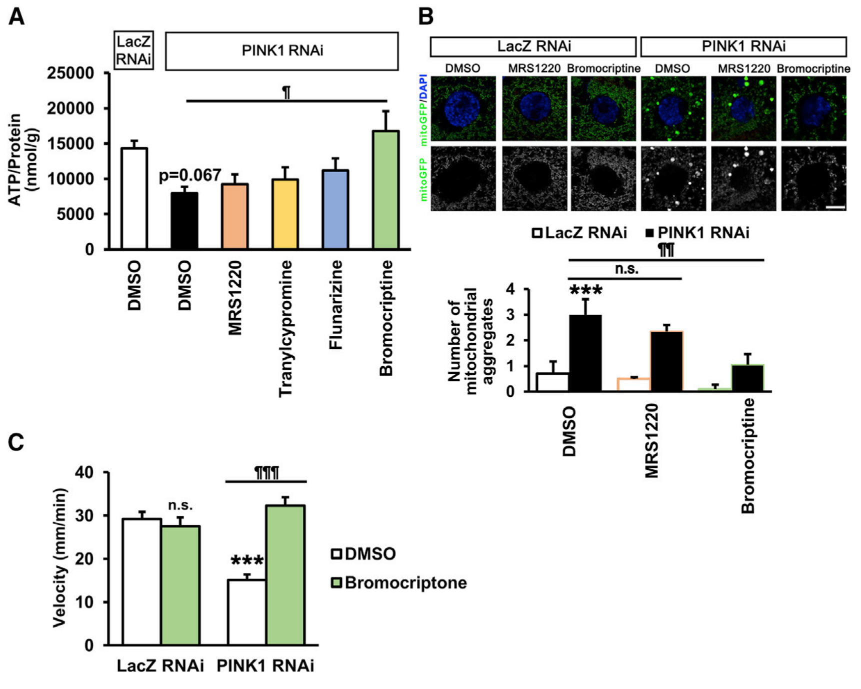

Figure 5. Bromocriptine Rescues Abnormal Phenotypes of *PINK1*-Inactivated *Drosophila*

(A) Whole-body ATP levels of *PINK1 RNAi* flies treated with or without drugs. Data represent the mean ± SEM (n = 9 independent replicates). ¶p < 0.05 compared with DMSO in *PINK1 RNAi* by Dunnett's test.

(B) Mitochondrial morphology of *PINK1 RNAi* flies treated with or without the indicated drugs. The number of greater than 2 μm^2 mitochondrial aggregates was graphed. Data represent the mean ± SEM (n = 20 cells from 5 independent flies). ***p < 0.001 compared with DMSO in *LacZ RNAi*, ¶¶p < 0.01 compared with DMSO in *PINK1 RNAi* by Tukey-Kramer test. n.s., not significant. Scale bar, 10 μm.

(C) Bromocriptine did not have any effect on the motor behavior of *LacZ RNAi* flies. Assay was performed as in Figure S5B. Data represent the mean ± SEM (n = 9–35 independent replicates). ***p < 0.001 compared with DMSO in *LacZ RNAi*, ¶¶¶p < 0.001 compared with DMSO in *PINK1 RNAi* by Tukey-Kramer test. n.s., not significant.

derived from four patients with idiopathic PD (iPD1-4) and three age-matched healthy controls (Cont1-3; Table S2). All established iPSC clones showed typical embryonic stem cell-like morphology and were positive for pluripotent markers (Figure S6A). We confirmed no difference in induction efficiency of dopaminergic neurons between the healthy controls and idiopathic PDs (Figures S6B and S6C). To elucidate the idiopathic PD phenotypes and evaluate the effect of the candidate compounds, we examined cell death and mitochondrial clearance abnormalities in neurons derived from each idiopathic iPSC clone as readouts of the appropriate phenotypes.

Quantitation of the cleaved caspase-3-positive neurons using the same protocol as that in familial PDs revealed that the two idiopathic PD lines, iPD1 and iPD3, significantly increased apoptosis compared with the healthy control line WD39 (Figure 6A). Interestingly, only these two lines showed impaired elimination of damaged mitochondria (Figures 6B and 6C). To eliminate the possibility that these iPSC lines have unknown genetic mutations, we analyzed all PD-related genes. Interestingly, two other idiopathic PD lines have LRRK2-p.G2385R (heterozygote), which has been reported as a PD-risk variant (Di Fonzo et al., 2006; Tan et al., 2007). These results suggest that some idiopathic cases recapitulate the phenotypic features of PARK2 and PARK6 neurons.

To evaluate the efficacy of the candidate compounds in idiopathic PD iPSCs, we treated the two idiopathic PD lines, i.e., iPD1 and iPD3, that exhibit impaired mitochondrial clearance and increased apoptosis with these compounds. Tranylcypromine, flunarizine, and bromocriptine significantly improved the impaired mitochondrial clearance in iPD1 line but not in iPD3 (Figure 6D). Flunarizine and bromocriptine also significantly decreased apoptosis in iPD1, but tranylcypromine did not (Figure 6E). These results suggest that bromocriptine is effective for a specific type of idiopathic PD. We conclude that the therapeutic drug candidates identified by our high-throughput phenotype detection system using PARK2/6 iPSCs are effective in other PD models, such as *Drosophila* and idiopathic PD with impaired mitochondrial clearance.

DISCUSSION

We have established an imaging-based, high-throughput phenotype detection system for neurons derived from PARK2 and PARK6 iPSCs and have assessed its applicability through compound library screening. There have been a few studies that performed drug screening with neurons from iPSCs in neurodegenerative disorders (Imamura et al., 2017; Kondo et al., 2017; Tabata et al., 2018). Because the efficacy of neutron differentiation is critical for the assays, in most studies the neurons were differentiated from iPSCs through the viral expression of neuron-generating genes or through long-term self-renewing neuroepithelial-like stem cells (lt-NES cells). In this study, we have analyzed a large number of iPSC-derived neurons

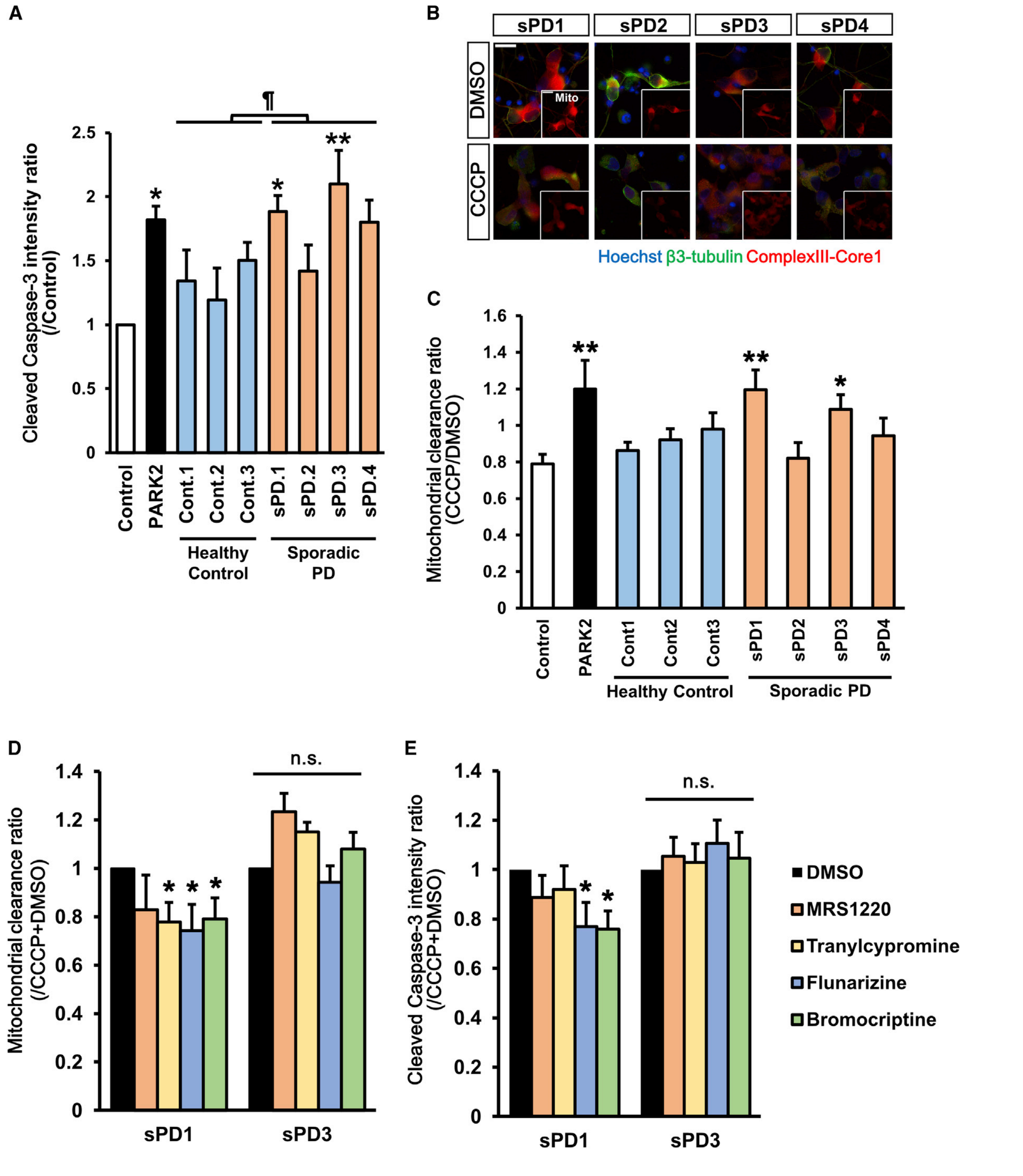

Figure 6. Evaluation of Drug Efficacy in Idiopathic PD-Derived Neurons

(A) Apoptosis assay in idiopathic PD-derived neurons. Data represent the ratio of the intensity of cleaved caspase-3 in day 39 idiopathic PD-derived neurons and that in control (201B7) neurons (n = 6–9 independent replicates; mean ± SEM). *$p < 0.05$ compared with control neurons by Wilcoxon rank sum test, ¶$p < 0.05$ compared between Control (Cont) and idiopathic PD (iPD) neurons by Student's t test.

(B) Images of mitochondrial clearance analysis in day 32 neurons. Scale bar, 20 μm.

(C) Quantitative data of mitochondrial clearance analysis. Data represent the ratio of mitochondrial area in neurons treated with CCCP and that in neurons treated with DMSO (n = 7–8 independent replicates; mean ± SEM). *$p < 0.05$, **$p < 0.01$ compared with control (201B7) by Wilcoxon rank sum test.

(legend continued on next page)

with sufficient induction efficiency in a short culture period by accelerating iPSC differentiation via embryo body-like state by treatment with three chemicals (Fujimori et al., 2017). Moreover, because our automatic analysis system is based on immunofluorescent imaging, we can select the neurons by markers. Because cell culturing and immunostaining are still performed manually, our proposal can be considered a "semi"-automatic high-throughput assay system. This system can recapitulate disease-specific phenotypes similar to previous reports in the same control and familial PD iPSC-derived neurons (Imaizumi et al., 2012; Shiba-Fukushima et al., 2017). Because we focused on the reproducibility of the phenotype detection, we built this system using the same iPSCs, but it might have been better to use other iPSCs as normal controls, such as age-matched or mutation-corrected iPSCs. However, this system managed to detect disease phenotypes in some idiopathic PD and control iPSCs that were not used for drug screening.

We investigated whether drugs identified using iPSC-derived neurons with abnormal mitochondrial clearance were also effective for the majority of patients with PD, including those with idiopathic cases and presumed similar mitochondrial abnormalities. We focused mainly on the phenotype of the mitochondrial clearance abnormality and identified four drugs, i.e., MRS1220, tranylcypromine, flunarizine, and bromocriptine. Mitochondrial quality control is regulated by both the removal of the damaged mitochondria and mitochondrial biogenesis (Pickles et al., 2018). The damaged mitochondria are mainly eliminated by mitophagy, but they can also be degraded by the other processes, such as macroautophagy and the ubiquitin proteasome system (Pickles et al., 2018; Yoshii et al., 2011). In this study, the involvement of lysosomes in the degradation of mitochondria was confirmed, but we did not examine in detail how mitochondria are degraded and how the candidate drugs promote the degradation of mitochondria. Both an A3A-R antagonist, which is reversine, and a histone lysine-specific demethylase 1 inhibitor, which is also a MAO inhibitor, activated autophagy via Akt/mTORC1 inhibition in several cell lines (Ambrosio et al., 2017; Lee et al., 2012); hence, MRS1220 and tranylcypromine may have eliminated mitochondria by activating autophagy. Ca^{2+} signaling is a well-known apoptosis and autophagy regulator. However, the effects of flunarizine on autophagy or apoptosis have not yet been elucidated; therefore, further experiments are required for elucidating the mechanisms of the effects of the candidate drugs. Moreover, mitochondrial dysfunction plays a pivotal role in the pathogenesis of other neurodegenerative diseases, such as Alzheimer disease (Fang et al., 2019), Huntington disease, and amyotrophic lateral sclerosis (Rogers et al., 2017). Therefore, our proposed system could also be useful in detecting disease phenotypes and screening therapeutic drugs in these neurodegenerative diseases.

Considering the clinical use of these drugs for treatment of PD, TCP-FA4, a derivate of tranylcypromine (Desino et al., 2009), flunarizine (Agarwal et al., 1996), and bromocriptine (Friis et al., 1979), have good blood-brain barrier permeability. Caffeine, a nonselective adenosine receptor antagonist, has been reported to lower the risk of PD (Altman et al., 2011; Postuma et al., 2012). Currently, an adenosine A2A receptor antagonist is used as treatment for PD, but A3A-R antagonists have not yet been used. Tranylcypromine is used as an antidepressant, and other MAO inhibitors are currently used as a treatment for PD. Further experiments are needed to determine whether this effect on mitochondria is unique to tranylcypromine. Flunarizine is effective in treating migraine but has side effects, such as depression and weight gain (Sørensen et al., 1991). CCBs can induce parkinsonism (Jhang et al., 2017), but some reports suggested that CCBs lower the risk of PD (Jhang et al., 2017; Simon et al., 2010; Swart and Hurley, 2016). The ergot-derived dopamine agonists were commonly used as a treatment for PD, but lots of adverse events, such as valvar heart disease, retroperitoneal fibrosis, pleurisy, and pericarditis have been reported (Horvath et al., 2004; Schade et al., 2007). Moreover, because we used these candidate compounds as treatment to neurons and flies in progressive phase, their efficacies can be expected from the prodromal to mid-term stage of PD. Overall, these four compounds have potential as disease-modifying treatment for PD, and the elucidation of their mechanisms of action, including their target molecules and pathways, could lead to the discovery of novel clinically optimized drugs.

The efficacy of the candidate drugs was validated using both *in vivo* and *in vitro* models as summarized in Table 1. We first confirmed their efficacies and optimal concentrations in PARK2 and PARK6 iPSCs. Then we applied them to *PINK1* RNAi flies and evaluated their effect on locomotion activity and ATP production to confirm their

(D) Compound evaluation in mitochondrial clearance assay. Data represent the ratio of mitochondrial area in neurons treated with CCCP + compound and that in neurons treated with CCCP + DMSO (n = 6 independent replicates; mean ± SEM). *$p < 0.05$ compared with DMSO by Wilcoxon signed rank test.
(E) Compound evaluation in apoptosis assay. Data represent the ratio of fluorescence intensity of cleaved caspase-3 in neurons treated with CCCP + compound and that in neurons treated with CCCP + DMSO (n = 6 independent replicates; mean ± SEM). *$p < 0.05$ compared with CCCP + DMSO by Wilcoxon signed rank test. n.s., not significant. See also Figure S6.

Table 1. Summary of the Therapeutic Effect of Candidate Drugs in Various PD Models Used in This Study

	PARK2A			PARK2B			PARK6			Idiopathic PD		*Drosophila*		
	Apoptosis (Basal)	Apoptosis (+CCCP)	Mito phagy	Apoptosis (Basal)	Apoptosis (+CCCP)	Mito phagy	Apoptosis (Basal)	Apoptosis (+CCCP)	Mito phagy	Apoptosis (Basal)	Mito phagy	Move ment	ATP	Mitochondrial Reduction
MRS 1220	–	+	+	–	+	+	–	+	+	–	–	+	–	–
Tranylcypromine	–	–	+	+	+	–	+	–	+	–	+	–	–	NA
Flunarizine	–	+	+	+	+	+	+	–	+	+	+	–	–	NA
Bromocriptine	–	–	+	+	+	+	+	+	+	+	+	+	+	+

therapeutic effects (Yang et al., 2006). The inactivation of PINK1 in *Drosophila* causes severe defects in mitochondrial morphology, muscular function, and dopaminergic neurons (Yang et al., 2006). The loss of Parkin in *Drosophila* produces very similar phenotypes with the inactivation of PINK1 (Greene et al., 2003; Shiba-Fukushima et al., 2017). We used *Drosophila* larvae expressing *PINK1* RNAi in muscles as a mitochondria-associated PD animal model for the following reasons: first, the reduced but not complete loss of PINK1-Parkin pathway activity would mimic most of the pathological conditions of PD caused by mitophagy defects, including those of PARK2 and PARK6 cases harboring milder mutations; secondly, controlling the amount of drug administered to *Drosophila* larvae is easy due to their stable feeding behavior; thirdly, their muscular mitochondrial phenotype is detected at an early stage and appears to be more severe than that of dopaminergic neurons. On the other hand, there are some limitations to the use of *Drosophila* as human disease models. It is difficult to evaluate the effects of compounds in aged animals using PD model flies, which show early developmental phenotypes, such as *PINK1* RNAi flies, because flies do not consume any food or water for 3.5–4.5 days during pupation, making it difficult to administer compounds constantly. Evaluation of the compounds for the survival and function of dopaminergic neurons in our fly models would be a challenge for further study. Nevertheless, *Drosophila* larval models have advantages in evaluating compounds targeting mitochondria in early screenings for drug repositioning at least as a mitochondria-associated PD model animal. The *Drosophila* genome contains at least ~75% of ortholog genes for human diseases, and most cell-signaling pathways in mammals are conserved as simple frameworks (Reiter et al., 2001). Moreover, mitochondrial degeneration caused by the defects in PINK1-Parkin signaling is more obvious than in rodent models (Clark et al., 2006; Kitada et al., 2007; Park et al., 2006; Yang et al., 2006). Thus, drug evaluation using *Drosophila* models is less expensive and can bypass ethical issues to assess approved or potential compounds in terms of highly conserved mitochondrial functions.

The majority of patients with PD are idiopathic cases with multiple pathological causes, such as impaired mitochondrial homeostasis, α-synuclein accumulation, autophagic dysfunction, endoplasmic reticulum stress, and immunological dysfunction, due to genetic and environmental factors (Kalia and Lang, 2015). In this study, we newly established iPSCs from four idiopathic PD patients, and two out of these showed increased apoptosis and impaired mitochondrial clearance (Figures 6C and 6D). Unexpectedly, both lines had *LRRK2*-p.G2385R (heterozygote), which has been reported as a risk variant for PD (Di Fonzo et al., 2006; Tan et al., 2007). Because G2385R was found in approximately 5% of the control in an Asian population, this variant is not a pathogenic mutation (Funayama et al., 2007). The association of this variant with mitochondrial function has not yet been reported. It is unclear whether our proposed system can detect unknown genetic mutations, or whether these mutations are not relevant to the increment of apoptosis and impairment of mitochondrial clearance; therefore, further investigation with more samples is required. Interestingly, the identified drug candidates were effective in only one of two cases showing the phenotypes but ineffective in the other, indicating that, although a certain number of idiopathic cases show mitochondrial clearance abnormalities, the drugs we identified in PARK2/6 have limited efficacies. In conclusion, we were still able to show that our high-throughput phenotype detection system with familial PD neurons could be beneficial for patients with idiopathic PD having abnormal phenotypes common to familial PD.

EXPERIMENTAL PROCEDURES

Culture of Human iPSCs

The control human iPSC lines 201B7 (Takahashi et al., 2007) and WD39 (Imaizumi et al., 2012), PARK2 lines PA9 and PB20 (Imaizumi et al., 2012), and PARK6 line PKB4/6 (Shiba-Fukushima et al., 2017) were cultured on mitomycin C-treated SNL murine fibroblast feeder cells in iPSC medium as described previously (Takahashi et al., 2007). Details about the iPSC lines are given in Table S1. All experimental procedures involving human iPSCs were

approved by the Juntendo University School of Medicine Ethics Committee (approval no. 2017032).

Isolation of Human T Cells and Induction into iPSCs on a Small Scale

iPSCs were derived from four patients with idiopathic PD and three age-matched healthy controls (detailed information is given in Table S2). Sendai viral induction was performed on a small scale as reported previously (Fujimori et al., 2018; Matsumoto et al., 2016) with slight modifications. Protocols for the iPSC induction and characterization are detailed in the Supplemental Experimental Procedures.

Neural Induction

The differentiation into midbrain dopaminergic neurons was induced as reported previously (Fujimori et al., 2017; Imaizumi et al., 2015) with slight modifications and summarized in Figure S1A. In brief, 2 days after seeding iPSCs (day 0), 3 μM SB431542 (Tocris Bioscience, Avonmouth, UK), 3 μM dorsomorphin (Sigma-Aldrich), and 3 μM CHIR99021 (ReproCELL, Yokohama, Japan) were added to the iPSC medium for 5 days (days 0–5), which was replaced daily for 5 days. This is described as the CTraS method. To form neurospheres, on day 5 iPSC colonies were detached from the feeder cell layers using the Dissociation solution (ReproCELL), and then dissociated into single cells using TrypLE Select (Life Technologies, Carlsbad, CA, USA) at 37°C for 5–7 min. The dissociated and filtered (40 μm) cells were cultured at a density of 1×10^4 cells/mL in KBM Neural Stem Cell medium (Kohjin Bio) supplemented with B27 (Life Technologies), 20 ng/mL basic fibroblast growth factor (PeproTech, Rocky Hill, NJ, USA), 2 μM SB431542 (Tocris Bioscience), and 5 μM Y27632 (Wako, Osaka, Japan) in 4% O_2 atmosphere. On day 8, 3 μM CHIR99021 and 2 μM purmorphamine (Millipore, Burlington, MA, USA) were added to the culture medium. For terminal differentiation, on day 22 the neurospheres were dissociated by TrypLE Select with same protocol as day 5 and plated onto a 96-well plate (Corning, Corning, NY, USA) with poly-L-ornithine (Sigma-Aldrich) and Fibronectin (Corning) at a density of 2×10^4 cells/well. The cells were cultured in KBM Neural Stem Cell medium supplemented with B27, 20 ng/mL brain-derived neurotrophic factor (BioLegend, Sandiego, CA, USA), glial cell-derived neuotrophic factor (PeproTech), 200 μM ascorbic acid (Sigma-Aldrich), 0.5 mM dibutyryl-cAMP (Nakalai Tesque, Kyoto, Japan), 1 ng/mL TGF-β3 (BioLegend), and 10 μM DAPT (Sigma) for 10 or 17 days before analysis. CHIR99021 was added to the medium only after the dissociated cells were plated. Every 2 days, 60% of the medium was replaced with fresh medium. We used neurons cultured for 10 days (day 32; mitophagy assay and apoptosis assay induced by CCCP) or 17 days (day 39; apoptosis assay in static condition and cell population assay) and plated onto 96-well plates.

High-Content Analysis

For the cell population, mitophagy, ROS, and apoptosis assays, neurons were fixed and then stained with the antibodies listed in Table S3. The stained neurons on 96-well plates were automatically imaged by the IN Cell Analyzer 2200 imaging system (GE Healthcare) and then automatically analyzed by the IN Cell Developer Toolbox v.1.9 (GE Healthcare). An overview of the analysis is shown in Figure S2 and detailed in the Supplemental Experimental Procedures.

Compound Library

We used a commercial inhibitor library (Sigma; S990043-INH4∼7) consisting of 320 compounds.

Drosophila Genetics and Larval Assays

Drosophila lines with the following genotypes were used: UAS-mitoGFP/+, MHC-Gal4, UAS-PINK1 RNAi/+ (PINK1 RNAi), UAS-mitoGFP/UAS-LacZ RNAi, and MHC-Gal4/+ (LacZ RNAi). Eggs were laid on grape juice agar plates. The larvae were transferred to yeast chunks (0.6 g/mL distilled water), including 0.05% DMSO with or without drugs and were raised from the first-instar to the third-instar stage. Wandering is a behavior in *Drosophila* larvae before metamorphosis. Wandering larvae at late third-instar stage were used for the crawling assay. Their crawling, mitochondrial morphology, and ATP production were analyzed as described in the Supplemental Experimental Procedures.

Statistical Analysis

The data are presented as the mean ± standard error of the mean (SEM). Analysis was performed using the JMP v.13 software (SAS Institute, Cary, NC, USA). Comparisons between the groups were performed using Steel's test or Dunnett's test after one-way ANOVA and Wilcoxon rank sum test. The effect of the compound treatment was analyzed using Wilcoxon signed-rank test. p values less than 0.05 were considered statistically significant.

SUPPLEMENTAL INFORMATION

Supplemental Information can be found online at https://doi.org/10.1016/j.stemcr.2020.04.011.

AUTHOR CONTRIBUTIONS

A.Y., K.I., and W.A. conceived and designed the experiments. A.Y., K.I., T.I., K.S.-F., Y.L., M.F., and Y.I. performed the experiments and analyzed the data. S.S., T.H., A.M., Y.O., A.O., and N.H. contributed to the acquisition of patient samples and data. A.Y., K.I., Y.I., and W.A. wrote and revised the manuscript. All authors have reviewed and approved the manuscript.

ACKNOWLEDGMENTS

We thank Prof. Hideyuki Okano (Keio University, Tokyo, Japan) for providing PARK2/6 iPSCs. This work was funded by Ministry of Education, Culture, Sports, Science and Technology (MEXT), Japan-Supported Programs for the Strategic Research Foundation at Private Universities (S1411007) and the Fostering Physicians in Basic Research for Coping with Advancing Sophistication of Medicine and Medical Care, the Rare/Intractable Disease Project of the Japan (JP19ek0109244 to K.I., S.S., N.H., and W.A), Research on Development of New Drugs (JP19ak0101112 to K.I., S.S., N.H., and W.A), and the Advanced Genome Research and Bioinformatics Study to Facilitate Medical Innovation (GRIFIN, JP19km0405206s0104 to N.H.) from Japan Agency for Medical Research and Development (AMED) and the Grant-in-Aid

for Scientific Research (18K15463 to K.I., 17H04049 to Y.I., 18H02744 to S.S., and 18H04043 to N.H.) from Japan Society for the Promotion of Science (JSPS). This work was carried out (in part) at the Intractable Disease Research Center Juntendo University Graduate School of Medicine. There are no conflicts of interest to declare. We appreciate Editage (www.editage.com) for the English language editing.

Received: September 24, 2019
Revised: April 28, 2020
Accepted: April 29, 2020
Published: May 28, 2020

REFERENCES

Agarwal, V.K., Jain, S., Vaswani, M., Padma, M.V., and Maheshwari, M.C. (1996). Flunarizine as add-on therapy in refractory epilepsy: an open trial. J. Epilepsy *9*, 20–22.

Altman, R.D., Lang, A.E., and Postuma, R.B. (2011). Caffeine in Parkinson's disease: a pilot open-label, dose-escalation study. Mov. Disord. *26*, 2427–2431.

Ambrosio, S., Saccà, C.D., Amente, S., Paladino, S., Lania, L., and Majello, B. (2017). Lysine-specific demethylase LSD1 regulates autophagy in neuroblastoma through SESN2-dependent pathway. Oncogene *36*, 6701–6711.

Clark, I.E., Dodson, M.W., Jiang, C., Cao, J.H., Huh, J.R., Seol, J.H., Yoo, S.J., Hay, B.A., and Guo, M. (2006). *Drosophila* pink1 is required for mitochondrial function and interacts genetically with parkin. Nature *441*, 1162–1166.

Deng, H., Wang, P., and Jankovic, J. (2018). The genetics of Parkinson disease. Ageing Res. Rev. *42*, 72–85.

Desino, K.E., Pignatello, R., Guccione, S., Basile, L., Ansar, S., Michaelis, M.L., Ramsay, R.R., and Audus, K.L. (2009). TCP-FA4: a derivative of tranylcypromine showing improved blood-brain permeability. Biochem. Pharmacol. *78*, 1412–1417.

Devi, L., Raghavendran, V., Prabhu, B.M., Avadhani, N.G., and Anandatheerthavarada, H.K. (2008). Mitochondrial import and accumulation of α-synuclein impair complex I in human dopaminergic neuronal cultures and Parkinson disease brain. J. Biol. Chem. *283*, 9089–9100.

Fang, E.F., Hou, Y., Palikaras, K., Adriaanse, B.A., Kerr, J.S., Yang, B., Lautrup, S., Hasan-Olive, M.M., Caponio, D., Dan, X., et al. (2019). Mitophagy inhibits amyloid-β and tau pathology and reverses cognitive deficits in models of Alzheimer's disease. Nat. Neurosci. *22*, 401–412.

Di Fonzo, A., Wu-Chou, Y.H., Lu, C.S., Van Doeselaar, M., Simons, E.J., Rohé, C.F., Chang, H.C., Chen, R.S., Weng, Y.H., Vanacore, N., et al. (2006). A common missense variant in the LRRK2 gene, Gly2385Arg, associated with Parkinson's disease risk in Taiwan. Neurogenetics *7*, 133–138.

Friis, M.L., Paulson, O.B., and Hertz, M.M. (1979). Transfer of bromocriptine across the blood-brain barrier in man. Acta Neurol. Scand. *59*, 88–95.

Fujimori, K., Matsumoto, T., Kisa, F., Hattori, N., Okano, H., and Akamatsu, W. (2017). Escape from pluripotency via inhibition of TGF-β/BMP and activation of Wnt signaling accelerates differentiation and aging in hPSC progeny cells. Stem Cell Reports *9*, 1675–1691.

Fujimori, K., Ishikawa, M., Otomo, A., Atsuta, N., Nakamura, R., Akiyama, T., Hadano, S., Aoki, M., Saya, H., Sobue, G., et al. (2018). Modeling sporadic ALS in iPSC-derived motor neurons identifies a potential therapeutic agent. Nat. Med. *24*, 1579–1589.

Funayama, M., Li, Y., Tomiyama, H., Yoshino, H., Imamichi, Y., Yamamoto, M., Murata, M., Toda, T., Mizuno, Y., and Hattori, N. (2007). Leucine-rich repeat kinase 2 G2385R variant is a risk factor for Parkinson disease in Asian population. Neuroreport *18*, 273–275.

Goldberg, M.S., Fleming, S.M., Palacino, J.J., Cepeda, C., Lam, H.A., Bhatnagar, A., Meloni, E.G., Wu, N., Ackerson, L.C., Klapstein, G.J., et al. (2003). Parkin-deficient mice exhibit nigrostriatal deficits but not loss of dopaminergic neurons. J. Biol. Chem. *278*, 43628–43635.

Greene, J.C., Whitworth, A.J., Kuo, I., Andrews, L.A., Feany, M.B., and Pallanck, L.J. (2003). Mitochondrial pathology and apoptotic muscle degeneration in *Drosophila* parkin mutants. Proc. Natl. Acad. Sci. U S A *100*, 4078–4083.

Horvath, J., Fross, R.D., Kleiner-Fisman, G., Lerch, R., Stalder, H., Liaudat, S., Raskoff, W.J., Flachsbart, K.D., Rakowski, H., Pache, J.-C., et al. (2004). Severe multivalvular heart disease: a new complication of the ergot derivative dopamine agonists. Mov. Disord. *19*, 656–662.

Imaizumi, K., Sone, T., Ibata, K., Fujimori, K., Yuzaki, M., Akamatsu, W., and Okano, H. (2015). Controlling the regional identity of hPSC-derived neurons to uncover neuronal subtype specificity of neurological disease phenotypes. Stem Cell Reports *5*, 1010–1022.

Imaizumi, Y., Okada, Y., Akamatsu, W., Koike, M., Kuzumaki, N., Hayakawa, H., Nihira, T., Kobayashi, T., Ohyama, M., Sato, S., et al. (2012). Mitochondrial dysfunction associated with increased oxidative stress and α-synuclein accumulation in PARK2 iPSC-derived neurons and postmortem brain tissue. Mol. Brain *5*, 1–13.

Imamura, K., Izumi, Y., Watanabe, A., Tsukita, K., Woltjen, K., Yamamoto, T., Hotta, A., Kondo, T., Kitaoka, S., Ohta, A., et al. (2017). The Src/c-Abl pathway is a potential therapeutic target in amyotrophic lateral sclerosis. Sci. Transl. Med. *9*, eaaf3962.

Itier, J.M., Ibáñez, P., Mena, M.A., Abbas, N., Cohen-Salmon, C., Bohme, G.A., Laville, M., Pratt, J., Corti, O., Pradier, L., et al. (2003). Parkin gene inactivation alters behaviour and dopamine neurotransmission in the mouse. Hum. Mol. Genet. *12*, 2277–2291.

Jhang, K.M., Huang, J.Y., Nfor, O.N., Tung, Y.C., Ku, W.Y., Lee, C. Te, and Liaw, Y.P. (2017). Extrapyramidal symptoms after exposure to calcium channel blocker-flunarizine or cinnarizine. Eur. J. Clin. Pharmacol. *73*, 911–916.

Kalia, L.V., and Lang, A.E. (2015). Parkinson's disease. Lancet *386*, 896–912.

Kitada, T., Pisani, A., Porter, D.R., Yamaguchi, H., Tscherter, A., Martella, G., Bonsi, P., Zhang, C., Pothos, E.N., and Shen, J. (2007). Impaired dopamine release and synaptic plasticity in the striatum of PINK1-deficient mice. Proc. Natl. Acad. Sci. U S A *104*, 11441–11446.

Kondo, T., Imamura, K., Funayama, M., Tsukita, K., Miyake, M., Ohta, A., Woltjen, K., Nakagawa, M., Asada, T., Arai, T., et al. (2017). iPSC-based compound screening and in vitro trials identify a synergistic anti-amyloid β combination for Alzheimer's disease. Cell Rep. *21*, 2304–2312.

Lahiri, V., and Klionsky, D.J. (2017). Functional impairment in RHOT1/Miro1 degradation and mitophagy is a shared feature in familial and sporadic Parkinson disease. Autophagy *13*, 1259–1261.

Lee, Y.R., Wu, W.C., Ji, W.T., Chen, J., Cheng, Y.P., Chiang, M.K., and Chen, H.R. (2012). Reversine suppresses oral squamous cell carcinoma via cell cycle arrest and concomitantly apoptosis and autophagy. J. Biomed. Sci. *19*, 9.

Lücking, C.B., Dürr, A., Bonifati, V., Vaughan, J., De Michele, G., Gasser, T., Harhangi, B.S., Meco, G., Denèfle, P., Wood, N.W., et al. (2000). Association between early-onset Parkinson's disease and mutations in the parkin gene. N. Engl. J. Med. *342*, 1560–1567.

Matsuda, N., Sato, S., Shiba, K., Okatsu, K., Saisho, K., Gautier, C.A., Sou, Y.S., Saiki, S., Kawajiri, S., Sato, F., et al. (2010). PINK1 stabilized by mitochondrial depolarization recruits Parkin to damaged mitochondria and activates latent Parkin for mitophagy. J. Cell Biol. *189*, 211–221.

Matsumoto, T., Fujimori, K., Andoh-Noda, T., Ando, T., Kuzumaki, N., Toyoshima, M., Tada, H., Imaizumi, K., Ishikawa, M., Yamaguchi, R., et al. (2016). Functional neurons generated from T cell-derived induced pluripotent stem cells for neurological disease modeling. Stem Cell Reports *6*, 422–435.

Nalls, M.A., Pankratz, N., Lill, C.M., Do, C.B., Hernandez, D.G., Saad, M., Destefano, A.L., Kara, E., Bras, J., Sharma, M., et al. (2014). Large-scale meta-analysis of genome-wide association data identifies six new risk loci for Parkinson's disease. Nat. Genet. *46*, 989–993.

Narendra, D.P., Jin, S.M., Tanaka, A., Suen, D.F., Gautier, C.A., Shen, J., Cookson, M.R., and Youle, R.J. (2010). PINK1 is selectively stabilized on impaired mitochondria to activate Parkin. PLoS Biol. *8*, e1000298.

Orr, A.L., Rutaganira, F.U., de Roulet, D., Huang, E.J., Hertz, N.T., Shokat, K.M., and Nakamura, K. (2017). Long-term oral kinetin does not protect against α-synuclein-induced neurodegeneration in rodent models of Parkinson's disease. Neurochem. Int. *109*, 106–116.

Park, J., Lee, S.B., Lee, S., Kim, Y., Song, S., Kim, S., Bae, E., Kim, J., Shong, M., Kim, J.M., et al. (2006). Mitochondrial dysfunction in *Drosophila* PINK1 mutants is complemented by parkin. Nature *441*, 1157–1161.

Park, J.S., Davis, R.L., and Sue, C.M. (2018). Mitochondrial dysfunction in Parkinson's disease: new mechanistic insights and therapeutic perspectives. Curr. Neurol. Neurosci. Rep. *18*, 21.

Pickles, S., Vigié, P., and Youle, R.J. (2018). Mitophagy and quality control mechanisms in mitochondrial maintenance. Curr. Biol. *28*, R170–R185.

Postuma, R.B., Lang, A.E., Munhoz, R.P., Charland, K., Pelletier, A., Moscovich, M., Filla, L., Zanatta, D., Romenets, S.R., Altman, R., et al. (2012). Caffeine for treatment of Parkinson disease: a randomized controlled trial. Neurology *79*, 651–658.

Reiter, L.T., Potocki, L., Chien, S., Gribskov, M., and Bier, E. (2001). A systematic analysis of human disease-associated gene sequences in *Drosophila melanogaster.* Genome Res. *11*, 1114–1125.

Rogers, R.S., Tungtur, S., Tanaka, T., Nadeau, L.L., Badawi, Y., Wang, H., Ni, H.M., Ding, W.X., and Nishimune, H. (2017). Impaired mitophagy plays a role in denervation of neuromuscular junctions in ALS mice. Front. Neurosci. *11*, 473.

Schade, R., Andersohn, F., Suissa, S., Haverkamp, W., and Garbe, E. (2007). Dopamine agonists and the risk of cardiac-valve regurgitation. N. Engl. J. Med. *356*, 29–38.

Schapira, A.H.V., Cooper, J.M., Dexter, D., Clark, J.B., Jenner, P., and Marsden, C.D. (1990). Mitochondrial complex I deficiency in Parkinson's disease. J. Neurochem. *54*, 823–827.

Shiba-Fukushima, K., Ishikawa, K.I., Inoshita, T., Izawa, N., Takanashi, M., Sato, S., Onodera, O., Akamatsu, W., Okano, H., Imai, Y., et al. (2017). Evidence that phosphorylated ubiquitin signaling is involved in the etiology of Parkinson's disease. Hum. Mol. Genet. *26*, 3172–3185.

Sian, J., Dexter, D.T., Lees, A.J., Daniel, S., Agid, Y., Javoy-Agid, F., Jenner, P., and Marsden, C.D. (1994). Alterations in glutathione levels in Parkinson's disease and other neurodegenerative disorders affecting basal ganglia. Ann. Neurol. *36*, 348–355.

Simon, K.C., Gao, X., Chen, H., Schwarzschild, M.A., and Ascherio, A. (2010). Calcium channel blocker use and risk of Parkinson's disease. Mov. Disord. *25*, 1818–1822.

Sørensen, P.S., Larsen, B.H., Rasmussen, M.J.K., Kinge, E., Iversen, H., Alslev, T., Nøhr, P., Pedersen, K.K., Schrøder, P., Lademann, A., et al. (1991). Flunarizine versus metoprolol in migraine prophylaxis: a double-blind, randomized parallel group study of efficacy and tolerability. Headache J. Head Face Pain *31*, 650–657.

Swart, T., and Hurley, M.J. (2016). Calcium channel antagonists as disease-modifying therapy for Parkinson's disease: therapeutic rationale and current status. CNS Drugs *30*, 1127–1135.

Tabata, Y., Imaizumi, Y., Sugawara, M., Andoh-Noda, T., Banno, S., Chai, M.C., Sone, T., Yamazaki, K., Ito, M., Tsukahara, K., et al. (2018). T-type calcium channels determine the vulnerability of dopaminergic neurons to mitochondrial stress in familial Parkinson disease. Stem Cell Reports *11*, 1171–1184.

Takahashi, K., Tanabe, K., Ohnuki, M., Narita, M., Ichisaka, T., Tomoda, K., and Yamanaka, S. (2007). Induction of pluripotent stem cells from adult human fibroblasts by defined factors. Cell *131*, 861–872.

Takanashi, M., Li, Y., and Hattori, N. (2016). Absence of Lewy pathology associated with PINK1 homozygous mutation. Neurology *86*, 2212–2213.

Tan, E.K., Zhao, Y., Skipper, L., Tan, M.G., Di Fonzo, A., Sun, L., Fook-Chong, S., Tang, S., Chua, E., Yuen, Y., et al. (2007). The LRRK2 Gly2385Arg variant is associated with Parkinson's disease: genetic and functional evidence. Hum. Genet. *120*, 857–863.

Tysnes, O.B., and Storstein, A. (2017). Epidemiology of Parkinson's disease. J. Neural. Transm. (Vienna) *124*, 901–905.

Valente, E.M., Abou-Sleiman, P.M., Caputo, V., Muqit, M.M.K., Harvey, K., Gispert, S., Ali, Z., Del Turco, D., Bentivoglio, A.R.,

Healy, D.G., et al. (2004). Hereditary early-onset Parkinson's disease caused by mutations in PINK1. Science *304*, 1158–1160.

Yang, Y., Gehrke, S., Imai, Y., Huang, Z., Ouyang, Y., Wang, J.W., Yang, L., Beal, M.F., Vogel, H., and Lu, B. (2006). Mitochondrial pathology and muscle and dopaminergic neuron degeneration caused by inactivation of *Drosophila* Pink1 is rescued by Parkin. Proc. Natl. Acad. Sci. U S A *103*, 10793–10798.

Yoshii, S.R., Kishi, C., Ishihara, N., and Mizushima, N. (2011). Parkin mediates proteasome-dependent protein degradation and rupture of the outer mitochondrial membrane. J. Biol. Chem. *286*, 19630–19640.

Stem Cell Reports, Volume *14*

Supplemental Information

Identifying Therapeutic Agents for Amelioration of Mitochondrial Clearance Disorder in Neurons of Familial Parkinson Disease

Akihiro Yamaguchi, Kei-ichi Ishikawa, Tsuyoshi Inoshita, Kahori Shiba-Fukushima, Shinji Saiki, Taku Hatano, Akio Mori, Yutaka Oji, Ayami Okuzumi, Yuanzhe Li, Manabu Funayama, Yuzuru Imai, Nobutaka Hattori, and Wado Akamatsu

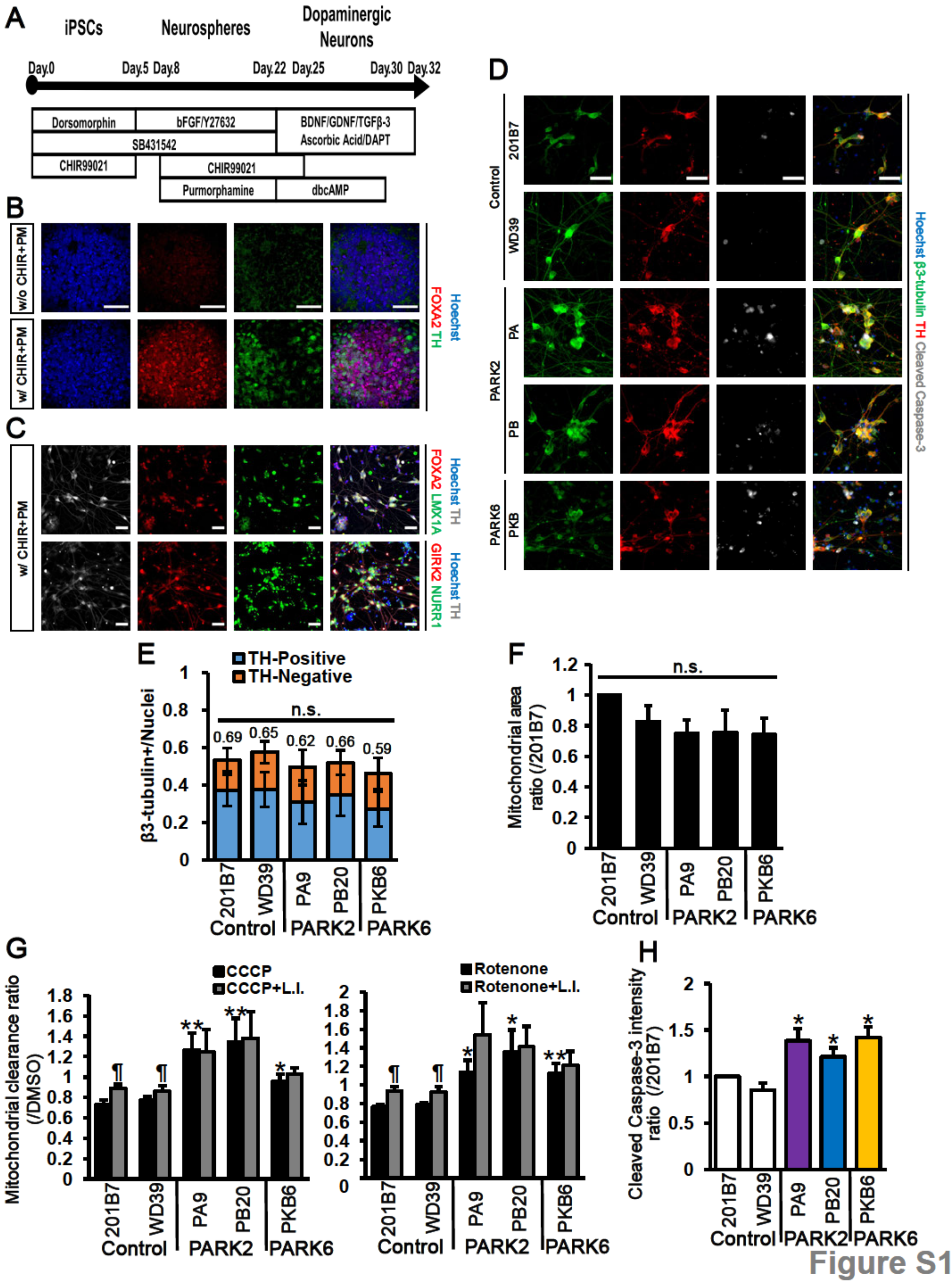
A
iPSCs
Neurospheres
Dopaminergic Neurons
Day.0
Day.5 Day.8
Day.22 Day.25
Day.30 Day.32
Dorsomorphin
bFGF/Y27632
BDNF/GDNF/TGFβ-3
Ascorbic Acid/DAPT
SB431542
CHIR99021
CHIR99021
Purmorphamine
dbcAMP
B
w/o CHIR+PM
w/ CHIR+PM
Hoechst
FOXA2 TH
C
w/ CHIR+PM
Hoechst TH
FOXA2 LMX1A
Hoechst TH
GIRK2 NURR1
D
Control
201B7
WD39
PARK2
PA
PB
PARK6
PKB
Hoechst β3-tubulin TH Cleaved Caspase-3
E
TH-Positive
TH-Negative
β3-tubulin+/Nuclei
n.s.
0.69
0.65
0.62
0.66
0.59
201B7
WD39
PA9
PB20
PKB6
Control
PARK2
PARK6
F
Mitochondrial area ratio (/201B7)
n.s.
G
CCCP
CCCP+L.I.
Rotenone
Rotenone+L.I.
Mitochondrial clearance ratio (/DMSO)
H
Cleaved Caspase-3 intensity ratio (/201B7)
Figure S1

Figure S1. Overview and Characterization of PARK2/6 Dopaminergic Neurons. Related to Figure 1

(A) Schematic of the iPSC induction into midbrain dopaminergic neurons. (B) Characterization of neurosphere treated without (w/o) or with (w/) CHIR 99021 (CHIR) and Purmorphamine (PM) from 201B7. Immunocytochemical staining was performed with antibodies against a dopaminergic neuron marker (TH) and a midbrain marker (FOXA2). Scale bar = 50 μm. (C) Characterization of differentiated neuron treated with CHIR and PM from 201B7. Immunocytochemical staining was performed with antibodies against a dopaminergic neuron marker (TH) and midbrain markers (FOXA2, LMX1A, GIRK2, and NURR1). Scale bar = 50 μm. (D) Representative images of iPSC-derived dopaminergic neurons. Scale bar = 50 μm. (E) Quantitative analysis of β3-tubulin positive ratio. The percentage above the bar indicates TH-positive ratio in β3-tubulin-positive neurons (n=4 independent replicates; mean ± SEM). n.s. means not significant by one-way ANOVA. (F) Quantitative data of basal mitochondrial area, representing the ratio of mitochondrial area of neurons as standardized by 201B7 (n=7-9 independent replicates; mean ± SEM). n.s. means not significant by one-way ANOVA. (G) Validation of the mitochondrial uncoupler and lysosome inhibitor for mitochondrial clearance assay. Data represent the ratio of mitochondrial area of neurons treated with CCCP or rotenone with/without lysosomal inhibitors (L.I.; E64d and pepstatin A; n=9 independent replicates; mean ± SEM). *$p<0.05$; *$p<0.01$ compared with 201B7, ¶$p<0.05$ comparing CCCP treatment with CCCP+L.I. treatment by Wilcoxon rank-sum test. (H) Quantitative data of spontaneous apoptosis, representing the ratio of the intensity of cleaved caspase-3 in neurons and that of control (201B7) neurons (n=4-5 independent replicates; mean ± SEM). *$p<0.05$ compared with control (201B7) by Wilcoxon rank-sum test. CCCP means carbonyl cyanide 3-chlorophenylhydrazone.

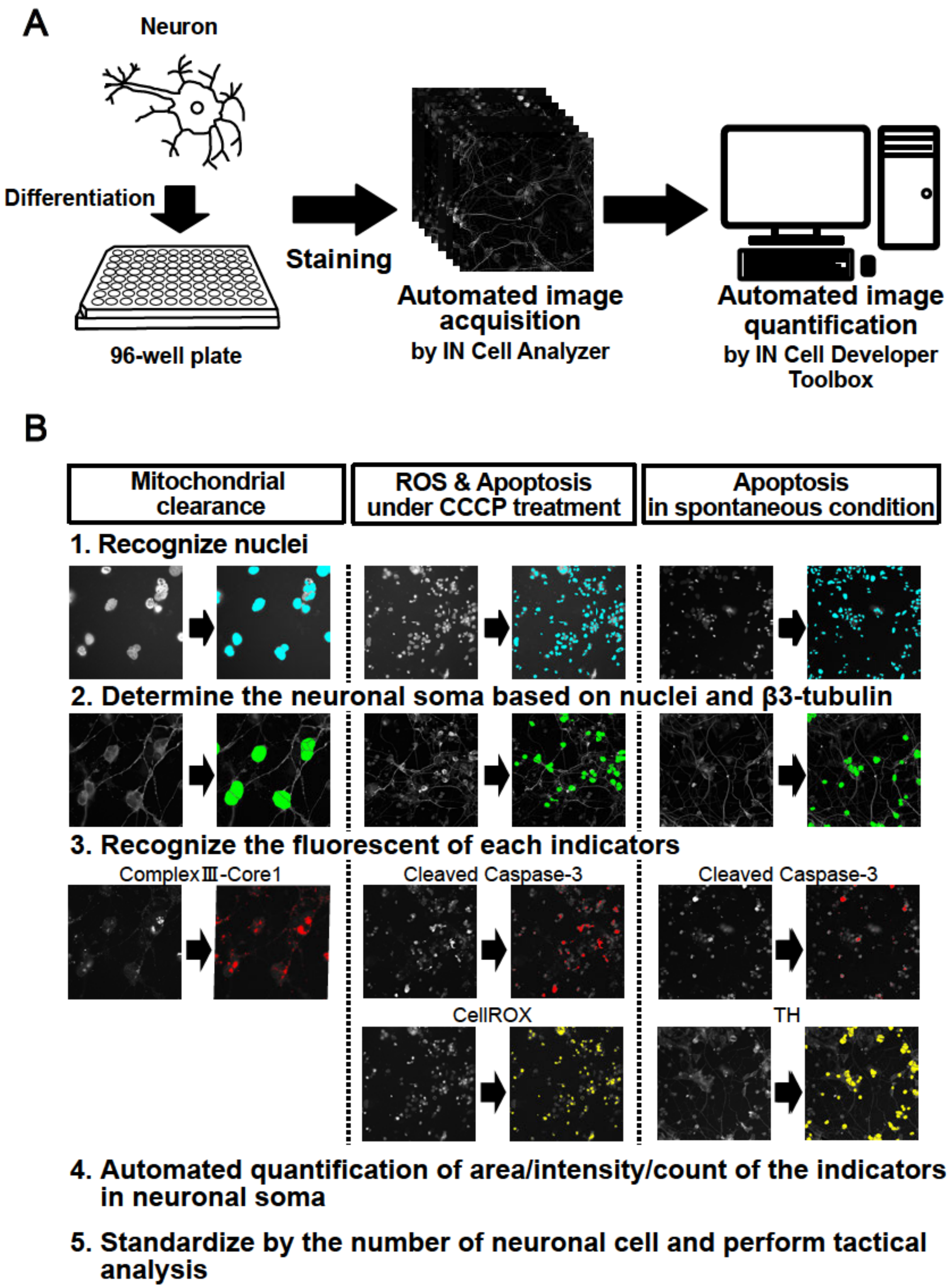
A
Neuron
Differentiation
96-well plate
Staining
Automated image acquisition
by IN Cell Analyzer
Automated image quantification
by IN Cell Developer Toolbox
B
Mitochondrial clearance
ROS & Apoptosis under CCCP treatment
Apoptosis in spontaneous condition
1. Recognize nuclei
2. Determine the neuronal soma based on nuclei and β3-tubulin
3. Recognize the fluorescent of each indicators
ComplexⅢ-Core1
Cleaved Caspase-3
Cleaved Caspase-3
CellROX
TH
4. Automated quantification of area/intensity/count of the indicators in neuronal soma
5. Standardize by the number of neuronal cell and perform tactical analysis

Figure S2

Figure S2. Schematic Representation of the Proposed Semi-Automated High-Throughput Phenotype Detection System. Related to Figure 2.

(A) Dopaminergic neurons were differentiated on 96-well plates and stained. All images were obtained automatically using the imaging cytometer and analyzed. (B) Recognition of cell features (e.g. nuclei, neural cell soma, mitochondria) and subsequent quantification of the mitochondrial area, CellROX® intensity, and cleaved caspase-3 intensity in the overlapped area were automatically analyzed. Results were standardized using the neuronal nuclei number in the field.

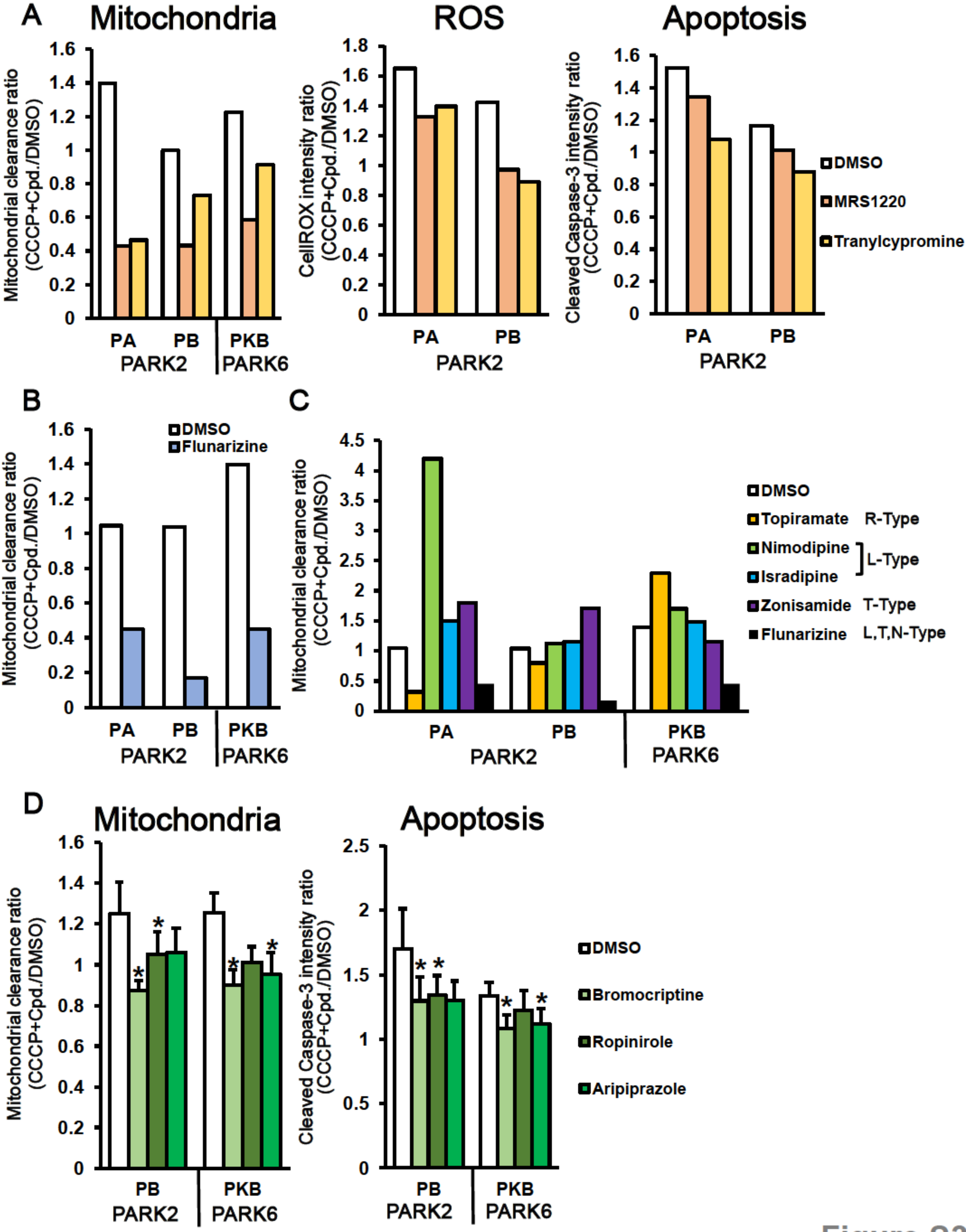
A
Mitochondria
Mitochondrial clearance ratio (CCCP+Cpd./DMSO)
1.6
1.4
1.2
1
0.8
0.6
0.4
0.2
0
PA
PB
PKB
PARK2
PARK6
ROS
CellROX intensity ratio (CCCP+Cpd./DMSO)
1.8
1.6
1.4
1.2
1
0.8
0.6
0.4
0.2
0
PA
PB
PARK2
Apoptosis
Cleaved Caspase-3 intensity ratio (CCCP+Cpd./DMSO)
1.6
1.4
1.2
1
0.8
0.6
0.4
0.2
0
PA
PB
PARK2
DMSO
MRS1220
Tranylcypromine
B
Mitochondrial clearance ratio (CCCP+Cpd./DMSO)
DMSO
Flunarizine
1.6
1.4
1.2
1
0.8
0.6
0.4
0.2
0
PA
PB
PKB
PARK2
PARK6
C
Mitochondrial clearance ratio (CCCP+Cpd./DMSO)
4.5
4
3.5
3
2.5
2
1.5
1
0.5
0
PA
PB
PKB
PARK2
PARK6
DMSO
Topiramate R-Type
Nimodipine
Isradipine
L-Type
Zonisamide T-Type
Flunarizine L,T,N-Type
D
Mitochondria
Mitochondrial clearance ratio (CCCP+Cpd./DMSO)
1.6
1.4
1.2
1
0.8
0.6
0.4
0.2
0
*
PB
PKB
PARK2
PARK6
Apoptosis
Cleaved Caspase-3 intensity ratio (CCCP+Cpd./DMSO)
2.5
2
1.5
1
0.5
0
*
PB
PKB
PARK2
PARK6
DMSO
Bromocriptine
Ropinirole
Aripiprazole

Figure S3

Figure S3. Result of Each Compound in Screening and Validation of Calcium Channel Blockers and D_2 receptor Agonists. Related to Figure 2

(A) Results of MRS1220 and tranylcypromine in compound library screening. (B) Results of flunarizine in compound library screening. (C) Results of mitochondrial clearance assay using various types of calcium channel blockers. Screening assays were similarly performed as Figure 2A. (D) Mitochondrial clearance and apoptosis assays with several D_2 receptor agonists. For the mitochondrial clearance assay, data represent the ratio of the mitochondrial area in neurons treated with CCCP and each compound and that in neurons treated with DMSO (n=9 independent replicates; mean ± SEM). For the apoptosis assay, data represent the ratio of the fluorescence intensity of the cleaved caspase-3 treated with CCCP+compound and that treated with DMSO (n=9 independent replicates; mean ± SEM). *$p<0.05$ compared with CCCP+DMSO treatment using Wilcoxon signed-rank test. CCCP means carbonyl cyanide 3-chlorophenylhydrazone.

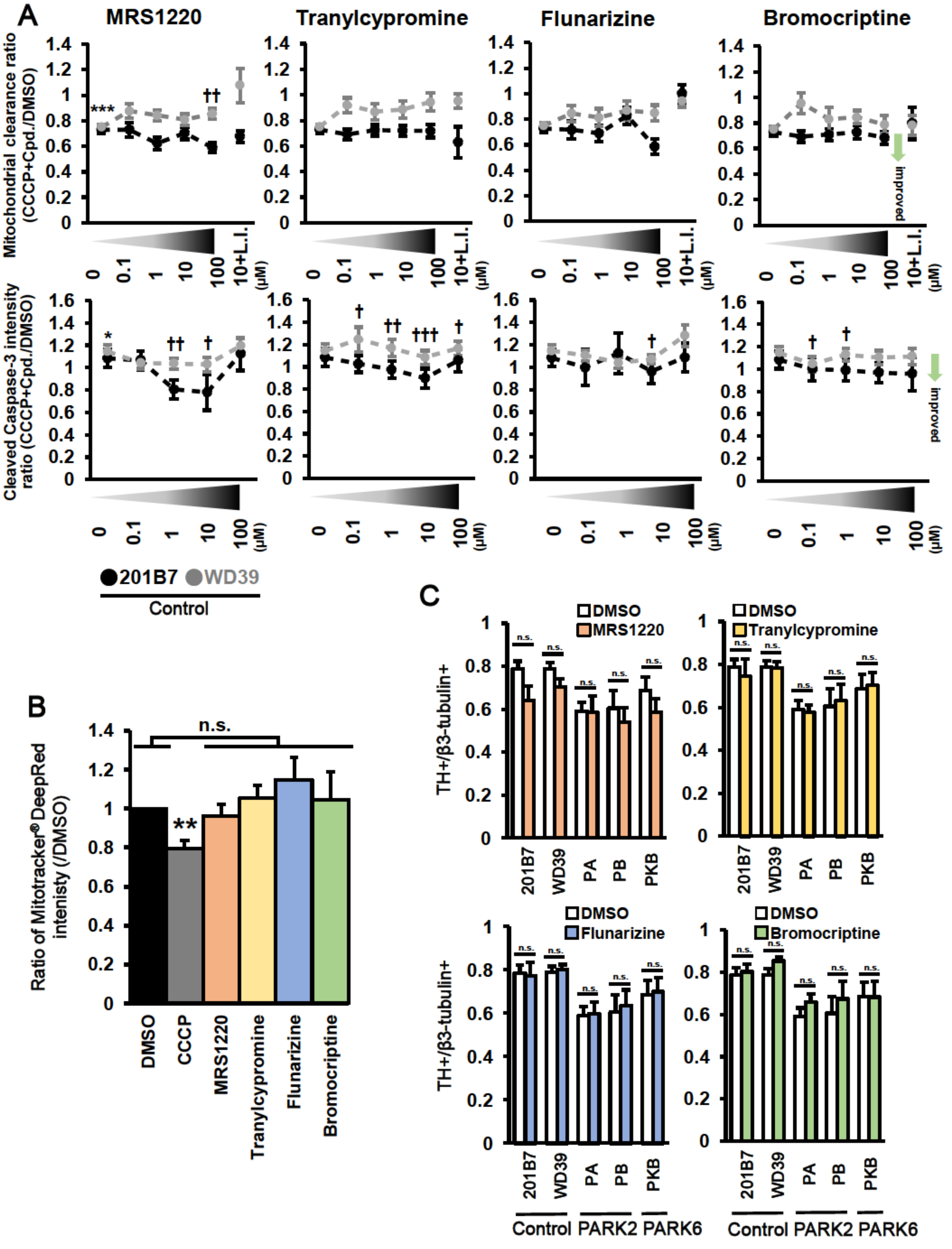
A
MRS1220
Tranylcypromine
Flunarizine
Bromocriptine
Mitochondrial clearance ratio (CCCP+Cpd./DMSO)
0 0.1 1 10 100 10+L.I. (μM)
improved
Cleaved Caspase-3 intensity ratio (CCCP+Cpd./DMSO)
0 0.1 1 10 100 (μM)
201B7 WD39
Control
B
Ratio of Mitotracker® DeepRed intenisty (/DMSO)
n.s.
DMSO
CCCP
MRS1220
Tranylcypromine
Flunarizine
Bromocriptine
C
DMSO
MRS1220
Tranylcypromine
Flunarizine
Bromocriptine
TH+/β3-tubulin+
201B7 WD39 PA PB PKB
Control PARK2 PARK6

Figure S4

Figure S4. Detailed Evaluation of Candidate Compounds. Related to Figure 3

(A) Quantitative data of candidate compounds in mitochondrial clearance and apoptosis assays in two control (201B7 and WD39) neurons. Plots show the results of DMSO, 0.1-100 μM of each candidate, and 100 μM of each candidate with lysosomal inhibitors (L.I.) under CCCP treatment. Data represent the mean ± SEM. (n=10 independent replicates). $*p<0.05$; $***p<0.001$ compared with DMSO, †$p<0.05$; ††$p<0.01$; †††$p<0.001$ compared with CCCP+DMSO by Wilcoxon signed-rank test. L.I., lysosomal inhibitors (E64d and pepstatin A). (B) Effect of candidates on mitochondrial membrane potential. Mitotracker® signal becomes weaker when mitochondrial membrane potential decreased. Data represent the ratio of fluorescence intensity of Mitotracker DeepRed® in neurons treated with DMSO and that in neurons treated with the compound (n=9 independent replicates; mean ± SEM). $**p<0.01$ compared with DMSO by Wilcoxon signed-rank test. n.s. means not significant. (C) Effect of candidates on differentiation (TH-positive ratio). Data represent the mean ± SEM. (n=9 independent replicates). n.s. means not significant by one-way ANOVA. CCCP means carbonyl cyanide 3-chlorophenylhydrazone.

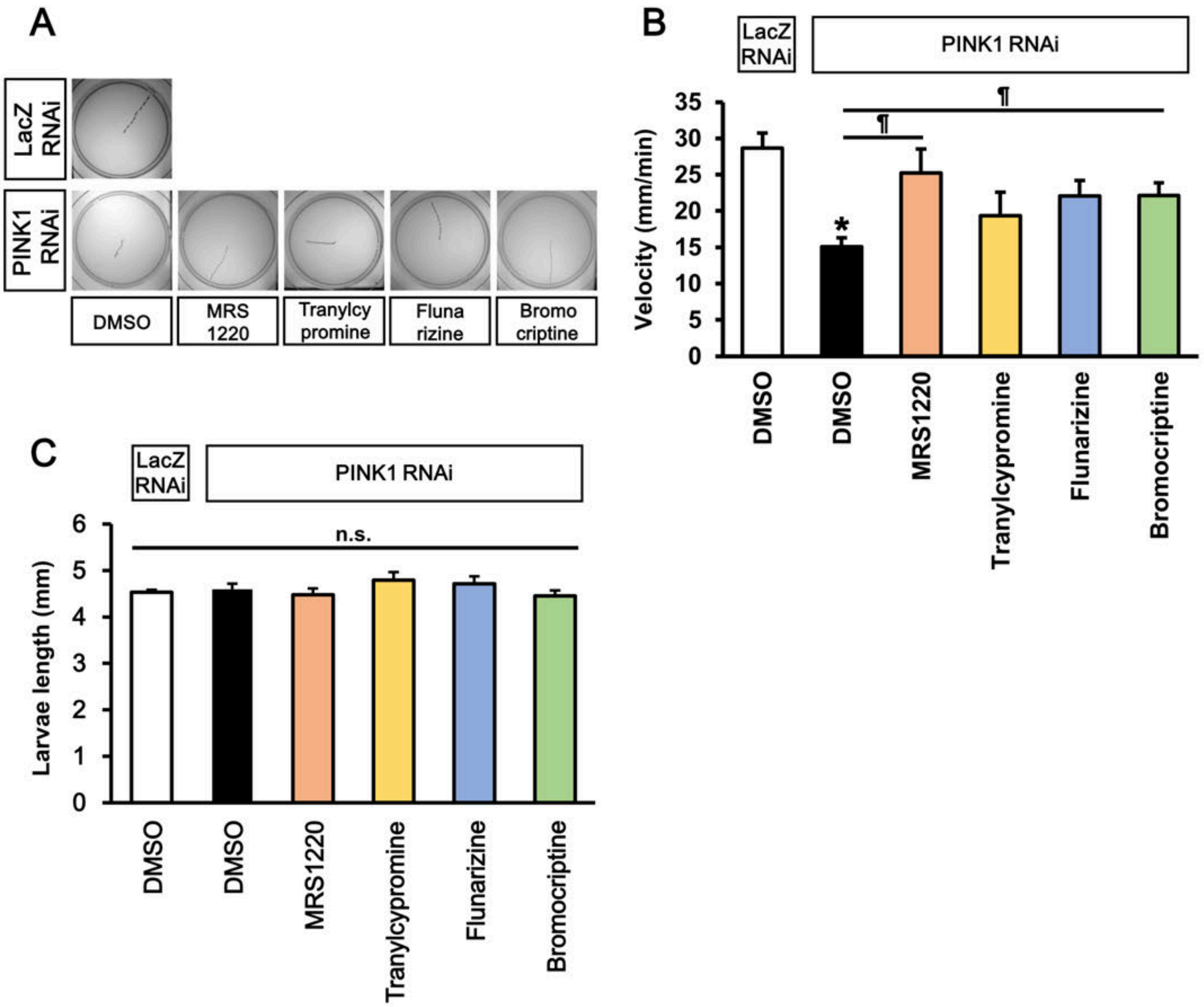
A
LacZ RNAi
PINK1 RNAi
DMSO
MRS 1220
Tranylcy promine
Fluna rizine
Bromo criptine
B
LacZ RNAi
PINK1 RNAi
Velocity (mm/min)
35
30
25
20
15
10
5
0
¶
¶
*
DMSO
DMSO
MRS1220
Tranylcypromine
Flunarizine
Bromocriptine
C
LacZ RNAi
PINK1 RNAi
Larvae length (mm)
6
5
4
3
2
1
0
n.s.
DMSO
DMSO
MRS1220
Tranylcypromine
Flunarizine
Bromocriptine

Figure S5

Figure S5. Candidate evaluation in *PINK1*-inactivated *Drosophila*. Related to Figure 5

(A) Tracking of representative larval movements treated with or without drugs. Larvae were placed at the center of dishes, and their movements recorded for 1 min. LacZ RNAi flies served as the healthy control. (B) Velocity of the movements measured in (A). Data represent the mean ± SEM (n=7-22 independent replicates). *$p<0.05$ compared with DMSO in LacZ RNAi, ¶$p<0.05$ compared with DMSO in PINK1 RNAi by Dunnett's test. (C) The body size of larvae with or without drugs. (n=7-24 independent replicates; mean ± SEM). n.s. means not significant by one-way ANOVA.

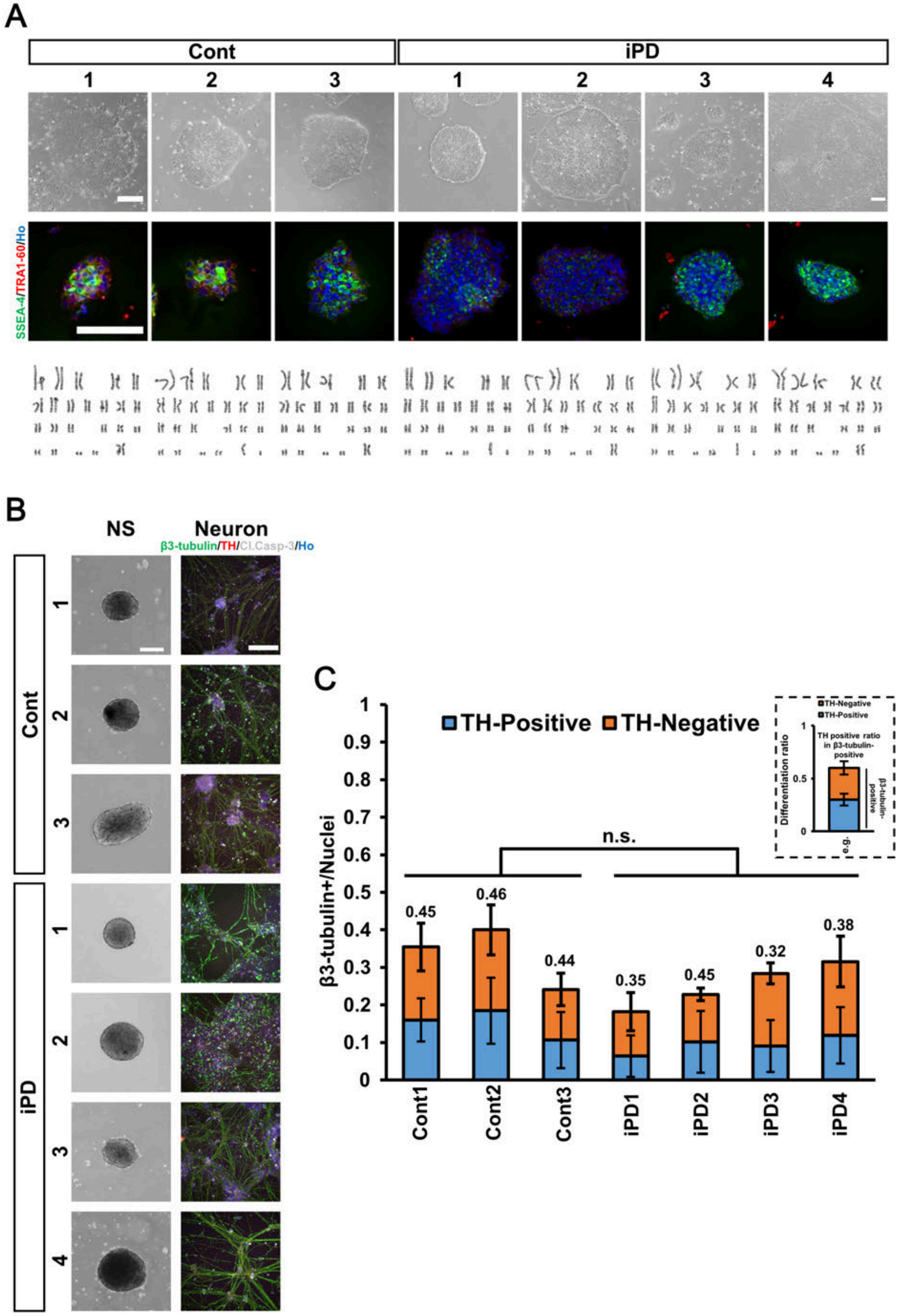

A
Cont
iPD
1
2
3
1
2
3
4
SSEA-4/TRA1-60/Ho
B
NS
Neuron
β3-tubulin/TH/Cl.Casp-3/Ho
Cont
iPD
C
TH-Positive
TH-Negative
β3-tubulin+/Nuclei
n.s.
0.45
0.46
0.44
0.35
0.45
0.32
0.38
Cont1
Cont2
Cont3
iPD1
iPD2
iPD3
iPD4
TH-Negative
TH-Positive
TH positive ratio in β3-tubulin-positive
Differentiation ratio
β3-tubulin-positive
e.g.

Figure S6

Figure S6. Characterization of Newly Established iPSCs derived from Control and Patients with Sporadic PD. Related to Figure 6

(A) Generation of iPSCs from patients with idiopathic PD and healthy controls. Representative images of iPSC colonies and their karyotypes are shown. Immunostaining of iPSCs was performed with multi-potency markers (SSEA-4 and TRA-1-60) and Hoechst. Scale bar = 200 µm. (B) Representative images of neurospheres (NS) and neurons. Immunostaining of iPSCs and neurons was performed with a neuronal marker (β3-tubulin), a dopaminergic neuron marker (TH), and Hoechst. Scale bar = 200 µm. (C) Quantitative analysis of β3-tubulin positive ratio. The percentage above the bar indicates the TH-positive ratio in β3-tubulin positive neurons (n=6-9 independent replicates; mean ± SEM). n.s. means not significant by one-way ANOVA.

Table S1. iPSCs derived from healthy donors and patients with familial PDs. Related to Figure 1.

Category	Gender	Ethnicity	Cells of Origin	Reprogramming Method	Sampling Age	Age of Onset	Risk Variants	H&Y Stage	Reference
Normal (201B7)	F	Caucasian	Dermal fibroblast	Retro virus	36	N/A	N/A	N/A	Takahashi et al. 2007
Normal (WD39)	F	Asian	Dermal fibroblast	Retro virus	16	N/A	N/A	N/A	Imaizumi et al. 2012
PARK2 (PA9)	F	Asian	Dermal fibroblast	Retro virus	72	62	*Parkin* Ex. 2-4 deletion homo	II	Imaizumi et al. 2012
PARK2 (PB20)	M	Asian	Dermal fibroblast	Retro virus	50	28	*Parkin* Ex. 6,7 deletion homo	N/A	Imaizumi et al. 2012
PARK6 (PKB4,6)	F	Asian	Dermal fibroblast	Retro virus	61	54	*PINK1* c.1162T>C homo	III	Shiba et al. 2017

Abbreviations: N/A, not available; H&Y Stage, Hoehn and Yahr stage.

Table S2. iPSCs derived from healthy donors and patients with idiopathic PDs. Related to Figure 6.

Category	Gender	Ethnicity	Cells of Origin	Reprogramming Method	Sampling Age	Age of Onset	Risk Variants	H&Y Stage
Cont1	F	Asian	PBC	Sendai virus	84	N/A	N/A	N/A
Cont2	M	Asian	PBC	Sendai virus	67	N/A	N/A	N/A
Cont3	F	Asian	PBC	Sendai virus	47	N/A	N/A	N/A
iPD1	M	Asian	PBC	Sendai virus	76	75	*LRRK2* p.G2385R hetero	III
iPD2	F	Asian	PBC	Sendai virus	66	60	N/A	I
iPD3	M	Asian	PBC	Sendai virus	66	56	*LRRK2* p.G2385R hetero	II
iPD4	F	Asian	PBC	Sendai virus	64	62	N/A	II

Abbreviations: PBC, peripheral blood cells; N/A, not available; H&Y Stage, Hoehn and Yahr stage.

Table S3. Antibodies used for staining. Related to Experimental Procedures.

Protein	Species	Source (catalogue #)	Dilution
FOXA2	Goat	Santa Cruz Biotechnology (sc-6554)	1:500
LMX1A	Rabbit	Sigma (HPA030088)	1:300
NURR1	Mouse	R&D (PP-N1404)	1:300
GIRK2	Rabbit	Alomone Labs (APC-006)	1:300
β3-tubulin	Mouse	Sigma (T8660)	1:1000
TH	Mouse	Sigma (T1299)	1:1000
TH	Rabbit	Millipore (AB152)	1:1000
Complex Ⅲ-Core1	Mouse	Sigma (459140)	1:300
TOM20	Rabbit	Santa Cruz Biotechnology (sc-11415)	1:300
Cleaved Caspase-3	Rabbit	Cell Signaling Technology (9661)	1:400
SSEA4	Mouse	Abcam (ab16287)	1:1000
TRA-1-60	Mouse	Millipore (MAB4360)	1:1000

Table S4. Compounds used for screening. Related to Experimental Procedures.

Compound	Molecular Weight	Source (catalogue #)	Concentration (reference)
DMSO	78.13	Fujifilm-Wako (048-21985)	0.3%
CCCP	204.62	Sigma (C2759)	30μM (Imaizumi et al. 2012.)
Bafilomycin A1	622.83	Sigma (B1793)	5μM (Imaizumi et al. 2012.)
E-64-d	342.43	Peptide Institute (4321-v)	30μM (Hirano et al. 2019.)
Pepstatin A	685.89	Peptide Institute (4397-v)	15μM (Hirano et al. 2019.)
Rotenone	394.42	Sigma (R8875)	10μM (Tabata et al. 2018.)
MRS 1220	403.83	Santa Cruz Biotechnology (sc-361259)	10μM
Tranylcypromine	169.65	Sigma (P8511)	10μM
Bromocriptine mesylate	750.70	Fujifilm-Wako (020-18471)	10μM
Flunarizine dihydrochloride	477.42	Sigma (F8257)	10μM
Ropinirole hydrochloride	296.84	Sigma (R2530)	10μM
Aripiprazole	448.39	Sigma (SML0935)	10μM

Supplemental Experimental Procedures

Immunocytochemistry

iPSCs, NS, or mDA neurons were fixed with 4% paraformaldehyde (PFA) in PBS at 23 °C for 30 min. The fixed mDA neurons in 96-well plates were washed with PBS automatically by a plate washer (HydroSpeed plate washer; Tecan) in the following steps. The cells were blocked with 5% FBS and 0.3% Triton X-100 in PBS for 30 min and then stained with the primary antibodies at 4 °C overnight (Table S3). After rinsing with PBS, the cells were incubated with species-specific Alexa Fluor 488-, Alexa Fluor 555-, or Alexa Fluor 647-conjugated secondary antibodies (1:500; ThermoFisher) and Hoechst 33258 (1:5000; Sigma) at 23 °C for 1 h (iPSC and mDA neurons) or overnight (NS). For whole mount image acquisition of NS, the stained samples were treated with a clearing solution of 60% glycerol and 2.5 M fructose in distilled H_2O (Dekkers et al., 2019) for 20 min. Then, the cleared samples were gently placed in microscope slides and mounted by the clearing solution. The images were obtained using a BZ-Z810 microscope (Keyence, Osaka, Japan) and IN Cell Analyzer 2200 imaging system (GE Healthcare, Chicago, IL, USA).

High-content analysis

For the cell population, mitophagy, ROS, and apoptosis assays, the stained neurons were imaged on 96-well plates by the IN Cell Analyzer 2200 imaging system (GE Healthcare). Twenty-five fields were automatically collected from each well using a 20× magnification for the cell population, ROS, and apoptosis assays, whereas 50 fields were collected from each well using a 60× objective for the mitophagy assay. The images were analyzed by IN Cell Developer Toolbox v1.9 (GE Healthcare). The nuclei, neuronal cell bodies, dopaminergic neurons, and mitochondria were identified with Hoechst, β3-tubulin, TH, and Complex III-Core 1 staining, respectively. Apoptosis was detected by cleaved caspase-3 staining. To analyze the phenotypes in neurons, the stained area of Complex III-Core 1 (mitochondrial clearance assay), and the fluorescence intensities of CellROX® (ROS assay) and cleaved Caspase-3 (apoptosis assay) in the neural soma recognized by Hoechst and β3-tubulin and/or TH signals were analyzed. The quantified area/intensity was normalized using the number of β3-tubulin and/or TH-positive nuclei. For the cell population assay, by setting the areas of β3-tubulin- and TH-positive cells, the numbers of Hoechst-positive total nuclei, β3-tubulin–positive nuclei, and TH-positive nuclei were analyzed. For drug screening, the raw value in each plate (Vr) was corrected (Vc) to account for variations between plates using the following formula:

$$Vc = Vr \times (\{the\ average\ value\ of\ CCCP\ treatment\ per\ DMSO\ in\ all\ plates\} \div \{the\ value\ of\ CCCP\ treatment\ per\ DMSO\ in\ the\ target\ plate\})$$

The overview of the analysis is shown in Figure S2.

Mitochondrial clearance assay

For induction of mitochondrial clearance, 30 µM CCCP (Sigma), 10 µM Rotenone (Sigma), or 0.3% DMSO were added in differentiation medium without dibutyryl-cAMP for 48 h. To assess the lysosomal degradation of mitochondria, 5 µM BafA1 (Sigma), or 30 µM E-64-d (Peptide Institute, Ibaraki, Japan) plus 15 µM pepstatin A (Peptide Institute) were also added at the same time. Subsequently, the cells were fixed and stained as described in "Immunocytochemistry", and automated analysis methods were discussed in "High-content analysis". The total mitochondrial area was normalized using the total β3-tubulin–positive nuclei number, and the mitochondrial clearance were evaluated using the ratio with DMSO treatment conditions.

Oxidative stress assay

After the CCCP treatment, the neurons were incubated with the CellROX® RGreen Reagent (Life Technologies) for 30 min at 37 °C following the manufacturer's instructions. Afterwards, the cells were fixed with 4% PFA at 23 °C for 30 min and stained with antibodies as described above. The automated analysis methods were as discussed in "High-content analysis." The total CellROX® intensity in neurons was normalized using the total β3-tubulin–positive nuclei number and calculated using the ratio of the CCCP treatment and DMSO treatment.

Mitochondrial membrane potential assay

After treatment with the compound for 2.5 h, the neurons were incubated with the Mitotracker® DeepRed FM (Life Technologies), a mitochondrial membrane potential indicator (Nakahira et al., 2011), at 37 °C for 30 min according to the manufacturer's instructions. Subsequently, the cells were fixed and stained. Image acquisition and quantification were performed by the IN Cell Analyzer 2200 imaging system and IN Cell Developer Toolbox v1.9, respectively (GE Healthcare).

Crawling assay of *Drosophila* larvae

The wandering larvae fed with or without compounds were placed in the middle of a 2% agar plate (100 mm in diameter). The body size of larvae in each group was not significantly different (Figure S5C). Larval positions were recorded every 7.5 s using a webcam (BSW20KM11BK; Buffalo, Nagoya, Japan) and traced by ImageJ software. The moving distance for 1 min was calculated using the ImageJ software.

Analysis of mitochondrial morphology of *Drosophila* larvae

Z-stacked images (5-µm thick) of mito-GFP–positive perinuclear mitochondria (40-μm^2-square containing a nucleus in the center) in the third-instar larval body wall muscle cells were obtained by confocal microscopy (Leica SP5). More than 2-μm^2-aggregated mitochondria were extracted by the 'Analyze particles' tool of the ImageJ software.

ATP assay

The effect of the compounds on ATP production was analyzed using CellTiter-Glo® Luminescent Cell Viability Assay (Promega, Madison, WI, USA). After treatment with compounds, 100 µL CellTiter-Glo® Reagent was added to the cells or *Drosophila* larval homogenates in white 96-well plates, and the samples were incubated according to the manufacturer's instructions. For the ATP measurement in *Drosophila* larvae, the effects of bacterial flora in the gut were estimated with and without the digestive organs. Because the effects of bacterial flora were negligible, whole bodies were homogenized in 40 µL of homogenization buffer (6 M guanidine-HCl, 100 mM Tris, and 4 mM EDTA; pH 7.8) and centrifuged at 16,000 g. The supernatants were diluted 1:500 and 1:10 with water and subjected to measurements of ATP and protein concentrations, respectively. ATP levels were estimated as luminescence intensity measured by a microplate reader (Mithras^2 LB943; Berthold, Bad Wildbad, Germany). The luminescence intensities were standardized with the amounts of proteins measured using the Pierce BCA Protein Assay Kit (Thermo Scientific).

Isolation of human T cells and small-scale induction into iPSCs

The peripheral blood mononuclear cells (PBMCs) were obtained from 4 patients with idiopathic PD and 3 age-matched healthy donors. (Detailed information is listed in Table S2.) After centrifugation, CD3- and CD28-positive cells were selected and expanded using Dynabeads® Human T-Activator CD3/CD28 (ThermoFisher Scientific, Waltham, MA, USA) in KBM 502 medium (Kohjin Bio, Sakado, Japan) for 5–7 days. For cryopreservation, we used CELLBANKER®2 (ZENOAQ, Koriyama, Japan), and thawing was performed according to the manufacturer's instructions. After thawing, the activated T cells were transferred to a 24-well plate with 500-µL KBM 502 medium at a density of 8 x 10^4 cells/well, and then, Cytotune®-iPS 2.0 Reprogramming Kit (MOI=5; DNAVEC, Tsukuba, Japan), 0.25 µM sodium butyrate (Sigma, St. Louis, MO, USA), and 5 µM cyclic pifithrin (Sigma) were added. At 24 h post-infection, 500 µL of the medium (KBM 502 + Sodium Butyrate, cyclic Pifithrin) was added. Defining the day on which the SeV infection was performed as day 0, the cells were collected on day 3 and transferred to a new 24-well plate coated with iMatrix 511-E8 (at a density of 0.5 µg/cm^2; Nippi, Adachi-ku, Japan). On day 4, 1 mL of hiPSC medium (StemFit® AK02N; Ajinomoto, Chuo-ku, Japan) was added. After day 6, hiPSC medium was changed every other day until the colonies were visible. On day 10 until day 21, the cell-generated iPSC colonies were passaged to 6-well plates coated with iMatrix 511-E8 for subsequent maintenance and cryopreservation. We used cells at passage 5–10 for differentiation. G-band karyotyping of the established iPSCs were performed at LSI Medience Corporation (Japan).

Genetic analysis

PD-causative gene analysis was performed as previously reported (Shin et al., 2017). Briefly, PD- and dementia-associated genes, including *SNCA, Parkin, UCHL1, PINK1, DJ-1, LRRK2, ATP13A2,GIGYF2,*

HTRA2, PLA2G6, FBXO7, VPS35, EIF4G1, DNAJC6, SYNJ1, DNAJC13,CHCHD2, GCH1, NR3A2, VPS13C, RAB7L1, BST1, c19orf12, RAB39B, MAPT, PSEN1, GRN, APP, and *APOE*, were screened using high-throughput, next-generation sequencing such as Ion Torrent System (IAD103177_182; Thermo Fisher Scientific, Waltham, MA, US). The panel for sequencing was designed by Ion AmpliSeq Designer (https://www.ampliseq.com). Library preparation was performed by using an Ion AmpliSeq Kit for Chef DL8 (Thermo Fisher Scientific) and Ion Chef System (Thermo Fisher Scientific). Emulsion PCR was performed using an Ion 530 Kit-Chef, and the product was sequenced on an IonS5 Plus Sequencer with an Ion 530 Chip. Sequence alignment was performed using the Torrent Mapping Alignment Program aligner implemented in the Torrent Suite software (v5.10; Thermo Fisher Scientific).

Supplemental References

Dekkers, J.F., Alieva, M., Wellens, L.M., Ariese, H.C.R., Jamieson, P.R., Vonk, A.M., Amatngalim, G.D., Hu, H., Oost, K.C., Snippert, H.J.G., et al. (2019). High-resolution 3D imaging of fixed and cleared organoids. Nat. Protoc. *14*, 1756–1771.

Nakahira, K., Haspel, J.A., Rathinam, V.A.K., Lee, S.J., Dolinay, T., Lam, H.C., Englert, J.A., Rabinovitch, M., Cernadas, M., Kim, H.P., et al. (2011). Autophagy proteins regulate innate immune responses by inhibiting the release of mitochondrial DNA mediated by the NALP3 inflammasome. Nat. Immunol. *12*, 222–230.

Shin, S., Kim, Y., Oh, S.C., Yu, N., Lee, S.T., Choi, J.R., and Lee, K.A. (2017). Validation and optimization of the Ion Torrent S5 XL sequencer and Oncomine workflow for BRCA1 and BRCA2 genetic testing. Oncotarget *8*, 34858–34866.
